# Estimating feedforward and feedback effective connections from fMRI time series: Assessments of statistical methods

**DOI:** 10.1162/netn_a_00061

**Published:** 2019-02-01

**Authors:** Ruben Sanchez-Romero, Joseph D. Ramsey, Kun Zhang, Madelyn R. K. Glymour, Biwei Huang, Clark Glymour

**Affiliations:** Department of Philosophy, Carnegie Mellon University, Pittsburgh, PA, USA; Department of Philosophy, Carnegie Mellon University, Pittsburgh, PA, USA; Department of Philosophy, Carnegie Mellon University, Pittsburgh, PA, USA; Department of Philosophy, Carnegie Mellon University, Pittsburgh, PA, USA; Department of Philosophy, Carnegie Mellon University, Pittsburgh, PA, USA; Department of Philosophy, Carnegie Mellon University, Pittsburgh, PA, USA

**Keywords:** fMRI, Effective connectivity, Feedback networks, Directed networks, Graphical causal models

## Abstract

We test the adequacies of several proposed and two new statistical methods for recovering the causal structure of systems with feedback from synthetic BOLD time series. We compare an adaptation of the first correct method for recovering cyclic linear systems; Granger causal regression; a multivariate autoregressive model with a permutation test; the Group Iterative Multiple Model Estimation (GIMME) algorithm; the Ramsey et al. non-Gaussian methods; two non-Gaussian methods by Hyvärinen and Smith; a method due to Patel et al.; and the GlobalMIT algorithm. We introduce and also compare two new methods, Fast Adjacency Skewness (FASK) and Two-Step, both of which exploit non-Gaussian features of the BOLD signal. We give theoretical justifications for the latter two algorithms. Our test models include feedback structures with and without direct feedback (2-cycles), excitatory and inhibitory feedback, models using experimentally determined structural connectivities of macaques, and empirical human resting-state and task data. We find that averaged over all of our simulations, including those with 2-cycles, several of these methods have a better than 80% orientation precision (i.e., the probability of a directed edge is in the true structure given that a procedure estimates it to be so) and the two new methods also have better than 80% recall (probability of recovering an orientation in the true structure).

## INTRODUCTION AND BACKGROUND

At rest and at work, neuronal physiological processes in various regions of the brain influence processes in other regions. Indirect measures of the local processes, such as the BOLD signal from fMRI (Goense & Logothetis, 2008; Kahn et al., 2011; Winder, Echagarruga, Zhang, & Drew, 2017), result in time series whose statistical relations are often used as keys in attempts to recover causal relations among neuronal processes. Establishing accurate, computationally feasible statistical methods for this purpose is difficult because the measurements are of tens of thousands of mildly non-Gaussian, sometimes nonstationary times series that are noisy indirect measures of processes for which there is little possibility of direct experimental testing and only a very limited number of relevant animal studies. Recent experiments (Dubois et al., in press) approach, but do not achieve, comparing fMRI inferred models with the local effects of localized interventions on the human cortex.

The principal method of testing the accuracies of proposed statistical methods has therefore been with simulated data from biophysical models in which the generating structure is of course known. The most extensive simulation study of the accuracies of statistical methods for estimating neuronal connections from BOLD signals (Smith et al., 2011) generated simulated data from dynamic causal models (DCM) (Friston, Harrison, & Penny, 2003) and found that no methods tested were useful for determining directions of influence represented by the directed graphs in the data-generating models. Some then extant methods were not tested and several methods have since appeared that show good accuracy in most of the simulated conditions. Only 2 of the 28 conditions in the Smith et al. study involved feedback cycles represented by directed graphs with cycles.

Feedback structures in functional brain networks at the cellular level include both amplifying (excitatory) and control (inhibitory) connections, and it is prudent to consider the possibility of both kinds of connections between neuronal populations at the mesoscopic scale of voxels or clusters of voxels. The Smith et al. (2011) study had all positive connections except for one condition with inhibitory feedbacks. In addition, based on anatomical evidence, a common assumption in brain connectivity analyses is that the majority of connections between clusters of cortical voxels are bidirected (Friston, Li, Daunizeau, & Stephan, 2011; Kötter & Stephan, 2003; Zeki & Shipp, 1988), although this does not imply that both directions are effective in a particular scanning episode or in response to a particular stimulus or between pairs of individual voxels in different clusters. Effective feedback structures in a scanning session or group of sessions can be represented by cyclic-directed graphs in which each brain region is represented by a vertex or node, and a directed edge, *X* → *Y*, represents the hypothesis that *X* is a direct (relative to the set of represented regions) cause of *Y*, that is, values of *X* and *Y* would be associated were a hypothetical experiment carried out that varied *X* while controlling all other variables other than *Y*.

Of the many methods that have been proposed for recovering causal connectivity from BOLD signals with feedback, several are inappropriate for the task of recovering at least some of the directions of influence including feedback relations. Simple correlation and total partial correlation conditioning for all the rest of the measured variables as estimated by Lasso (Tibshirani, 1996) or Glasso (Friedman, Hastie, & Tibshirani, 2008) techniques are a priori inappropriate for causal inference because they produce undirected graphs whose edges do not even in principle capture causal connections. Simple correlations and total partial correlations give neither directions of influence nor actual connectivities even if the direction is ignored. If *X* → *Z* → *Y* or *X* ← *Z* → *Y*, then *X* and *Y* will be correlated and a graphical representation of “functional connectivity” will find an *X* − *Y* connection. If there are several intermediates or common causes between *X* and *Y*, their correlation coefficient can be very large even though there is no real direct causal connection between them. If, instead, *X* → *Z* ← *Y*, partial correlation of *X* and *Y* controlling for (i.e., conditioning or “partialling” on) *Z* will find an *X* − *Y* connection, and, again, if there are multiple variables that, like *Z*, are direct effects of *X* and *Y*, the partial correlation of *X* and *Y* can be very large. Correlation and total partial correlation thus cannot be trusted to give directions of influence or to give correct information about causal pathways between neuronal regions. For these reasons they will not be considered here.

Assuming independent and identically distributed (i.i.d.) data from a linear system, an asymptotically correct algorithm for estimating cyclic structures, the cyclic causal discovery (CCD) procedure (Richardson, 1996), has been available for more than 20 years. Because of limitations of complexity and accuracy with finite samples, CCD has rarely been applied, but it served to show that the identification of feedback structures from i.i.d. data is theoretically possible.

Ramsey, Sanchez-Romero, and Glymour (2014) presented and tested several procedures for estimating directions of influence from adjacencies of an undirected graph. These procedures exploit the non-Gaussianity of the BOLD signal, which is typically diminished or removed by temporal high-pass filtering (Ramsey et al., 2014). In principle, all of the non-Gaussian pairwise directionality algorithms in Ramsey et al. (2014) can detect cyclic structures when the number of variables conforming the cycle (degree of the cycle) is greater than 2, but only two of them, denoted by R1 and R4, can estimate cycles of degree 2 (indicated here as 2-cycles). Ramsey et al. (2014) tested their algorithms on simple structures of five-node ring graphs with one 2-cycle, two 2-cycles sharing a node, and two 2-cycles not sharing a node; and a more complex 10 node graph with four 2-cycles. Accuracy results show that the algorithms performed better when the sample size was increased by concatenation of datasets. Nevertheless, R1 rarely detected 2-cycles while R4 had good recall and precision for detecting 2-cycles in simple structures but lost accuracy in more complex models. A search procedure for DCMs (Friston et al., 2011), assumes that all connections are direct feedback cycles (2-cycles) in the generating network. The procedure has been tested with good results on simulated fMRI structures with six variables. For complexity reasons in the model search strategy, the method is limited to a very small number of nodes, although it remains an open question whether more efficient modifications are possible (Freenor & Glymour, 2010). Other procedures capable of inferring cyclic structures from i.i.d. data have been published but not tested on neuronal data. Itani, Ohannessian, Sachs, Nolan, and Dahleh (2010) introduced a causal search procedure based on Bayesian networks capable of inferring cyclic structures from i.i.d. data and applied it to a protein measurements dataset. To find cyclic structures, this procedure depends on the possibility of the researcher intervening experimentally to fix the value of each variable individually. Our study focuses on noninvasive fMRI data, so we do not consider this procedure here. Similarly to Ramsey et al. (2014), Mooij, Peters, Janzing, Zscheischler, and Schölkopf (2016) did a systematic analysis of pairwise directionality algorithms based on different sets of assumptions, and tested them on empirical and synthetic datasets of various kinds, but did not consider their performance with cyclic structures or neuronal data.

Most efforts to identify causal structure in neuronal systems have exploited time-lagged dependencies in BOLD time series. Some of these procedures, notably those based on Granger causality (Granger, 1969) and GIMME (Gates & Molenaar, 2012), are routinely used in many functional connectivity applications (Bellucci et al., 2017; Gates, Molenaar, Iyer, Nigg, & Fair, 2014; Juan-Cruz, Gómez, Poza, Fernández, & Hornero, 2017; Price et al., 2017; Sreenivasan et al., 2017; Zelle, Gates, Fiez, Sayette, & Wilson, 2017), and their accuracies in the presence of feedbacks deserve more extensive assessment. Granger causal methods estimate causal connections in time series by multiple regression of time-indexed variables on lagged values of variables. This autoregressive method has been supplemented by procedures for estimating “contemporaneous” causal connections by applying machine learning methods to the joint distribution of the residuals after regression (Hoover, 2005; Spirtes, Glymour, & Scheines, 2000; Swanson & Granger, 1997). On the cyclic simulations of Smith et al. (2011), different versions of Granger causality performed poorly on data from structures with 5-cycles and structures with 2-cycles. Permutation tests (Gilson, Tauste Campo, Chen, Thiele, & Deco, 2017) have been proposed for testing and refining autoregressive models, including Granger causal models, specifically applied to multiunit activity data recorded from electrode arrays in a monkey. We compared this technique in multiple simulations with Granger causal methods and other methods described below.

Adapting LISREL procedures (Jöreskog & Sörbom, 1993) to time series, Gates & Molenaar (2012) introduced the GIMME algorithm, which combines autoregressive and cyclic structural equations, model fit scoring, and group voting. In principle, it can accommodate cyclic structures of any degree. Using multiple data sets on the cyclic simulations of Smith et al. (2011), it achieved an almost perfect accuracy on the 5-cycle but low accuracy on the structure containing 2-cycles. The computational complexity of the search strategy limits GIMME to small (e.g., 15) numbers of regions of interest.

In this paper, using the same BOLD synthetic data simulator employed by Smith et al. (2011), the following lag-based methods are compared: a multivariate implementation of Granger causality (Barnett & Seth, 2014); a multivariate autoregression method with a nonparametric test (Gilson et al., 2017); the GIMME algorithm; and the Global Mutual Information Transfer (GlobalMIT) algorithm, a method based on discrete dynamic Bayesian networks (Vinh, Chetty, Coppel, & Wangikar, 2011), together with the following i.i.d.-based methods: the CCD algorithm; the Ramsey et al. (2014) non-Gaussian methods; two non-Gaussian methods proposed by Hyvärinen and Smith (2013); and a method of Patel, Bowman, and Rilling (2006). We introduce and also compare two new methods, the Fast Adjacency Skeweness (FASK) algorithm and Two-Step, which exploit non-Gaussian features of the BOLD signal in different ways. Theoretical justifications are given for the latter two algorithms. The tuning parameters of all methods are optimized on separate sets of data distinct from the test data. Our test models include feedback structures with and without 2-cycles, and amplifying and control feedbacks. The best procedures are tested on four complex models derived from experimentally determined structural connectivity of macaques from Markov et al. (2013). The realism of any simulator is at least open to question, and so we also apply our two new methods, FASK and Two-Step, to two empirical datasets, one fMRI resting-state dataset from the medial temporal lobe from 23 individuals, and one fMRI dataset for a rhyming task from 9 individuals, where results are expected to be in qualitative accord with expert opinion, and in one case where a gold standard is available for a connection and its direction.

We emphasize that the algorithms we review here are scarcely the last word on the subject. New statistical procedures appear almost monthly, often with very limited testing for accuracy in fMRI and related imaging time series. The present paper is therefore not exhaustive, and certainly will not be exhaustive as future work appears. We aim, however, besides proposing and assessing two new statistical procedures, to give accurate and informative assessments of recent and long-standing procedures some of which are currently applied in diagnostics and in research psychology. We also aim to illustrate assessments in more realistic, denser structures and with structures from animal studies.

## METHODS: DATA DESCRIPTION

### Simple Networks Simulations

The fMRI BOLD data simulations of Smith et al. (2011) are based on the DCM architecture (Friston et al., 2003), where the regions of interest are nodes embedded in a directed network, and the temporal evolution of their neuronal signals follow the linear approximation:

dz/dt=σAz+Cu (1)

where *dz*/*dt* is the temporal evolution of the neuronal signals; *σ* is a constant that controls the within and between nodes neuronal lag (it was set to create a mean neuronal lag of approximately 50 ms, and as explained in (Smith et al., 2011), this value is toward the upper end of the majority of neuronal lags generally seen, and was chosen in order to evaluate lag-based methods in a best case scenario, while remaining realistic); **A** is the directed connectivity matrix defining the causal structure between nodes; *z* are the time series of the neuronal signals of the regions of interests; and **C** is a matrix controlling how the external neuronal inputs *u* feed into the network. To simulate resting-state data, the network is not altered by external stimuli, as it would be in task fMRI experiments; Instead the *u* stimuli are modeled by a Poisson process for each of the regions. Finally, the observed BOLD signals are obtained by passing the neuronal signals *z* through a balloon nonlinear functional model relating neuronal and vascular systems:

$$\tilde{y}=g(z,\theta )$$ (2)

where $\tilde{y}$ are the BOLD signals, *z* the neuronal signals, and *θ* is a vector of parameters describing the hemodynamics of the brain vascular system.

The standard parameters of Smith et al. (2011) simulations were kept and are briefly summarized here: Values for the coefficients of the **A** matrix were sampled from a Gaussian distribution with mean = 0.5, standard deviation = 0.1 and values truncated between 0.3 and 0.7. The diagonal of the **A** matrix was set to −1 to simulate a self-decay in each node. As mentioned above, a value of the time constant *σ* was set to produce a mean neuronal lag between and within nodes of approximately 50 ms. The input matrix **C** was modeled with an identity matrix to represent that each region of interest has one exclusive neuronal input. The Poisson process generating the neuronal inputs (*u* timeseries) controls two states with mean duration of 2.5 s (up) and 10 s (down). Log-log plots and fitting of the spectral density of the simulated neuronal signals *z* show that these approximate a power-law distribution *p*(*x*) = *x*^−*a*^ with a mean exponent of *a* = 2.4, which has been observed in empirical neuronal data (He, Zempel, Snyder, & Raichle, 2010). These plots are included in Supporting Information D, Sanchez-Romero et al. (2019).

To simulate differences in the vascular response across the brain, the hemodynamic response function delay has variations across nodes of *SD* = 0.5 seconds. Gaussian measurement noise was added to the resulting BOLD signal. This implies that the *observed* BOLD signals are properly defined as:

$$y=\tilde{y}+e_{m}$$ (3)

Where $\tilde{y}$ is the measurement-error-free BOLD signal and *e*_*m*_ is the added measurement noise, in our simulations, sampled from a Gaussian distribution with mean = 0 and *SD* = 1. The average standard deviation of the measurement-error-free simulated BOLD signals $\tilde{y}$ across all our conditions is 2 (±0.43); this entails that for the final *observed* BOLD signals *y*, the signal-to-noise ratio (SNR = *SD*_signal_/*SD*_noise_) is in average 2/1, or inversely that the measurement noise standard deviation is on average 0.5 times the standard deviation of the true signal.

The scanning session time was set to 10 min, but the repetition time (TR) was reduced from 3 s to 1.20 s to reflect current acquisition protocols with higher temporal resolution, as observed in Poldrack et al. (2015). The result is an increase in datapoints of the BOLD time series from 200 to 500. As reported in Ramsey et al. (2014) the Butterworth temporal filter used in Smith et al. (2011) reduces the original non-Gaussianity of the BOLD data and puts at disadvantage search methods that leverage higher order moments of the distributions. For this reason, an FSL high-pass filter of 1/200 Hz was applied, which has shown a minimal impact on the non-Gaussianity of the BOLD signal (Ramsey et al., 2014).

**Figure 1.**
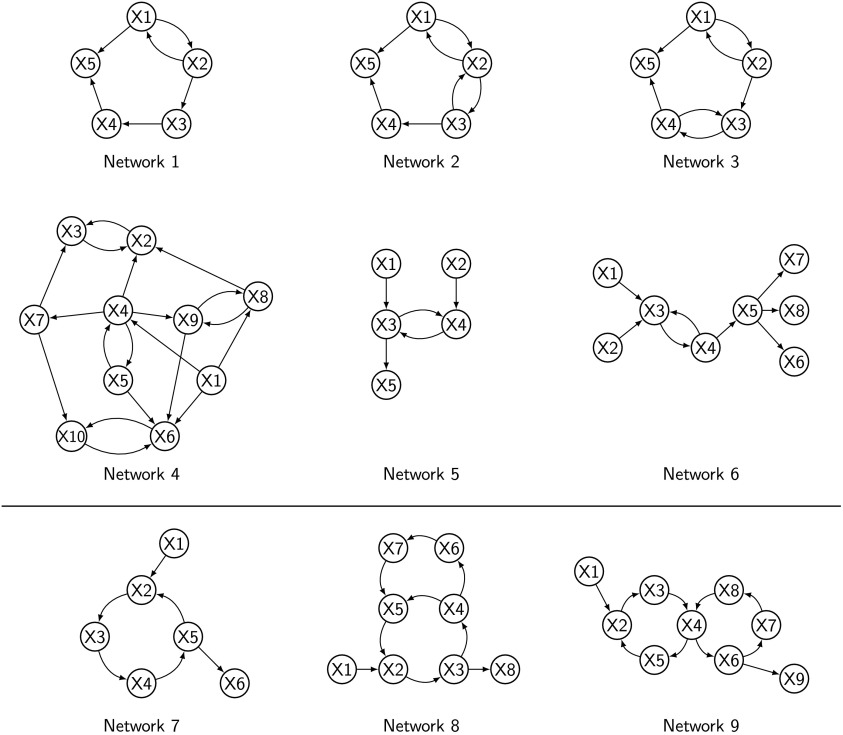
Networks containing 2-cycles: *Networks 1* to *6*; and higher degree cycles: *Networks 7* to *9*.

Using this data generator model, a series of networks containing different cyclic structures with different degrees of complexity were simulated. The networks are illustrated in Figure 1 and described below:

•  *Networks 1*, *2*, and *3* are replications of Figure 14 in Ramsey et al. (2014), with one 2-cycle, two 2-cycles sharing a node, and two 2-cycles not sharing a node.
•  *Network 4* is a replication of Figure 16 in Ramsey et al. (2014), a graph with 10 nodes, and four 2-cycles not sharing nodes.
•  *Network 5* is a 2-cycle network introduced in Richardson (1996) to illustrate the CCD algorithm; two nodes feed into a 2-cycle which in turn feeds into a single node.
•  *Network 6* is a chain network where two nodes feed into a 2-cycle which then branches into three nodes.
•  *Network 7* is a variant of Smith et al. (2011) condition 14; one node feeds into a 4-cycle which in turn feeds into a single node.
•  *Network 8* is a second-order cyclic system illustrated in the seminal work on causal analysis by Heise (1975, p. 220), useful for studying the effects of interactions between signal amplification cycles (where the product of the coefficients is positive) and signal control cycles (where the product of the coefficients is negative). The network is formed by one node that feeds into a second-order cyclic system (i.e., a cycle inside a cycle) with six nodes, which in turn feeds into a single node.
•  *Network 9* is an alternative structure for studying interactions of amplifying and control cyclic structures. One node feeds into a 4-cycle that shares one node with another 4-cycle that feeds into a single node.

Cyclic structures can amplify the signal in a system if the product of the coefficients in the cycle is positive, or control it if the product of the coefficients in the cycle is negative. Interactions (represented as node sharing) between amplification and control feedbacks can produce nonintuitive outcomes, such as unstable oscillatory signals produced by two cycles that would independently produce stable signals (Heise, 1975). Variants of the networks were made by adjusting the signs of the coefficients of the corresponding matrices to produce amplifying and control versions for *Network 5*, *6*, and *7*; and combinations of amplifying and control structures for *Network 8* and *9*. In total we simulate fMRI BOLD signals for 18 cyclic networks.

For each network, 60 different individual datasets of 500 datapoints each were simulated, which can be conceived as 60 different scanning sessions. Ten of these datasets were selected at random without replacement, centered individually, and concatenated to use as a 5,000 datapoints input for the algorithms. The scans selection, centering and concatenation process was repeated 60 times, and average performance across the 60 repetitions is reported in the Results section.

The *centering* of individual datasets before concatenating is a necessary step to avoid spurious associations due merely to differences between the individuals means of the concatenated datasets. If datasets *D*_1_ and *D*_2_ are concatenated, and variables *X* and *Y* have zero covariance in each individual dataset, so that ∑_*D*_1__ ((*X* − *μ*_*X*_)(*Y* − *μ*_*Y*_)) = 0 and ∑_*D*_2__((*X* − *μ*_*X*_)(*Y* − *μ*_*Y*_)) = 0, *unless* the means of the variables in each dataset are equal, *μ*_*X*(*D*_1_)_ = *μ*_*X*(*D*_2_)_ and *μ*_*Y*(*D*_1_)_ = *μ*_*Y*(*D*_2_)_, the resulting covariance between *X* and *Y* from the concatenated data, ∑_*D*_1_*D*_2__((*X* − *μ*_*X*_)(*Y* − *μ*_*Y*_)), will not be zero as in the individual datasets (see Pearson, Lee, & Bramley-Moore, 1899; Ramsey, Spirtes, & Glymour, 2011).

### Macaque-Based Networks Simulations

In principle, it would be straightforward to increase the complexity of the discovery problem by randomly simulating networks with more interacting excitatory and inhibitory cycles and increasing number of nodes and connections between regions of interest. A more informative way to increase the complexity and realism is to use empirical structural brain connectivity information as a blueprint. Axonal connectivity derived from tracer injection studies in nonhuman primates encode information about the directionality of the connections and so allow us to design simulations on which to test causal search algorithms. Recent studies in macaques have characterized axonal-directed connectivity networks of cortical regions spanning the whole neocortex (Markov et al., 2013, 2012). Using retrograde tracer injection experiments, Markov et al. (2013) built an axonal-directed network of 91 cortical regions and 1,615 directed edges, with cyclic structures of different degree, including 214 2-cycles. The network is publicly available (http://core-nets.org) and is used here as a blueprint to design more complex and realistic synthetic networks.

Cyclic structures are prone to produce unstable output signals under certain conditions, for example if the product of the coefficients of the connections forming the cycle is greater than one, or if certain complex interactions are present, such as a control feedback embedded in an amplifying feedback (Heise, 1975). We have noticed in simulations that most parameterizations of the directed network of Markov et al. (2013) will produce unstable synthetic outputs. To produce stable synthetic signals, small coefficient values were often required for the coefficients of the macaque empirical directed graphs. Nature may well have found larger coefficients producing stability than we have been able to find.

To simulate fMRI BOLD signals, we followed the DCM model linear parameterization as described above, except when noted. We build four different cases, described below, to study the performance of the algorithms under different conditions of connection density, number of nodes, complexity and number of cyclic paths, and linear coefficient strength. We test accuracies on the full Macaque network as reported by Markov et al. (2013) as well as subnetworks and pruned subnetworks. For each case we simulate 60 individual datasets.

•  *Macaque SmallDegree*: From the original Markov et al. (2013) macaque network we selected a subgraph from 28 target nodes where retrograde tracer injections were made, and randomly removed edges to force an average in-degree of 1.8 and out-degree of 1.8. The average in-degree and out-degree of the original macaque network is 17.7, so this considerable reduction in connectivity degree allowed us to assess the performance of the methods under sparser structures. This pruning process resulted in a subnetwork with 28 nodes, 52 directed edges and 10 cycles, out of which 5 are 2-cycles; the maximum cycle length is 6, and the mean cycle length is 3. All the 2-cycles are amplifying cycles. The coefficients were sampled from a Gaussian distribution with mean = 0.5, *SD* = 0.1, and values truncated between 0.3 and 0.7.
•  *Macaque LongRange*: The Markov et al. (2013) network reports distance in millimeters for the axonal connections. This information was used to select long-range connections, defined as the connections in the top 10 percentile of the distance distribution in the network. This percentile corresponds to a threshold of 35.6 mm (max distance 53.8 mm). Thresholding the network by using long-range connections produced a subnetwork of 67 nodes, 161 directed edges, average in-degree of 2.4 and out-degree of 2.4, and 239 cycles, out of which 19 are 2-cycles, the maximum cycle length is 12, and the mean cycle length is 7. The Markov et al. (2013) network also encodes connection weights reflecting axonal density. Base connectivity coefficients were defined for the network by mapping the values of the axonal weights for the long-range connections to values in a range between 0.05 and 0.1. Then to simulate heterogeneity, for each dataset 50% of the coefficients were randomly chosen and values sampled from a Gaussian distribution with mean = 0.01, and *SD* = 0.01, were added to them.
•  *Macaque LongRangeControl*: This case is built as for the *Macaque LongRange* case described above, with the difference that for all the 60 datasets, the same nine 2-cycles (out of 19) were defined as control cycles by setting negative one of the coefficients in the cycle.
•  *Macaque Full*: We used the complete macaque axonal connectivity dense network as reported in Markov et al. (2013): 91 nodes and 1,615 directed edges, with average in-degree of 17.7 and out-degree of 17.7 and 214 2-cycles. As in the *Macaque LongRange* simulation, base connectivity coefficients were defined for the network by mapping the values of the axonal weights for all the connections to values in a range between 0.01 and 0.05. Then to simulate heterogeneity, for each dataset 50% of the coefficients were randomly chosen and we added values sampled from a Gaussian distribution with mean = 0.01, and *SD* = 0.01, truncating the values from below to 0.01 to avoid zero or negative values.

As with the previous simulations, 60 different individual datasets of 500 datapoints were generated for each of the four macaque-based networks. Ten of these datasets were selected at random without replacement, centered individually, and concatenated to use as a 5,000 datapoints input for the algorithms. The scans selection, centering and concatenation process was repeated 60 times, and average performance across the 60 repetitions is reported in the Results section.

For simulations with very small values for the coefficients (small size effects) as these, centering the data previous to the concatenation is particularly important. As mentioned at the end of the previous section, the concatenation of data can create spurious associations due merely to differences between the means of the individual datasets. These spurious associations may be weak, but comparable in size to real effects if the true coefficients are very small (as in our simulations). Therefore, centering is necessary to reduce the chance of false positive estimations in concatenated data.

The synthetic fMRI data described above is available at http://github.com/cabal-cmu/Feedback-Discovery. The repository contains individual and concatenated datasets for each of the simple networks and Macaque-based networks, together with the corresponding graphs encoded as lists of directed edges and matrices in Matlab files.

### Empirical Data

#### Resting-state data.

To assess the algorithms under real BOLD data conditions we use publicly available high-resolution 7T human resting-state fMRI data from the medial temporal lobe. The fMRI data were acquired and preprocessed by Shah et al. (2017) and are publicly available at https://github.com/shahpreya/MTLnet. We summarize the data here and refer to Shah et al. (2017) for further details.

Resting-state fMRI data for 23 human subjects was acquired at TR = 1 s, for 7 min, resulting in 421 datapoints per subject (the original Shah et al., 2017, data contains 24 subjects, but the first one (subject S1) has 304 datapoints, so for consistency we exclude it from our analysis). The data was band-pass filter in the range 0.008–0.08 Hz, and no spatial smoothing was applied to avoid mixing of signals between neighboring regions (Shah et al., 2017). We consider the following seven regions of interest from the medial temporal lobe in each hemisphere: perirhinal cortex divided into Brodmann areas 36 and 35 (BA35 and BA36); parahippocampal cortex (PHC); entorhinal cortex (ERC); subiculum (SUB); cornu ammonis 1 (CA1); and a region comprising CA2, CA3 and dentate gyrus together (CA23DG); averaging the individual signals of these last three areas into one regional signal helps to reduce potential problems in connectivity estimation arising from the mixing of signals of neighboring areas (Smith et al., 2011), which can be especially acute in regions that are challenging to parcel out, such as CA2, CA3, and the dentate gyrus (Ekstrom et al., 2009; Preston et al., 2010).

FASK and Two-Step (Alasso) were run on 23 repetitions of 10 individual subjects concatenated (4,210 datapoints) for the seven medial temporal lobe regions of interest of the left and right hemispheres separately. The individual data was standardized (centered and variance normalized to one) before being concatenated. For comparison, we ran both algorithms on the 23 subjects datasets individually (421 datapoints). Each individual dataset was previously standardized. Estimated networks from concatenated data are reported in the Results section. Results for individual datasets are reported in Supporting Information C (Sanchez-Romero et al., 2019).

#### Task data.

To test the performance of the algorithms with fMRI task data, we use data previously published in Ramsey et al. (2010), in which nine subjects judged if a pair of visual stimuli rhymed or not. In each 20 s block, eight pairs of words were presented for 2.5 s each. Four blocks of words were followed by four blocks of pseudowords. Task blocks were separated by fixation blocks of 20 s. Data was acquired with a 3T scanner, with TR = 2 s, resulting in 160 datapoints. Raw data is available at the OpenNeuro project (https://openneuro.org/datasets/ds000003/versions/00001), and the preprocessed data used here is available at https://github.com/cabal-cmu/Feedback-Discovery. We refer the reader to Ramsey et al. (2010) for more details about the acquisition and preprocessing of the data, and region of interest definition.

For our analysis we considered eight regions of interest: left and right occipital cortex (LOCC, ROCC); left and right anterior cingulate cortex (LACC, RACC); left and right inferior frontal gyrus (LIFG, RIFG); and left and right inferior parietal (LIPL, RIPL). In addition, we included an Input variable build by convolving the rhyming task boxcar model with a canonical hemodynamic response function. If the algorithms infer orientations correctly, then edges from the Input variable must feedforward into the regions of interest, and no edge should point backward into the Input variable. This is a reliable first test that can be used to evaluate the performance of causal search algorithms on task data as shown in Ramsey et al. (2010, 2014) and Sanchez-Romero (2012).

Given the small number of subjects (9) and reduced sample size (160 datapoints), FASK and Two-Step were run on one repetition of nine individual subjects concatenated (1,440 datapoints) for the Input variable and the eight regions of interest. As with the resting-state data, the individual data was standardized before being concatenated. For comparison, we ran both algorithms on each of the nine subjects individually (160 datapoints). The datasets were standardized before running the algorithms. Estimated networks from concatenated data are reported in the Results section. Results for individual datasets are reported in Supporting Information C (Sanchez-Romero et al., 2019).

## METHODS: CYCLIC SEARCH PROCEDURES

Previously published lag-based methods and methods that assume i.i.d. data are tested here. Their principal assumptions are mentioned, and we refer the reader to the original papers for specific details. Two novel algorithms are presented that can discover cyclic structures: Fast Adjacency Skewness (FASK) and Two-Step.

Some of the procedures tested here are purely orientation algorithms and require as initial input a list of adjacencies (undirected edges), whereas others estimate a list of adjacencies as a first step followed by orientation rules. For the procedures that require it, we use the Fast Adjacency Search stable (FAS-stable) algorithm as an adjacency estimator. The FAS-stable algorithm is the order-independent adjacency search of the PC-stable algorithm that avoids spurious connections between parents of variables (Colombo & Maathuis, 2014; Spirtes et al., 2000). FAS-stable builds an undirected graph by iteratively testing conditional independence facts under increasing size of the conditioning set. We use a Bayesian information criterion (BIC; Schwarz et al., 1978) approach to perform the conditional independence tests needed by the algorithm in the following way: for two variables *X*, *Y* and a set **S** of adjacent variables of *X* or *Y*, if the BIC score for the linear model *X* ← **S** is better than the BIC score for the increased model *X* ← {*Y* ∪ **S**}, we conclude that *X* is independent of *Y* conditional on **S** (*X* ⊥ *Y*|**S**). Using the BIC score in this way we can ask if the inclusion of *Y* in the original model covariates (**S**) increases the explanatory power of the model. If this is not the case, we have evidence that *X* and *Y* are not dependent when we take into account the effect of the conditioning set **S**. The BIC score as used by FAS-stable has an additional user-input penalty discount term *c*, used to force extra sparsity on the estimated model if necessary. We defined the score here as BIC* = −2ln(*ML*) + *ck*ln(*n*), where *ML* is the maximum likelihood of the model, *c* is the extra penalty discount, *k* is the number of covariates, and *n* is the sample size. If the penalty discount *c* is set to 1, we end up with the original BIC score. We used the FAS-stable Java implementation from the Tetrad suite of algorithms (www.phil.cmu.edo/tetrad/ and www.ccd.pitt.edu/tools/), and pseudo-code for FAS-stable is included in Supporting Information A (Sanchez-Romero et al., 2019).

Each search procedure has one or more tuning parameters. As described in Supporting Information B (Section B2; Sanchez-Romero et al., 2019), the optimal tuning parameters were estimated using a subset of graphical models with a different set of model coefficients than those for the test data. Trade-offs between precision and recall in optimizing tuning parameters were decided using Matthews correlation (Matthews, 1975). The resulting optimal parameters often require very small *α* values for independence tests.

### Procedures Assuming Temporal Data

We run a multivariate implementation of Granger Causality (MVGC) by Barnett and Seth (2014; Matlab code at www.sussex.ac.uk/sackler/mvgc/). MVGC assumes the structure is linear, Gaussian, and a stationary time series. For each pair of variables *X* and *Y* in the set, MVGC computes the Granger causal multiple regressions conditioning on the rest of the variables in the set, and decides if *X* → *Y*, *X* ← *Y*, or *X* ⇄ *Y*. The order of the temporal lag for the regressions is chosen via a BIC score comparison across increasing lag models. For our BOLD simulated data the lag order chosen is always one or two, which is expected given the sampling resolution of fMRI data. MVGC as implemented by Barnett and Seth (2014), has a parameter *α* that controls the false discovery rate significance threshold (Benjamini & Hochberg, 1995) for the multiple Granger’s *F* tests. Our parameter tuning process (described in Supporting Information B, Section B1, Sanchez-Romero et al., 2019) set a value of *α* = 10^−5^. We show test data results for this parameter value.

Gilson et al. (2017) presented an alternative, which in simulation perform better than MVGC. Their method estimates the coefficients of a multivariate autoregressive (MVAR) model directly from the cross-covariance matrices using the Yule-Walker equation *Q*_1_ = *AQ*_0_, where *Q*_1_ and *Q*_0_ are the cross-covariance matrices at lag order 1 and 0, respectively, and *A* is the coefficient matrix of the MVAR model with lag order 1. This is equivalent to solving a multiple autoregression of lag order 1 for each variable on the rest of the variables in the set, including itself. The statistical significance of each coefficient *A*_*ij*_ is evaluated using a two-sided permutation test. The MVAR model is assumed to be linear, Gaussian, and stationary. Following Gilson et al. (2017) we used 1,000 permutations of the dataset, and for each permutation the variables were individually randomized to destroy any temporal dependency structure. We use an in-house Matlab implementation of the method (https://github.com/cabal-cmu/MVARp) following code provided by the authors (http://bit.ly/Gilsonetal). The tuning parameter process set a value of *α* = 10^−3^ for the two-sided permutation test. We show test data results for this parameter value and refer to the method as MVARp.

For the GIMME algorithm (Gates & Molenaar, 2012) we used the authors’ R implementation (http://CRAN.R-project.org/package=gimme). GIMME assumes linear structure and Gaussian time series data. It combines autoregressive and non-recursive structural equations, model fit scoring, and group voting to output a group graph, and then extends the graph separately for each individual dataset. Since in our simulations for each graph and parameterizations all simulated scans are alike except for chance variation, in comparing GIMME with other algorithms we use the group graph. GIMME, as implemented in R, has a single parameter for the voting step that sets the minimum frequency of appearance for an edge across the individual graphs to be included in the group graph. The tuning parameter process found two optimal values of 50% and 60%. We show test data results for the algorithm using both values, and indicate them as GIMME-50 and GIMME-60, respectively.

GlobalMIT (Vinh et al., 2011) assumes the data comes from a temporal causal process that can be modeled as a dynamic Bayesian network, allowing for self-loops. The procedure assumes linear systems and discrete multinomial data. We use the authors’ Matlab implementation (http://code.google.com/archive/p/globalmit/). GlobalMIT searches for each variable *X*, the set of parents across all the rest of the variables, **V** ∖ {*X*}, that maximize a mutual information target score. This search strategy is inefficient since with increasing number of variables, the number of combinations of possible sets of parents that have to be tested grows exponentially. In simulations, we have seen that even with 10 variables GlobalMIT can take considerable time to return. To overcome this computational problem, FAS-stable with a penalty discount of *c* = 2 was used as a preprocessor to obtain for each variable, *X*, a set of adjacent variables *adj*(*X*). We restrict the GlobalMIT parent search to *adj*(*X*) instead of **V** ∖ {*X*}. This step considerably reduces the running time of the procedure, since in general we expect |*adj*(*X*)| < |**V** ∖ {*X*}|. In our simulated structures GlobalMIT with FAS-stable preprocessing is 50 times faster than regular GlobalMIT. GlobalMIT is considered here because it has recently been applied to fMRI data from the human hippocampus recovering a well-known cyclic structure between the cornu ammonis, the dentate gyrus, and the entorhinal cortex (P. Santos et al., 2017). For our simulated continuous data, the best training results were obtained when the data was discretized into three values, the presence of self-loops was assumed (consistent with the DCM model), and an *α* value controlling the significance level for the mutual information test for independence was set to *α* = 10^−16^. We show test data performance results for these parameters and refer to the procedure as GlobalMIT (FAS).

### Procedures Assuming i.i.d. Data

A concern with the methods discussed in this paper is that they do not attempt to estimate the unobserved neuronal signal. fMRI measurements made close in time should depend on both neuronal and hemodynamic processes and variations in hemodynamic response times; vascular differences may therefore confound the two effects on the measured BOLD signal. This is a problem for lagged methods, but less so for methods that treat fMRI measurements as i.i.d. The temporal separation between recorded BOLD observations within an fMRI scan, usually between 1 and 3 s depending on the scanning protocol, should make most pairs of records from different sampling times practically independent. For example, in a 10-min session with sampling at 1.2 s, the mean time difference between time measurements will be 200 s. This independence has been shown by Dubois et al. (in press) using MyConnectome resting state data (Poldrack et al., 2015; available at http://myconnectome.org/wp/data-sharing/).

The proof of large sample correctness of the CCD algorithm (Richardson, 1996) assumes linearity and i.i.d. data. In principle, it can find cyclic structures from conditional independence relations. We used an optimized version of CCD, called CCD-max, that improves performance in simulation by enhancing the orientation accuracy of unshielded colliders (paths of the form *X* → *Z* ← *Y*, with *X* not adjacent to *Y*). This is done by finding the conditioning set **S** that confirms the conditional independence of *X* and *Y* given **S** with the best BIC* score, and orienting a collider *X* → *Z* ← *Y* if *Z* is not in the optimal conditioning set **S**. We use the Java implementation in the Tetrad suite of algorithms (www.phil.cmu.edu/tetrad/). CCD-max uses as parameter a penalty discount *c* for the BIC* score for the conditional independence decisions required by the algorithm. The penalty discount was set to *c* = 2 according to the results from the parameter tuning procedure. We show test data results for this value.

The Ramsey et al. (2014) algorithms, R1, R2, and R3 are orientation methods that assume i.i.d. non-Gaussian data. R2 and R3 assume acyclicity of the model. R1 can in principle find 2-cycles and higher degree cycles. They require as input a set of adjacencies from an undirected graph. Thus, we complemented them with the output of the FAS-stable adjacency search. R1, R2, and R3 do not require any parameter, but FAS-stable requires a penalty discount for the BIC* score for the conditional independence tests. Following the parameter tuning results, the penalty discount was set to *c* = 2 for all the methods that use FAS-stable as a first step for adjacency search. These methods are implemented in the Java Tetrad suite of algorithms (www.phil.cmu.edu/tetrad/ and www.ccd.pitt.edu/tools/) and source code is available at http://github.com/cmu-phil/tetrad. We refer to them in the Results section as FAS+R1, FAS+R2 and FAS+R3.

Skew and RSkew from Hyvärinen and Smith (2013), and Patel from Patel et al. (2006), as implemented by Smith et al. (2011), are pairwise orientation methods that assume i.i.d. non-Gaussian data. As with the Ramsey et al. (2014) methods, these algorithms require as input a set of adjacencies, so we complemented them with the output of the FAS-stable algorithm with a penalty discount of *c* = 2. Skew, Rskew, and Patel do not require any parameter. These orientation methods are also implemented in the Tetrad suite. We refer to them in the Results section as FAS+Skew, FAS+RSkew, and FAS+Patel.

#### The FASK algorithm.

The idea of the FASK algorithm is as follows: first, FAS-stable is run on the data, producing an undirected graph. We use the BIC* score as a conditional independence test with a specified penalty discount *c*. This yields undirected graph *G*_0_. The reason FAS-stable works for sparse cyclic models where the linear coefficients are all less than 1 is that correlations induced by long cyclic paths are statistically judged as zero, since they are products of multiple coefficients less than 1. Then, each of the *X* − *Y* adjacencies in *G*_0_ is oriented as a 2-cycle *X* ⇄ *Y*, or *X* → *Y*, or *X* ← *Y*. Taking up each adjacency in turn, one tests to see whether the adjacency is a 2-cycle by testing if the difference between *corr*(*X*, *Y*) and *corr*(*X*, *Y*|*X* > 0), and *corr*(*X*, *Y*) and *corr*(*X*, *Y*|*Y* > 0), are both significantly not zero. If so, the edges *X* → *Y* and *X* ← *Y* are added to the output graph *G*_1_. If not, the Left-Right orientation rule is applied: Orient *X* → *Y* in *G*_1_, if:

$$\frac{E(XY|X>0)}{\sqrt{E(X^{2}|X>0)E(Y^{2}|X>0)}}-\frac{E(XY|Y>0)}{\sqrt{E(X^{2}|Y>0)E(Y^{2}|Y>0)}}>0$$ (4)

Otherwise orient *X* ← *Y*. *G*_1_ will be a fully oriented graph. For some models, where the true coefficients of a 2-cycle between *X* and *Y* are more or less equal in magnitude but opposite in sign, FAS-stable may fail to detect an edge between *X* and *Y* when in fact a 2-cycle exists. In this case, we check explicitly whether *corr*(*X*, *Y*|*X* > 0) and *corr*(*X*, *Y*|*Y* > 0) differ by more than a set amount of 0.3. If so, the adjacency is added to the graph and oriented using the aforementioned rules.

Justification and pseudo-code for FASK are in Supporting Information A (Sanchez-Romero et al., 2019). Java source code for FASK is available at http://github.com/cmu-phil/tetrad. FASK uses two parameters, a penalty discount *c* for the BIC* score in the FAS-stable adjacency search and an *α* threshold for the test of difference of correlations in the 2-cycle detection step. Following results from the parameter tuning procedure, we set the penalty discount at *c* = 2 and *α* = 10^−6^ for the test data runs.

#### The Two-Step algorithm.

The Two-Step algorithm represents a linear causal structure, with possible unmeasured confounding variables and cyclic relationships, as:

x=Bx+Mc+e (5)

where **x** is a vector of observed variables; **B** is the directed connectivity matrix defining the causal structure between observed variables; **M** is a matrix indicating which observed variables are affected by unmeasured confounders; **c** is a vector of unmeasured confounders, if they exist; and **e** is the vector of mutually independent noise terms. Two-Step applies the principles of independent component analysis (ICA; Hyvärinen & Oja, 2000) by expressing the observed variables **x** as a mixture of a set of unobserved components:

$$\text{x}={\left(\text{I}-\text{B}\right)}^{-1}(\text{Mc}+\text{e})$$ (6)

where **I** is the identity matrix; (**I** − **B**)^−1^ defines the mixing matrix; and (**Mc** + **e**) defines the unobserved components. Generally speaking, Equation 6 does not follow the ICA model since the components defined by (**Mc** + **e**) are not necessarily mutually independent because of the possible presence of unmeasured confounders in **c**. Properly, the equation corresponds to the independent subspace analysis (ISA) model (Gruber, Gutch, & Theis, 2009; Theis, 2007). The components in (**Mc** + **e**) can be divided into mutually independent variables or groups of variables, where the variables within the same group are not independent. Each of these groups is a set of confounders plus noise terms that influence one another or are influenced by some common unknown mechanism. Under mild assumptions, the solution to ISA can be found by applying ICA and then testing for the independence between the ICA outputs. Otherwise, if there are no confounders, Equation 6 is a standard ICA model.

Equation 6 can be expressed as:

y=(I−B)x (7)

where **y** = (**Mc** + **e**). Now, independent components analysis methods aim to find a suitable matrix **B** so that the components in **y** are as independent as possible. Without constraints on **B**, we may end up with local optimal solutions, for which **B** is not necessarily the real directed connectivity matrix, or with considerable random error in the estimated **B** matrix on finite samples. To solve this problem in a computationally efficient and reliable way, the Two-Step algorithm estimates the directed connectivity matrix **B** in a divide-and-conquer manner. In a first step the algorithm infers the undirected adjacencies between the observed variables in **x**, and makes use of these adjacencies to reduce the free parameters in **B**. In a second step, the algorithm tunes the nonzero entries of **B** to make the components in **y** as independent as possible, with a sparsity constraint on **B**.

In particular, in the Two-Step algorithm, the first step finds the undirected adjacencies over the nodes **x**. If two nodes, *x*_*i*_ and *x*_*j*_, are not adjacent, then the entries *B*_*ij*_ and *B*_*ji*_ are constrained to zero in the **B** connectivity matrix. We test two different alternatives for Step 1. The first learns the undirected adjacencies using FAS-stable. The FAS-stable algorithm outputs a set of undirected adjacencies among the variables **x**. If two variables are not adjacent in the set, then the corresponding entries in the **B** matrix are constrained to zero. The second alternative learns the undirected adjacencies via Adaptive Lasso (ALasso; Zou, 2006), a regularized regression method with adapted penalization for each individual coefficient. Each node *x*_*i*_ is regressed on the rest of the nodes in **x** using ALasso. For two variables, *x*_*i*_ and *x*_*j*_, if the value of the ALasso coefficients *β*_*ij*_ and *β*_*ji*_ shrink to zero, then the *B*_*ij*_ and *B*_*ji*_ entries in the **B** connectivity matrix are constrained to zero. All the nonzero entries in **B** will be considered free parameters for the estimation of **B** in the second step of the algorithm. Step 2 estimates the free parameters in the directed connectivity matrix **B** that maximize the independence of the components of **y** = (**I** − **B**)**x**, also with a sparsity constraint on all remaining free parameters of **B**. The current implementation uses ICA with sparse connection matrices (Zhang, Peng, Chan, & Hyvärinen, 2009). Because of the sparsity constraint in Step 2, the **B** matrix entries are expected to be as small as possible. As a consequence of this constraint and initializing the free parameters for the estimation of **B** with small values, we avoid the permutation issue present in ICA-LiNGAM (Shimizu, Hoyer, Hyvärinen, & Kerminen, 2006), and in the presence of a cyclic structure the method tends to find the most stable solution for which the connectivity coefficients in the cycles are small. Finally, the **B** matrix final values can be thresholded to censor values close to zero that were not penalized in the previous steps.

We used an in-house Matlab implementation of the Two-Step algorithm available at http://github.com/cabal-cmu/Two-Step/. From the parameter tuning process described in Supporting Information B (Section B2, Sanchez-Romero et al., 2019), for Two-Step using FAS-stable we set a penalty discount of *c* = 2 for the FAS-stable step; a sparsity penalty of ln(*N*)*λ* for the **B** matrix estimation, where *N* is the sample size and *λ* was set to 64; and the absolute values of the final **B** matrix were thresholded at 0.15. We refer to the procedure as Two-Step (FAS). For Two-Step using Alasso, a penalization term equal to ln(*N*)/2 was used for the Alasso regressions; the sparsity penalty for the **B** matrix estimation was set to ln(*N*)32; and the final absolute values of the **B** matrix were thresholded at 0.15. We refer to the method as Two-Step (Alasso).

As noted above, the search procedures require at least one user-input parameter; some require two or three. To give fair comparisons under parameters optimal for the respective algorithms and kind of data, datasets were created exactly as the ones described in the above section Simple Network Simulations, but changing the coefficient values range by sampling them from a Gaussian distribution with mean = 0.4, SD = 0.1, and values truncated between 0.2 and 0.6. We refer to these data as *training* datasets. Full description of the parameter tuning process for all algorithms tested and simple and macaque-based networks is included in Supporting Information B (Sanchez-Romero et al., 2019).

## SIMULATIONS RESULTS

### Simple Networks Results

Each algorithm is parameterized as indicated in the Cyclic Search Procedures section above and its performance measures are averaged across the 18 simulated networks presented in the Simple Networks Simulations section. The performance of the algorithms was measured using Precision = True Positives/(True Positives + False Positives) and Recall = True Positives/(True Positives + False Negatives), for adjacencies, orientations and 2-cycle detection. Precision and recall range from 0 to 1. If both precision and recall are equal to 1, this indicates a perfect recovery of the true graph with no extra edges (false positives) and no missing edges (false negatives). Running time in seconds for each algorithm is also reported. All the runs were executed on a MacBook Pro, 2.8 GHz Intel Core i7 processor, 16GB of memory, with macOS Sierra 10.12.6.

The results are presented in two parts. First for the structures with 2-cycles (*Networks 1–6*) and then for the structures with higher degree cycles but not 2-cycles (*Networks 7–9*).

**Figure 2.**
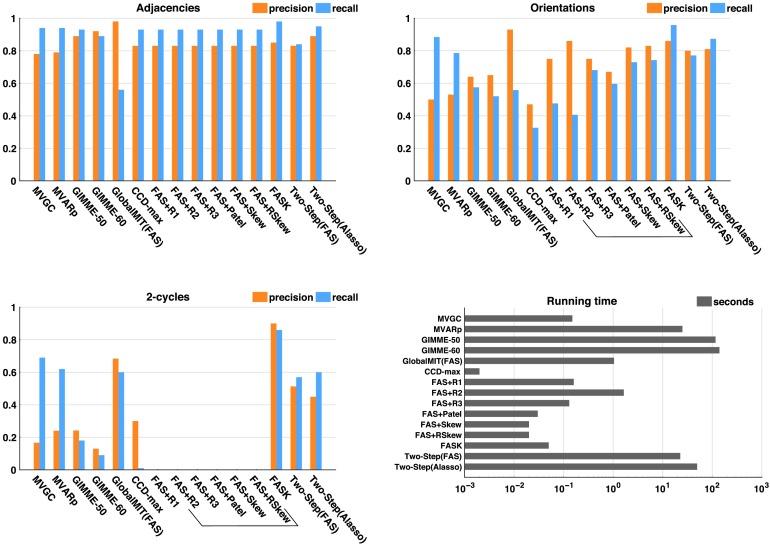
Precision and recall for adjacencies, orientations, and 2-cycles for each algorithm averaged across 10 simulations containing 2-cycles: *Network 1* to *Network 6* with amplifying and control variants. Algorithms that cannot detect 2-cycles by design are inside a bracket: R2, R3, Patel, Skew, and RSkew. Running time is in logarithmic scale. In Supporting Information C (Section C1, Sanchez-Romero et al., 2019) we disaggregate these summary results by presenting tables with average precision and recall over 60 repetitions of 10 concatenated datasets for each simulation and algorithm, with their corresponding standard deviations.

Figure 2 summarizes results by showing precision and recall averaged across the 10 simulations that contain 2-cycles: *Networks 1* to *6* with amplifying and control variants. Figure 2 bars show averages (over 10 simulations) of averages (over 60 repetitions of 10 concatenated datasets). In Supporting Information C (Section C1, Sanchez-Romero et al., 2019) we disaggregate these summary results by presenting tables with average precision and recall over 60 repetitions of 10 concatenated datasets for each simulation and algorithm tested, with their corresponding standard deviations.

**Figure 3.**
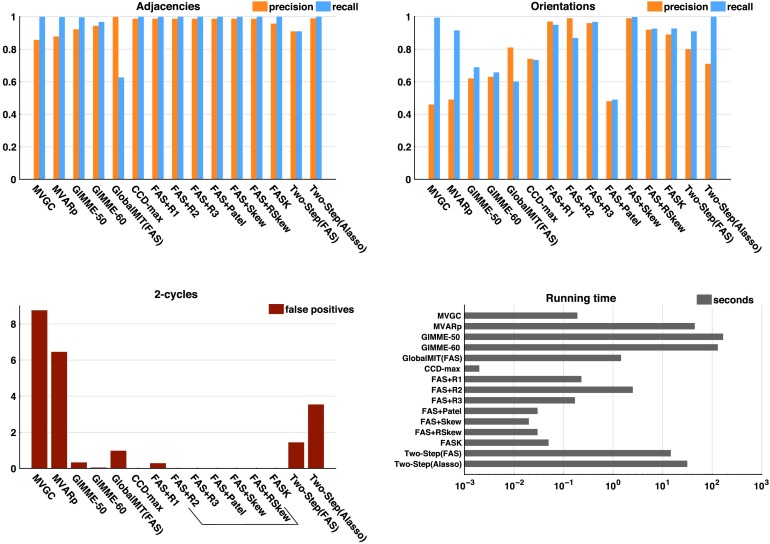
Precision and recall for adjacencies and orientations; number of 2-cycle false positives, and running times for each algorithm averaged across eight structures containing higher degree cycles but not 2-cycles: *Network 7* to *Network 9* and their amplifying and control variants. Algorithms that cannot detect 2-cycles by design are inside a bracket. Note that false positives for 2-cycles are the average number of false positives over the 60 repetitions. Running time is in logarithmic scale. In Supporting Information C (Section C2, Sanchez-Romero et al., 2019) we disaggregate these summary results by presenting tables with average precision and recall over 60 repetitions of 10 concatenated datasets for each simulation and algorithm tested, with their corresponding standard deviations.

Figure 3 summarizes results by showing precision and recall averaged across eight simulations containing higher-degree cycles but *not* 2-cycles: *Network 7* to *9* with their amplifying and control variants. In these results there is no recall for 2-cycles given that the true structures do not have 2-cycles. Instead, the average number of 2-cycle false positives is plotted to show algorithms that are prone to false detections. A bracket is drawn around those methods that by design cannot detect 2-cycles: R2, R3, Patel, Skew, and RSkew. Figure 3 bars show averages (over eight simulations) of averages (over 60 repetitions of 10 concatenated datasets). In Supporting Information C (Section C2, Sanchez-Romero et al., 2019) we disaggregate these summary results by presenting tables with average precision and recall over 60 repetitions of 10 concatenated datasets for each simulation and algorithm tested, with their corresponding standard deviations.

In simple networks containing 2-cycles and higher degree cycles (Figures 2 and 3), all methods have on average high precision and recall for adjacencies, with the exception of GlobalMIT (FAS), which has high precision but very low recall, meaning it fails to detect true adjacencies. The GlobalMIT lag-based procedure may eliminate true edges when searching for the parent set for each variable. In terms of orientations, FASK and Two-Step (Alasso) have on average the best combination of precision and recall. Of the lag-based methods, MVGC and MVARp have good recall but lower precision because of incorrectly judging many adjacencies as 2-cycles. GlobalMIT (FAS) has low orientation recall because of the low adjacency recall, but good precision. GIMME fails in both orientation precision and recall. Of the methods that can detect 2-cycles, FASK has by far the best 2-cycle precision and recall, followed by GlobalMIT (FAS). Both implementations of Two-Step have similar 2-cycle recall and precision, and MVGC and MVARp lose in precision by producing many false positive 2-cycles. Figure 3 shows that MVGC and MVARp are by far the two algorithms with the largest average number of 2-cycle false positives.

GIMME is the slowest method of all, with running times that can reach 13 min in 10 variable problems. This limitation of GIMME is probably caused by an inefficient model search strategy adapted from LISREL. The running time of MVARp is dependent on the number of permutations and with 1,000 permutations it can reach one min in 10 variable problems. Two-Step (Alasso) is also relatively slower and can take up to 2 min in 10 variable problems. Two-Step speeds up if FAS-stable is used as the first step, which indicates that FAS-stable is a faster algorithm to infer adjacencies than Alasso if the true graph is sparse. The rest of the algorithms are very fast and take milliseconds to run in lower dimension networks such as these. These running times have to be considered, taking into account the machine used (MacBook Pro, 2.8 GHz Intel Core i7 processor, 16GB memory, with macOS Sierra 10.12.6) and the various softwares used to implement the different algorithms: Java, Matlab, and R.

### Macaque-Based Networks Results

FASK and Two-Step (Alasso) were the two algorithms that achieve the best combination of precision and recall for adjacencies and orientations under the cyclic networks of Figure 1. So, their performance was analyzed under more complex networks based on macaque structural connectivity.

Both algorithms were run on 60 repetitions of 10 concatenated datasets sampled without replacement from 50 individual test datasets. Average precision and recall for adjacencies, orientations and 2-cycles, and running times in seconds are reported in Table 1. Corresponding standard deviations are in Supporting Information C (Section C3, Sanchez-Romero et al., 2019).

**Table 1.** Average over 60 repetitions of 10 concatenated datasets. Results for FASK and Two-Step (Alasso) for the four different macaque-based networks described in the Macaque-Based Networks Simulations section. Precision and recall for adjacencies (AP, AR), orientations (OP, OR), 2-cycles (2CP, 2CR), and running times in seconds (r.time)

**FASK**							
Network	AP	AR	OP	OR	2CP	2CR	r.time(s)
SmallDegree	0.78	0.84	0.75	0.79	0.96	0.81	12.97
LongRange	0.95	0.24	0.95	0.31	0.94	0.81	1.15
LongRangeControl	0.94	0.22	0.91	0.24	0.85	0.44	0.43
Full	0.98	0.01	0.99	0.02	1.00	0.07	102.25

**Two-Step (Alasso)**							
Network	AP	AR	OP	OR	2CP	2CR	r.time(s)
SmallDegree	0.74	0.82	0.52	0.76	0.18	0.83	177.79
LongRange	0.97	0.84	0.97	0.85	0.99	0.93	43.33
LongRangeControl	0.97	0.81	0.97	0.77	0.98	0.50	46.06
Full	0.88	0.27	0.82	0.28	0.77	0.46	247.46

We choose tuning parameters for the algorithms according to the procedure described in Supporting Information B (Section B2, Sanchez-Romero et al., 2019). FASK was run with a penalty discount of *c* = 1 and *α* = 10^−7^ for the *SmallDegree* network; with penalty discount *c* = 1 and *α* = 10^−1^ for *LongRange* and *LongRange Control*; and with penalty discount *c* = 2 and *α* = 10^−1^ for the *Full* network. Two-Step with Alasso was run with *λ* = 2 and **B** *threshold* = 0.10 for the *SmallDegree* network; *λ* = 2 and **B** *threshold* = 0.05 for *LongRange* and *LongRange Control*; and *λ* = 10 and **B** *threshold* = 0.005 for the *Full* network.

FASK showed excellent adjacency precision but unusably low recall in the *LongRange* and *Full* networks, probably due to the small valued coefficients used to guarantee cyclic stability in these simulations. As the coefficients values get really small the BIC* score used by the FAS-stable adjacency search loses recall but keeps its high precision. Two-Step (Alasso) achieves a precision comparable to FASK, but with a superior performance in recall. This result indicates that Alasso may be a better method than FAS-stable to detect adjacencies when coefficients are very small and the sample size is large enough.

The orientation recall for both algorithms is low due to the low adjacency recall, but their precision is excellent considering the complexity of these problems, with the exception of the *SmallDegree* case for which the orientation precision is considerably lower. Two quantitative measures of the non-Gaussianity of the data, the Anderson-Darling score and the skewness, show that the simulated data for the *SmallDegree* macaque network is more Gaussian relative to the other simulated macaque networks. This we think is the reason why both algorithms do not perform as well in orientation precision as with more non-Gaussian data.

Both FASK and Two-Step (Alasso) show high precision with lower recall for 2-cycle detection, with the exception of the *LongRangeControl* macaque network. For the *LongRangeControl* simulation, 9 out of 19 2-cycles were set as control (inhibitory) 2-cycles, with one coefficient positive and the other negative. Control 2-cycles are challenging because the interaction of the positive and negative coefficients in the cycle can produce data that make the cycle variables look independent. None of the seven tested algorithms capable of inferring 2-cycles were good at detecting control 2-cycles in our simulations (see Tables C10 and C11 in Supporting Information C (Sanchez-Romero et al., 2019) for individual results for networks with control 2-cycles).

Consistent with the running times of the previous simple networks, FASK is considerably faster than Two-Step (Alasso), sometimes by an order of magnitude. For the dense network of 91 nodes and 1,615 edges (*Full* macaque network), FASK took 1.7 min, and Two-Step (Alasso) 4.1 min on average to return. These are excellent running times considering the complexity of the structure. One way to improve the recall of FAS-stable is to increase the sample size. We explore sample size effects by concatenating increasing numbers of datasets for the *LongRange* network and present results for FASK in Table 2. Results are for one repetition of 10, 20, 30, and 40 subjects concatenated. For comparison we also include results for Two-Step (Alasso). Both algorithms were run with the aforementioned parameters. Table 2 shows that indeed, FASK adjacency and orientation recall can be improved by increasing the sample size, without detriment for the precision. Sample size increase improvements also work for Two-Step, but in lesser degree.

**Table 2.** Results for FASK and Two-Step with Alasso for increasing sample size under *LongRange* simulation, which has very small coefficients for the linear interactions. Results for one repetition for different numbers of concatenated test datasets. Precision and recall for adjacencies (AP, AR), orientations (OP, OR), 2-cycles (2CP, 2CR), and running times in seconds (r.time).

**FASK**							
Sample size	AP	AR	OP	OR	2CP	2CR	r.time (s)
5,000 (10 datasets)	0.97	0.26	0.98	0.31	1	0.68	3.33
10,000 (20 datasets)	1	0.40	0.99	0.47	0.95	0.95	17.53
15,000 (30 datasets)	1	0.51	0.99	0.57	0.95	1	162.03
20,000 (40 datasets)	1	0.58	0.97	0.63	0.86	1	1354.78

**Two-Step (Alasso)**							
Sample size	AP	AR	OP	OR	2CP	2CR	r.time (s)
5,000 (10 datasets)	0.97	0.85	0.97	0.86	1	0.90	37.48
10,000 (20 datasets)	1	0.89	1	0.89	1	0.90	77.00
15,000 (30 datasets)	1	0.92	1	0.93	1	1	81.00
20,000 (40 datasets)	1	0.94	1	0.95	1	1	117.48

### Sensitivities to Measurement Noise and Temporal Undersampling

#### Measurement noise sensitivity.

As mentioned in the description of the synthetic data, following Smith et al. (2011), all the simulations have Gaussian noise added to the BOLD signals to model measurement noise in the observations, such that *y* = $\tilde{y}$ + *e*_*m*_, where $\tilde{y}$ is the measurement-noise-free BOLD signal and *e*_*m*_ is the measurement noise distributed as a standard Gaussian with mean = 0 and *SD* = 1. For this section we use data from *Network 4*, in which the measurement-noise-free data $\tilde{y}$ has an average standard deviation of 2.5 (±0.41), implying that the final *observed* BOLD data *y* has an average SNR (*SD*_signal_/*SD*_noise_) of 2.5/1.

In principle, for each of the causal inference methods presented, the corresponding estimator was derived by minimizing some error or maximizing the likelihood of a model. To do so one has to assume a generative model, and the assumed generative models do not contain measurement noise. So, adding measurement noise will be a violation of the model assumptions. In practice, some causal discovery methods will not have a good performance when model assumptions are violated, others may be more robust, and this is why simulation studies in which model assumptions are violated are conducted to assess the robustness of causal inference algorithms. Scheines and Ramsey (2016) assess the effect of increasing magnitudes of measurement noise on the performance of the Fast Greedy Equivalence Search (FGES) causal inference algorithm (Ramsey, Glymour, Sanchez-Romero, & Glymour, 2017), and report a reduction on adjacency and orientation precision and recall, as the measurement noise becomes more prominent. They also note that the detrimental effect on accuracy of measurement noise can be partially mitigated if the sample size is increased. Zhang et al. (2017) present a formalization of a generative model with added measurement noise and derive sufficient conditions under which the causal model governing the true measurement-noise-free variables can be identified. In general, the problem of additive measurement noise arises from the fact that observed variables with measurement noise are not exact proxies of the true measurement-noise-free underlying variables and thus, the structural equations and conditional independence relations governing the true variables will not necessary hold for the observed variables. Zhang et al. (2017) and Scheines and Ramsey (2016) show how, given the difference between true and observed variables due to measurement noise, significant correlations that hold in the true variables may disappear in the observed variables (increasing the risk of false negative edges), and zero partial correlations that hold in the true variables may become significant in the observed variables (increasing the risk of false positive edges), as the magnitude of the measurement noise increases.

**Figure 4.**
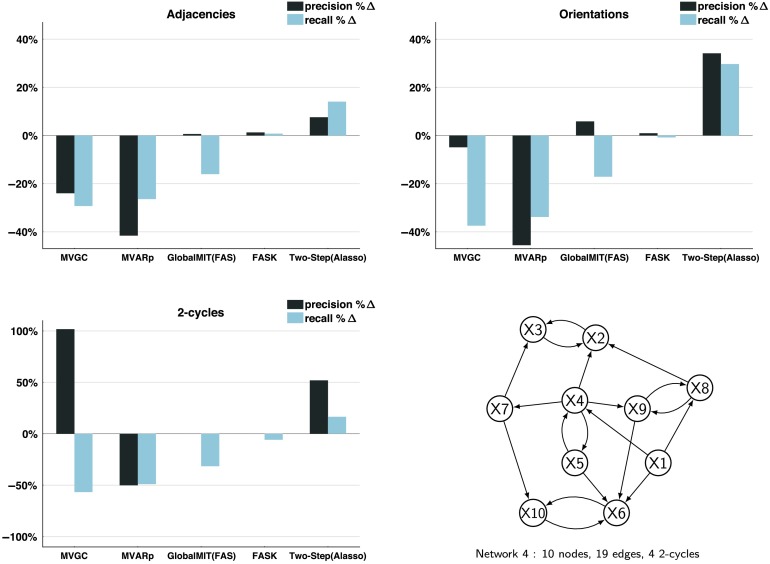
*Effect of removing measurement noise*. Percentage change (%Δ) of precision and recall when the standard Gaussian measurement noise is removed from the data. Results for adjacencies, orientations and 2-cycles, averaged across 60 repetitions of 10 concatenated sessions. Data simulated from *Network 4*.

To have a sense of the effect of measurement noise on the estimation of cyclic structures for Two-Step and FASK, we generated data for the *Network 4* simulation without standard Gaussian measurement noise, and computed the percentage change in performance between the data with measurement noise and the data without. For comparison, lag-based procedures, MVGC, MVARp, and GlobalMIT are also included. Figure 4 shows average *percentage change* of precision and recall for adjacencies, orientations, and 2-cycles detection for 60 repetitions of 10 concatenated datasets when the Gaussian measurement noise is removed. All considered, elimination of measurement noise decreases recall and precision for MVGC and MVARp, helps GlobalMIT (FAS) minimally with precision but not recall, FASK is mostly unaffected, and helps Two-Step (Alasso) considerably across adjacencies, orientations, and 2-cycle detection. The improvements for Two-Step are expected since without measurement noise the data are now from the true model, but the reasons of the changes on accuracy for the other methods, MVGC, MVARp, and GlobalMIT are unclear. Remarkably, FASK is essentially unaffected by measurement noise, at least within the limits we have explored here.

The measurement noise sensitivity we have explored here corresponds to added noise to the BOLD signal (Equation 3). A related question is the effect on the accuracy of FASK and Two-Step from noise directly added to the neuronal variables *z* (Equation 1). In the DCM model used for our simulations the noise can be modeled as *dz*/*dt* = *σ***A***z* + **C***u* + *n*, where *n* represents the added noise. Theoretically, this noise will be transferred to the BOLD signal through the hemodynamic function, increasing the variance of the observed BOLD and mainly hampering the recall (sensitivity) of FASK and Two-Step for the recovery of true adjacencies. The degree to which the recall is affected will depend on the variance of the added noise in the neuronal variables *z*.

#### Temporal undersampling sensitivity.

**Figure 5.**
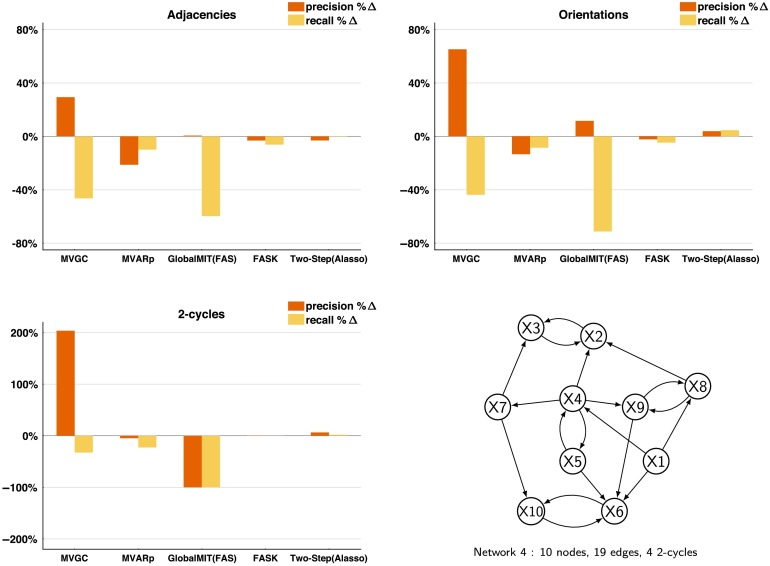
*Effect of reducing the temporal sampling resolution*. Percentage change (%Δ) of precision and recall when the sampling resolution is reduced from one sample every 1.2 s (TR = 1.2 s) to a slower rate of one sample every 3 s (TR = 3 s). Results for adjacencies, orientations, and 2-cycles, averaged across 60 repetitions of ten datasets concatenated. Data simulated from *Network 4*.

As with measurement noise, reductions in the temporal resolution of the observed data can affect the performance of causal inference algorithms, especially for lag-based methods. As the sampling rate of the fMRI data recording gets lower, the recall of lag-based methods is expected to decrease given that the information about variables dependency recovered from previous time steps will not contain relevant causal information when the real causal processes occur at a faster rate than the sampling rate (Seth, Chorley, & Barnett, 2013). In contrast, with data collected at a faster sampling rate the recall of lag-based methods is expected to improve. In fact, when the MVGC algorithm was run on noiseless 5-ms resolution synthetic neuronal data from the DCM simulator, recall and precision for adjacencies, orientations and 2-cycles detection were perfect. Following the measurement noise analysis, synthetic BOLD data was produced from *Network 4* at a lower sampling rate of TR = 3 s (datapoints sampled every 3 s), in order to compare it with the original data with a higher sampling rate of TR = 1.2 s. For the 3-s TR simulated data, the scanning session was increased to 25 min to guarantee a sample size of 500 datapoints in each individual dataset, as in the original data. Figure 5 shows average *percentage change* of precision and recall for adjacencies, orientations and 2-cycle detection for 60 repetitions of 10 concatenated datasets when the sampling resolution was reduced from 1.2-s TR (higher sampling resolution) to 3-s TR (lower sampling resolution), for MVGC, MVARp, GlobalMIT (FAS), FASK, and Two-Step (Alasso). The reduction in sampling rate reduced the adjacencies, orientations, and 2-cycle detection recall for the lag-based methods MVGC and GlobalMIT (FAS) by more than 40%, while MVARp recall and precision was considerably less affected. In contrast, for FASK and Two-Step, which assume i.i.d. data, the reduction in sampling rate did not significantly affect their precision or recall.

## EMPIRICAL DATA RESULTS

### Resting-State Data Results

FASK and Two-Step (Alasso) were run on 23 repetitions of 10 individual subjects selected at random, standardized individually, and concatenated. The resulting 23 concatenated datasets comprise seven regions of interest from the medial temporal lobe, with 4,210 datapoints. Left and right hemispheres were analyzed separately.

In contrast to simulated data, with empirical data we do not have a fully defined ground truth to exactly assess the performance of causal search algorithms. Instead, we have partial knowledge about the presence of structural connections between brain regions derived experimentally in animal models (Naber, Lopes da Silva, & Witter, 2001; und Halbach & Albrecht, 2002), postmortem in humans (Molnár, Blakey, Bystron, & Carney, 2006; Mori et al., 2017; Mufson, Brady, & Kordower, 1990), and in vivo in humans by using diffusion imaging–based tractography (Zeineh et al., 2017), which nevertheless has a systematic problem of false positive inferred–structural connections (Maier-Hein et al., 2017). In some cases we can also obtain knowledge about the direction of the structural connections by tracer injection experiments in animal models (Markov et al., 2012; Witter & Amaral, 1991). This partial information about the connectivity can be used to evaluate to a certain degree the performance of search algorithms, for example, by examining the presence or absence of directed edges relative to previously established reports. In addition, we can also register robust edges that are consistently estimated under different instantiations of the data. We evaluate the performance of FASK and Two-Step (Alasso) on the medial temporal lobe data by comparing robustly estimated edges for each of the algorithms.

Graphical results for the right hemisphere medial temporal lobe are in Supporting Information C (Section C4.2, Sanchez-Romero et al., 2019). We also include for each algorithm, each hemisphere, and for the concatenated and individual cases the list of directed edges and their frequency of appearance across the 23 instantiations, and the list of 2-cycles ordered by frequency of appearance (Supporting Information C, Section C4, Sanchez-Romero et al., 2019). Agreement with the partial knowledge is maximized when we considered edges that appear in 48% of the 23 repetitions. We define a robust edge as an edge estimated in 48% or more of the 23 repetitions of 10 concatenated subjects, or in the case of the individual subject analysis, in 48% or more of the 23 individual subjects.

**Figure 6.**
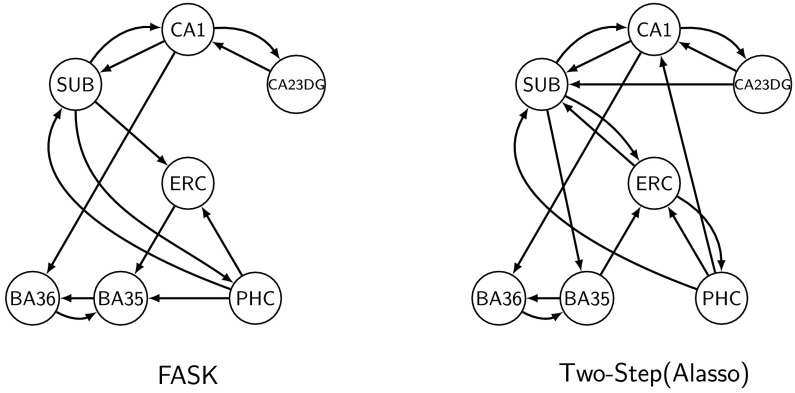
Comparison of the most robust edges estimated by FASK and Two-Step (Alasso) in 23 repetitions of 10 subjects concatenated for 10 regions of interest from the left hemisphere medial temporal lobe. A directed edge is depicted if it is estimated in 48% or more of the 23 repetitions of 10 subjects concatenated. Regions of interest include cornu ammonis 1 (CA1); CA2, CA3, and dentate gyrus together (CA23DG); entorhinal cortex (ERC); perirhinal cortex divided in Brodmann areas (BA35 and BA36); and parahippocampal cortex (PHC).

Figure 6 presents graphically, for the seven regions of interest from the left hemisphere medial temporal lobe, the directed edges estimated in 48% or more of the 23 repetitions of 10 concatenated subjects. We show the most robust edges produced by FASK parameterized with a penalty discount of *c* = 1 and threshold for 2-cycle detection of *α* = 0.05, and compare these edges with the most robust edges estimated by Two-Step (Alasso) parameterized with *λ* = 2 for the **B** matrix estimation, and a threshold of 0.10 for the absolute values of the final **B** matrix.

Overall both algorithms produce a closely similar set of robust edges for the medial temporal lobe left hemisphere data. With one exception, every directed edge found by FASK is found by Two-Step (Alasso). For matters of graph comparison, 2-cycles count as one single adjacency. Given this, it can be seen that the adjacencies output by both algorithms are almost the same with the exception of BA35–PHC that is found by FASK but not by Two-Step; and SUB–CA23DG, SUB–BA35, and PHC–CA1 that are found by Two-Step but not by FASK. Regarding inferred 2-cycles, both algorithms output the 2-cycles for SUB–CA1, CA1–CA32DG and BA35–BA36; the 2-cycle for SUB–PHC is present in the FASK output but not in Two-Step, while the SUB–ERC and ERC–PHC 2-cycles are present in Two-Step but not in FASK. The edge BA35–ERC is oriented in opposite directions by the two algorithms. The adjacencies and orientations are consistent with the medial temporal lobe model presented in Lavenex and Amaral (2000), capturing the flow of information from the medial temporal lobe cortices (BA35, BA36, PHC) directly into the entorhinal cortex (ERC), which works as a gateway to the hippocampal formation, where the signals travel to CA23DG to CA1 (Schaffer collaterals) to the subiculum (SUB) and back to ERC, and from ERC back to BA35, BA36, and PHC. As suggested by Lavenex and Amaral (2000) there are also direct two-way connections between PHC, BA35, and BA36 (perirhinal cortex) and subiculum and CA1, captured with some degree of agreement by FASK and Two-Step. The presence of 2-cycles in the output of both algorithms is consistent with reported feedback in medial temporal lobe information processing (Insausti et al., 2017). There are two main discrepancies of our results with the standard model of the hippocampus as presented in Lavenex and Amaral (2000). First, neither FASK nor Two-Step robustly inferred the ERC–CA23DG edge (perforant pathway). This is surprising since this is the main pathway connecting the medial temporal lobe cortices with the hippocampus. One possible explanation is that the signal between these regions measured with resting-state fMRI is not strong enough to be captured by our methods. Another explanation is that the efficacy of existing structural connectivity is modulated in a task-dependent fashion, so not all possible pathways should be functionally activated in resting state. One other possibility is that the regions of interest for ERC, or CA23DG or both were incorrectly spatially defined and necessary voxels driving the associations between the regions were left out. The second discrepancy is related to the CA23DG–SUB edge which shows in the Two-Step output but not in the FASK result. Again, a possible explanation of this spurious edge is related to a weak mixing of variables between CA1 and CA23DG that affects the more sensitive Alasso adjacency search in Two-Step, but not FASK which is less sensitive to weak signals. Equivalent results are observed for the right hemisphere medial temporal lobe, with the difference that the right hemisphere data show more robust 2-cycles but loses some of the direct connections from the parahippocampal and perirhinal cortices to the hippocampus. Results for the right hemisphere are shown in Supporting Information C (Sanchez-Romero et al., 2019). Surprisingly, however Two-Step with Alasso finds the ERC–CA23DG connection in 57% of the individual datasets (Table C35 in Supporting Information C, Sanchez-Romero et al., 2019).

The analysis using individual datasets showed similar results as the concatenated datasets, with the difference that the frequencies of estimation of the edges across the 23 individuals is lower than with the concatenated datasets. This is expected since reductions of the sample size reduces the ability to detect real effects.

### Task Data Results

**Figure 7.**
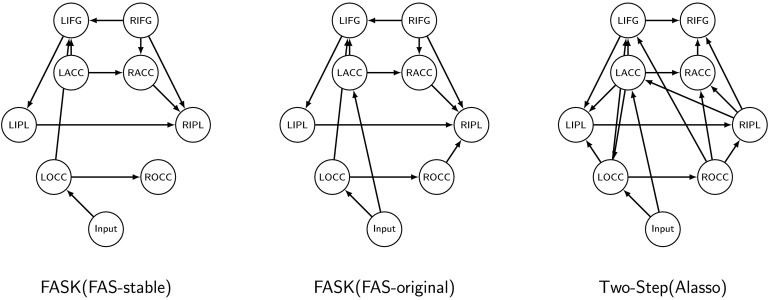
Comparison of the output graphs from FASK with FAS-stable and FAS-original, and Two-Step (Alasso) in one repetition of nine subjects concatenated (1,440 datapoints), for eight bilateral regions of interest and one Input variable. Regions of interest include left and right occipital cortex (LOCC, ROCC); left and right anterior cingulate cortex (LACC, RACC); and left and right inferior frontal gyrus (LIFG, RIFG); and left and right inferior parietal (LIPL, RIPL). The output of FASK (FAS-original) is identical to FASK (FAS-stable) with the addition of the Input → LACC and ROCC → RIPL connections.

FASK and Two-Step (Alasso) were run on one repetition of 9 subjects concatenated (1,440 datapoints) from the rhyming task data, for eight bilateral regions of interest and one Input variable representing the dynamics of the stimuli presentation. FASK was run separately using FAS-stable and FAS-original (the order dependent adjacency search of the PC algorithm; Spirtes et al., 2000) using the same penalty for the BIC* score of *c* = 1 and *α* = 0.001 for the 2-cycle detection. Two-Step (Alasso) was run with a *λ* = 2 for the penalization and a threshold of the absolute values of the **B** matrix of 0.10. The resulting graph for each algorithm is shown in Figure 7.

As mentioned in the Empirical Data section, when analyzing task data we can model the dynamics of the task with an Input variable for which we expect feedforward edges into the regions of interest and not vice versa. This is a simple limited but gold standard test for the accuracy of orientation algorithms. Both, FASK and Two-Step (Alasso) with concatenated data, correctly output feedforward edges from the Input variable to the regions of interest. Of particular relevance is the edge going from the Input to the left occipital cortex, which we expect as this is a task in which visual stimuli are presented first. Both algorithms present feedforward edges from the left occipital cortex to the frontal lobe (LIFG, left inferior frontal gyrus), and then back to the parietal lobe (LIPL, left inferior parietal). This causal order is consistent with the rhyming task (Ramsey et al., 2010), in which first, a pair of words (or pseudowords) are presented in a screen, and then the subjects have to conclude if the words rhyme or do not rhyme. In addition to the posterior anterior direction, we also see in the graphs a cascade of homotopic effects from the left hemisphere to the right hemisphere. The graph produced by the Two-Step algorithm is more dense, which is expected given Alasso higher sensitivity to detect adjacencies under reduced sample sizes compared with FASK. Nevertheless, the adjacencies of FASK(FAS-stable) and FASK(FAS-original) are proper subsets of the Two-Step adjacencies.

There are some differences in orientations between the algorithms in the right hemisphere, but the left hemisphere orientations are very consistent, especially regarding the flow from occipital to frontal to parietal. Interestingly, neither FASK nor Two-Step produce 2-cycles between the regions of interest. If effects are weak, FASK may miss the presence of a 2-cycle, but Two-Step (Alasso) will not, which give us some guarantee that the absence of 2-cycles for these regions under this particular task is a real connectivity property and not a problem of false negatives. These task data have been previously analyzed with algorithms that assume acyclicity (Ramsey et al., 2010), and pairwise non-Gaussian orientation algorithms (Ramsey et al., 2014), with similar results in terms of the gross flow of information from occipital to frontal regions and from left to right hemisphere.

For comparison, in Supporting Information C (Section C5, Sanchez-Romero et al., 2019) we present results for FASK(FAS-stable) and Two-Step (Alasso) on each of the nine individual datasets (160 datapoints). For each algorithm we report frequency of estimation for each directed edge, including 2-cycles. Even with individual data (160 datapoints) we can recover robust edges that are consistent with those obtained when we concatenated the nine subjects (Figure 7), especially those regarding homotopic connectivity from left to right hemisphere. We also observed that for FASK the directed edge from the Input variable to the left occipital region (LOCC) is hardly obtained at the individual level, whereas Two-Step (Alasso) has a better performance producing that edge in seven out of nine individual subjects. Two-Step seems prone to false positive edges when the sample size is small as in the individual scans.

## DISCUSSION

At present temporal resolution for fMRI data, lag-based methods are unreliable in three respects. They overfit, their accuracies are sensitive to the undersampling rate, and they do poorly in finding feedback cycles. The two non-Gaussian i.i.d. methods presented here, FASK and Two-Step, are far from perfect but they are more accurate than available lag-based methods. FASK application is very simple with only two adjustable parameters and short running time even in large dimension problems. In contrast, because of the optimization procedure to infer the **B** matrix coefficients, Two-Step can be slow, especially with large dimension problems or when the sample size is very large. This can be mitigated by initializing the optimization of the mixing matrix with small values for the coefficients and adjusting the penalization parameters as we did here. Both FASK and Two-Step are insensitive to variations in undersampling, can infer cycles of any degree, and they are distinctively good at finding 2-cycles. FASK is robust to measurement noise in the observed variables, a relevant property considering that measurement noise has shown to be detrimental to the performance of certain search algorithms including Two-Step. The non-Gaussianity leveraged by FASK to orient edges comes from the skewness of the distribution around the center: if the distribution of the data is non-Gaussian but symmetric around the center, such as the Uniform distribution, FASK is not guaranteed to infer the correct orientations. In contrast, Two-Step works accurately with any type of non-Gaussian distribution, even with non-Gaussian distributions symmetric around the center. It is still controversial whether BOLD signals are significantly non-Gaussian (Hlinka, Paluš, Vejmelka, Mantini, & Corbetta, 2011), so to quantify the presence of skewness (a measure of non-Gaussianity) in our empirical BOLD data we computed the mean skewness across all variables and all individual datasets and compared it against the mean skewness of an equivalent number of values of random variables sampled from a Gaussian distribution. We did this for the resting-state medial temporal lobe data and for the task data separately. In both cases, the difference between the mean skewness of the empirical BOLD data and the mean skewness of the random Gaussian variables was statistically significant at an *α* = 0.01, using a two-sided *z*-test for the difference of means. From this we can conclude that the empirical BOLD data used here present statistically significant non-Gaussianity (coming from the skewness) that was exploited by FASK and Two-Step.

Almost all of the hitherto published tests of discovery methods for effective connections using simulated fMRI data have used very sparse graphs, typically of degree 2 or 3, to generate data. Increased density of causal connections will inevitably result in lower recall from search methods—a larger fraction of the true connections will not be discovered. The precision—the fraction of connections reported by a search method that are correct—will be affected as well, although less severely. Here, we have analyzed a much denser simulation based on empirical anatomical connections in the macaque network, with average degree 35, representing 34% of all possible adjacencies among the 91 nodes in the network (or 19.7% of all possible directed edges, counting 2-cycles as two distinct directed edges). For this network, Two-Step has substantial recall and good precision, but FASK, although with perfect precision, is essentially uninformative, returning only 17 adjacencies. The disappointing recall for FASK is very likely the result of two factors. One is the possibly quite unrealistic very low coefficient values that were necessary to find in our simulator a stable model of the highly cyclic macaque network. The other is the adjacency search used in FASK—the fast adjacency search (FAS) of the PC algorithm. The quasi-Bayesian Fast Greedy Equivalence Search (FGES) algorithm (Ramsey et al., 2017) has an adjustable penalty that can be increased or decreased to force sparser or denser estimated graphs when the true graph is dense. Run on all cortical voxels for empirical resting-state fMRI data, Ramsey et al. (2017) found that more than 90% of the edges found with sparser penalties were included in the edges found with denser penalties, suggesting that for the adjacency search in FASK, one might substitute FGES. FGES is available in the TETRAD software (http://www.phil.cmu/tetrad).

The Two-Step algorithm outputs estimates of connection strengths (weighted directed matrix), which may be relevant for some applications, for example, connectivity-based classification. FASK does not give estimates of coefficients: its output is a possibly cyclic graph that can be encoded as a binary directed matrix. Notwithstanding, the graph output by FASK can be parameterized as a structural equation model and coefficients estimated from the data. We implemented FASK in Java, but the 2-cycle detection and left-right rules are flexible enough to be implemented in other programming languages such as R, Python, or Matlab. Two-Step was implemented in Matlab but it can also be implemented in R, Python, or Java as long as the optimization method used has efficient running times.

In theory, Two-Step accurately finds causal connections among measured variables in the presence of unrecorded, unknown confounding variables, but we have not tested that aspect of the procedure here. None of the other procedures tested here have any theoretical guarantees when there are unrecorded confounders. Researchers need a reasonably accurate procedure that can be run on such high-dimensional problems and is robust both to feedback cycles and to unmeasured confounders.

All of the search algorithms tested in our manuscript require setting one or more tuning parameters that control the sparsity of the estimated network. In novel empirical applications, we recommend generating simulated data that accords with the empirical sample size, number of variables, correlations, and any prior information the investigator has about structure, and choose tuning parameters that when applied in search yield the best agreement with the generating structure(s) of the simulations. That is essentially what we have done in applying our methods to the empirical resting-state and task data described here.

For our application of FASK and Two-Step to empirical data we used tuning parameters values from the optimal space of tuning parameters obtained in simulations for the macaque *SmallDegree* network (Figures B1 and B2 from Supporting Information B, Sanchez-Romero et al., 2019), which we consider holds similar characteristics to our empirical data in terms of sample size, sparsity, and coefficient strength (which can be loosely approximated with correlation strengths). We observe that the results of FASK and Two-Step with the chosen parameters produce results in agreement with reported knowledge, and with the ground truth that in task data, the input variable representing the task time series should feed into the brain nodes and not vice versa. We recommend a comparable check on any application of structural search methods to task related data.

In Table 2 we showed the positive effect on recall of increasing the sample size for the *LongRange* macaque simulation. A related question is, which is the minimum sample size required for FASK and Two-Step to obtain reliable precision *and* recall. To answer this question, we ran FASK and Two-Step (Alasso) under increasing sample size (obtained by concatenating datasets) for the *Network 4* simulation. The minimum sample size tested was 500 and the maximum 30,000. For this structure, FASK maximizes its recall from 5,000 datapoints (i.e., 10 datasets concatenated) onward, while high precision is achieved for all the sample sizes we tested. For 2-cycles detection, perfect accuracy is reached by FASK starting with 2,000 datapoints (i.e., 4 datasets concatenated). In this set up, Two-Step seems to maximize its precision from 7,500 datapoints (i.e., 15 datasets concatenated) onward but with reduction in recall. Recall of 2-cycles detection improves also around 7,500 datapoints, but accompanied by a reduction in the precision. Overall these results suggest that good precision *and* recall for FASK can be achieved with approximately 5,000 datapoints; there is a trade-off between higher and lower precision for Two-Step, and between overall edge orientation precision and 2-cycle detection precision. We include tables with full results in Supporting Information E (Sanchez-Romero et al., 2019).

The sample sizes suggested above guarantees, in simulation, a high precision and recall for FASK and Two-Step. Nevertheless, data with such large sample sizes may not be available or it may not be desirable to concatenate datasets to increase the sample size. For such scenarios, we ask if FASK and Two-Step can achieve *precision* as good as that achieved with larger sample size with a tolerable loss in recall. Bühlmann and van de Geer (2011, Chapter 10) propose a strategy for small sample regimes which potentially reduces the number of false positive edges and thus boosts the precision of a search method. This strategy has been previously used with Lasso and Glasso applications (Bühlmann & van de Geer, 2011), and we apply it here to FASK and Two-Step. The strategy consists in creating *k* random subsamples of size *N*/2 without replacement (within each subsample) from an original dataset of sample size *N*; infer *k* graphs and count the number of times a particular edge appears across the *k* graphs. A final graph is built with those edges that appear in more than a proportion *t* of the *k* graphs, where 0.50 < *t* ≤ 1. The value of *t* is a threshold that depends on the number of false positive edges tolerated by the researcher. The smaller the number of false positives tolerated the higher the value of *t* that should be set. Using datasets with 500 datapoints, we test this subsampling strategy for FASK and Two-Step (Alasso) on the 18 simple simulations and in the four macaque-based simulations. We compare precision and recall for these datasets against those obtained with the concatenated datasets of 5,000 datapoints for which results were reported in Figures 2 and 3 and Table 1. For the subsampling strategy we use *k* = 100 to guarantee stability of the results. The value of *t* may be different for each network and dataset, and depends on the number of tolerated false positives. In our simulations, for smaller size problems, such as the 18 simple simulations, setting the number of tolerated false positives equal to 5% of *p*, the total possible directed edges of the network (*p* = *v*(*v* − 1), where *v* is the number of nodes in the network), produced goods results, but with higher size problems, such as the four macaque-based simulations, we obtained better results with a lower proportion of 1/*p*, as suggested in Bühlmann and van de Geer (2011).

Using FASK with the subsampling strategy and 500 datapoints, the precision for adjacencies and orientations improved—in average across the 18 simple simulations—by 10% and 7%, respectively, relative to using datasets with 5,000 datapoints. The recall dropped by 35% for adjacencies and by 37% for orientations. A reduction in recall is expected since the power of FASK decreased because of the reduction in sample size. Nevertheless, the subsampling strategy helped to achieve a precision better than with 5,000 datapoints. For Two-Step (Alasso) with the subsampling strategy and 500 datapoints, the precision for adjacencies and orientations improved slightly—in average across the 18 simple simulations—by 2% and 1%, respectively; the recall dropped by 15% for adjacencies and 17% for orientations. As with FASK, the reduction in sample size diminished the power of Two-Step and consequently reduced the recall, but precision is as good as with 5,000 datapoints.

For the *Macaque SmallDegree* simulation, both algorithms experienced a reduction in recall due to the reduction in the sample size, but the precision was improved thanks to the subsampling strategy. In contrast, for *Macaque LongRange*, *LongRangeControl*, and *Full* simulations, the reduction in sample size considerably affected both the recall and the precision. The reason is that for these three simulations the coefficients are very small, (0.05–0.1) and (0.01–0.05) respectively, and the reduction in sample size from 5,000 to 500 makes it extremely difficult to detect these minuscule effects, while it also increases the risk of false positives. As a reference, all the other simulations have larger coefficients in the range (0.3–0.7).

All together, these results show that when complemented with the subsampling strategy, FASK and Two-Step with 500 datapoints can achieve precisions as good as those obtained with 5,000 datapoints. The recalls will be smaller compared with those obtained with a large sample size of 5,000, but researchers may have comparable confidence about the inferred edges. When effects are very small, subsampling will not find them. Details about the specific implementation of the subsampling strategy and results for each individual simulation with 500 and 5,000 datapoints are included in Supporting Information F (Sanchez-Romero et al., 2019).

Finally, a number of clinical studies have used hypothetical neuronal connections inferred from correlations (Abraham et al., 2017; Chen, Uddin, Zhang, Duan, & Chen, 2016; Cheng et al., 2017; Di Martino et al., 2014; Kassraian-Fard, Matthis, Balsters, Maathuis, & Wenderoth, 2016; Long, Duan, Mantini, & Chen, 2016; Ray et al., 2014; Uddin, Supekar, & Menon, 2013). In principle, it is possible to achieve good diagnostics from connectivities assumptions that are a mixture of true connections and false positive connections, but more accurate estimation of effective connections is likely to improve accuracy. Price et al. (2017), using the GIMME method we have examined in this paper, analyzed connectivity differences between patients with depression and controls. Although we do not provide any clinical application in this paper, Huang et al. (2017) using Two-Step obtained between 82% and 87% classification accuracy on autism spectrum disorder (ASD) identification (depending on the laboratory source from the ABIDE data repository)—so far as we know the best accuracy yet obtained from resting-state fMRI data.

## ACKNOWLEDGMENTS

We thank Matthieu Gilson for sharing code for the MVAR permutation method, Stephen M. Smith for sharing code for the DCM simulator, and Stephen J. Hanson for comments.

## AUTHOR CONTRIBUTIONS

Sanchez-Romero: Data curation; Formal analysis; Investigation; Methodology; Software; Validation; Visualization; Writing – original draft; Writing – review & editing. Joseph D. Ramsey: Conceptualization; Formal analysis; Investigation; Methodology; Software; Validation; Writing – review & editing. Kun Zhang: Formal analysis; Investigation; Methodology; Software; Validation; Funding acquisition; Writing – review & editing. Madelyn R. K. Glymour: Data curation; Investigation; Software; Validation. Biwei Huang: Formal analysis; Methodology; Software; Validation. Clark Glymour: Conceptualization; Formal analysis; Funding acquisition; Investigation; Methodology; Project administration; Supervision; Writing – original draft; Writing – review & editing.

## FUNDING INFORMATION

Clark Glymour, National Institutes of Health (http://dx.doi.org/10.13039/100000002), Award ID: U54HG008540. Clark Glymour, National Institutes of Health (http://dx.doi.org/10.13039/100000002), Award ID: 5RO1LM012087. Clark Glymour, National Institutes of Health (http://dx.doi.org/10.13039/100000002), Award ID: 1RO1EB022858-01. Kun Zhang, United States Air Force under Contract No. FA8650-17-C-7715.

## TECHNICAL TERMS

Feedback structure: In a directed graph, a set of nodes and edges, in which some nodes are reachable from themselves.
Pairwise directionality algorithms: In causal search, methods that infer the causal direction between each pair of adjacent variables individually.
Measurement noise: Error in observed signal introduced by the activity itself of measuring.
Retrograde tracer injection: An invasive neuronal imaging method where a substance, such as a virus, is injected in a target area to trace back axonal connections to their sources.
Skewness: The third moment of a probability distribution; it measures the asymmetry around its mean. Gaussian distributions have a skewness equal to zero.
Order-independent search: In causal inference, a search algorithm whose output graph does not depend on the order in which statistical tests are performed.
Non-recursive structural equations: A set of equations modeling the causal relations for a feedback model.
Unmeasured confounding variable: A variable that is a common cause of two or more observed variables but is not included in the observed dataset.
Independent component analysis (ICA): A mathematical method for decomposing an observed dataset into a combination of independent, non-Gaussian components.
Regularized regression: A method of regression in which a penalty term is used to avoid overfitting; generally it works by shrinking the values of small estimated regression coefficients to zero.
Structural connectivity: Interactions between neuronal regions captured by axonal connections.

## References

[bib1] Abraham A., Milham M. P., Di Martino A., Craddock R. C., Samaras D., Thirion B., & Varoquaux G. (2017). Deriving reproducible biomarkers from multi-site resting-state data: An autism-based example. NeuroImage, 147, 736–745. 27865923 10.1016/j.neuroimage.2016.10.045

[bib2] Barnett L., & Seth A. K. (2014). The MVGC multivariate Granger causality toolbox: A new approach to Granger-causal inference. Journal of Neuroscience Methods, 223, 50–68. 24200508 10.1016/j.jneumeth.2013.10.018

[bib3] Bellucci G., Chernyak S., Hoffman M., Deshpande G., Dal Monte O., Knutson K. M., … Krueger F. (2017). Effective connectivity of brain regions underlying third-party punishment: Functional MRI and Granger causality evidence. Social Neuroscience, 12(2), 124–134. 26942651 10.1080/17470919.2016.1153518

[bib4] Benjamini Y., & Hochberg Y. (1995). Controlling the false discovery rate: A practical and powerful approach to multiple testing. Journal of the Royal Statistical Society. Series B (Methodological), 289–300.

[bib5] Bühlmann P., & van de Geer S. (2011). Statistics for High-Dimensional Data: Methods, Theory and Applications. Berlin: Springer Science & Business Media.

[bib6] Chen H., Uddin L. Q., Zhang Y., Duan X., & Chen H. (2016). Atypical effective connectivity of thalamo-cortical circuits in autism spectrum disorder. Autism Research, 9(11), 1183–1190. 27868393 10.1002/aur.1614

[bib7] Cheng W., Rolls E. T., Zhang J., Sheng W., Ma L., Wan L., … Feng J. (2017). Functional connectivity decreases in autism in emotion, self, and face circuits identified by knowledge-based enrichment analysis. NeuroImage, 148, 169–178. 28040544 10.1016/j.neuroimage.2016.12.068

[bib8] Colombo D., & Maathuis M. H. (2014). Order-independent constraint-based causal structure learning. The Journal of Machine Learning Research, 15(1), 3741–3782.

[bib9] Di Martino A., Fair D. A., Kelly C., Satterthwaite T. D., Castellanos F. X., Thomason M. E., … Milham M. P. (2014). Unraveling the miswired connectome: A developmental perspective. Neuron, 83(6), 1335–1353. 25233316 10.1016/j.neuron.2014.08.050 PMC4169187

[bib10] Dubois J., Oya H., Tyszka J. M., Howard M., Eberhardt F., & Adolphs R. (in press). Causal mapping of emotion networks in the human brain: Framework and initial findings. Neuropsychologia. 10.1016/j.neuropsychologia.2017.11.015 PMC5949245 29146466

[bib11] Ekstrom A. D., Bazih A. J., Suthana N. A., Al-Hakim R., Ogura K., Zeineh M., … Bookheimer S. Y. (2009). Advances in high-resolution imaging and computational unfolding of the human hippocampus. NeuroImage, 47(1), 42–49. 19303448 10.1016/j.neuroimage.2009.03.017 PMC2689320

[bib12] Freenor M., & Glymour C. (2010). Searching the DCM model space, and some generalizations. Carnegie Mellon University, Department of Philosophy Technical Report No. CMU-PHIL-185 Retrieved from https://www.cmu.edu/dietrich/philosophy/docs/tech-reports/185_Freenor.pdf

[bib13] Friedman J., Hastie T., & Tibshirani R. (2008). Sparse inverse covariance estimation with the graphical lasso. Biostatistics, 9(3), 432–441. 18079126 10.1093/biostatistics/kxm045 PMC3019769

[bib14] Friston K. J., Harrison L., & Penny W. (2003). Dynamic causal modelling. NeuroImage, 19(4), 1273–1302. 12948688 10.1016/s1053-8119(03)00202-7

[bib15] Friston K. J., Li B., Daunizeau J., & Stephan K. E. (2011). Network discovery with DCM. NeuroImage, 56(3), 1202–1221. 21182971 10.1016/j.neuroimage.2010.12.039 PMC3094760

[bib16] Gates K. M., & Molenaar P. C. M. (2012). Group search algorithm recovers effective connectivity maps for individuals in homogeneous and heterogeneous samples. NeuroImage, 63(1), 310–319. 22732562 10.1016/j.neuroimage.2012.06.026

[bib17] Gates K. M., Molenaar P. C. M., Iyer S. P., Nigg J. T., & Fair D. A. (2014). Organizing heterogeneous samples using community detection of GIMME-derived resting state functional networks. PloS One, 9(3), e91322. 24642753 10.1371/journal.pone.0091322 PMC3958357

[bib18] Gilson M., Tauste Campo A., Chen X., Thiele A., & Deco G. (2017). Nonparametric test for connectivity detection in multivariate autoregressive networks and application to multiunit activity data. Network Neuroscience, 1, 357–380. 30090871 10.1162/NETN_a_00019 PMC6063719

[bib19] Goense J. B. M., & Logothetis N. K. (2008). Neurophysiology of the BOLD fMRI signal in awake monkeys. Current Biology, 18(9), 631–640. 18439825 10.1016/j.cub.2008.03.054

[bib20] Granger C. W. J. (1969). Investigating causal relations by econometric models and cross-spectral methods. Econometrica: Journal of the Econometric Society, 37, 424–438.

[bib21] Gruber P., Gutch H. W., & Theis F. J. (2009). Hierarchical extraction of independent subspaces of unknown dimensions. In Adali T., Jutten C., Romano J. M. T., & Barros A. K. (Eds.), Independent Component Analysis and Signal Separation. ICA 2009. Lecture Notes in Computer Science (Vol. 5441, pp. 259–266). Berlin: Springer.

[bib22] He B. J., Zempel J. M., Snyder A. Z., & Raichle M. E. (2010). The temporal structures and functional significance of scale-free brain activity. Neuron, 66(3), 353–369. 20471349 10.1016/j.neuron.2010.04.020 PMC2878725

[bib23] Heise D. R. (1975). Causal Analysis. New York: John Wiley & Sons.

[bib24] Hlinka J., Paluš M., Vejmelka M., Mantini D., & Corbetta M. (2011). Functional connectivity in resting-state fMRI: Is linear correlation sufficient? NeuroImage, 54(3), 2218–2225. 20800096 10.1016/j.neuroimage.2010.08.042 PMC4139498

[bib25] Hoover K. D. (2005). Automatic inference of the contemporaneous causal order of a system of equations. Econometric Theory, 21(1), 69–77.

[bib26] Huang B., Zhang K., Sanchez-Romero R., Ramsey J., Glymour M., & Glymour C. (2017). Diagnosis of autism spectrum disorder with causal connectivity strength discovered from resting-state fMRI. Poster presented at the Society for Neuroscience Meeting 2017, Washington D.C., USA Retrieved from http://bit.ly/HuangAustismSfN2017

[bib27] Hyvärinen A., & Oja E. (2000). Independent component analysis: Algorithms and applications. Neural Networks, 13(4–5), 411–430. 10946390 10.1016/s0893-6080(00)00026-5

[bib28] Hyvärinen A., & Smith S. M. (2013). Pairwise likelihood ratios for estimation of non-Gaussian structural equation models. Journal of Machine Learning Research, 14(Jan), 111–152. PMC6834441 31695580

[bib29] Insausti R., Marcos M., Mohedano-Moriano A., Arroyo-Jiménez M., Córcoles-Parada M., Artacho-Pérula E., … Muñoz-López M. (2017). The nonhuman primate hippocampus: Neuroanatomy and patterns of cortical connectivity. In The Hippocampus from Cells to Systems (pp. 3–36). Cham, Switzerland: Springer.

[bib30] Itani S., Ohannessian M., Sachs K., Nolan G. P., & Dahleh M. A. (2010). Structure learning in causal cyclic networks. In Proceedings of Workshop on Causality: Objectives and Assessment at NIPS 2008, PMLR (Vol. 6, pp. 165–176).

[bib31] Jöreskog K. G., & Sörbom D. (1993). LISREL 8: Structural equation modeling with the SIMPLIS command language. Scientific Software International.

[bib32] Juan-Cruz C., Gómez C., Poza J., Fernández A., & Hornero R. (2017). Assessment of effective connectivity in Alzheimer’s disease using Granger causality. In Converging Clinical and Engineering Research on Neurorehabilitation II (pp. 763–767). Cham, Switzerland: Springer.

[bib33] Kahn I., Desai M., Knoblich U., Bernstein J., Henninger M., Graybiel A. M., … Moore C. I. (2011). Characterization of the functional MRI response temporal linearity via optical control of neocortical pyramidal neurons. Journal of Neuroscience, 31(42), 15086–15091. 22016542 10.1523/JNEUROSCI.0007-11.2011 PMC3225054

[bib34] Kassraian-Fard P., Matthis C., Balsters J. H., Maathuis M. H., & Wenderoth N. (2016). Promises, pitfalls, and basic guidelines for applying machine learning classifiers to psychiatric imaging data, with autism as an example. Frontiers in Psychiatry, 7, 177. 27990125 10.3389/fpsyt.2016.00177 PMC5133050

[bib35] Kötter R., & Stephan K. E. (2003). Network participation indices: Characterizing component roles for information processing in neural networks. Neural Networks, 16(9), 1261–1275. 14622883 10.1016/j.neunet.2003.06.002

[bib36] Lavenex P., & Amaral D. G. (2000). Hippocampal-neocortical interaction: A hierarchy of associativity. Hippocampus, 10(4), 420–430. 10985281 10.1002/1098-1063(2000)10:4<420::AID-HIPO8>3.0.CO;2-5

[bib37] Long Z., Duan X., Mantini D., & Chen H. (2016). Alteration of functional connectivity in autism spectrum disorder: Effect of age and anatomical distance. Scientific Reports, 6, 26527. 27194227 10.1038/srep26527 PMC4872225

[bib38] Maier-Hein K. H., Neher P. F., Houde J.-C., Côté M.-A., Garyfallidis E., Zhong J., … others (2017). The challenge of mapping the human connectome based on diffusion tractography. Nature Communications, 8(1), 1349. 10.1038/s41467-017-01285-x PMC5677006 29116093

[bib39] Markov N. T., Ercsey-Ravasz M., Lamy C., Gomes A. R. R., Magrou L., Misery P., … Kennedy H. (2013). The role of long-range connections on the specificity of the macaque interareal cortical network. Proceedings of the National Academy of Sciences of the United States of America, 110(13), 5187–5192. 23479610 10.1073/pnas.1218972110 PMC3612613

[bib40] Markov N. T., Ercsey-Ravasz M. M., Ribeiro Gomes A. R., Lamy C., Magrou L., Vezoli J., … Kennedy H. (2012). A weighted and directed interareal connectivity matrix for macaque cerebral cortex. Cerebral Cortex, 24(1), 17–36. 23010748 10.1093/cercor/bhs270 PMC3862262

[bib41] Matthews B. W. (1975). Comparison of the predicted and observed secondary structure of t4 phage lysozyme. Biochimica et Biophysica Acta (BBA)-Protein Structure, 405(2), 442–451. 10.1016/0005-2795(75)90109-9 1180967

[bib42] Molnár Z., Blakey D., Bystron I., & Carney R. S. (2006). Tract-tracing in developing systems and in postmortem human material using carbocyanine dyes. Neuroanatomical Tract-Tracing 3, 366–393.

[bib43] Mooij J. M., Peters J., Janzing D., Zscheischler J., & Schölkopf B. (2016). Distinguishing cause from effect using observational data: Methods and benchmarks. The Journal of Machine Learning Research, 17(1), 1103–1204.

[bib44] Mori S., Kageyama Y., Hou Z., Aggarwal M., Patel J., Brown T., … Troncoso J. C. (2017). Elucidation of white matter tracts of the human amygdala by detailed comparison between high-resolution postmortem magnetic resonance imaging and histology. Frontiers in Neuroanatomy, 11. 10.3389/fnana.2017.00016 PMC5348491 28352217

[bib45] Mufson E. J., Brady D. R., & Kordower J. H. (1990). Tracing neuronal connections in postmortem human hippocampal complex with the carbocyanine dye DiI. Neurobiology of Aging, 11(6), 649–653. 1704107 10.1016/0197-4580(90)90031-t

[bib46] Naber P. A., Lopes da Silva F. H., & Witter M. P. (2001). Reciprocal connections between the entorhinal cortex and hippocampal fields CA1 and the subiculum are in register with the projections from CA1 to the subiculum. Hippocampus, 11(2), 99–104. 11345131 10.1002/hipo.1028

[bib47] P. Santos F., Smagula S. F., Karim H., Santini T. S., Aizenstein H., Ibrahim T. S., & Maciel C. D. (2017). Dynamic Bayesian network modeling of hippocampal subfields connectivity with 7T fMRI: A case study. Proceedings of the 10th International Joint Conference on Biomedical Engineering Systems and Technologies (January), 178–184.

[bib48] Patel R. S., Bowman F., & Rilling J. K. (2006). A Bayesian approach to determining connectivity of the human brain. Human Brain Mapping, 27(3), 267–276. 16092131 10.1002/hbm.20182 PMC6871439

[bib49] Pearson K., Lee A., & Bramley-Moore L. (1899). Mathematical contributions to the theory of evolution. VI. Genetic (reproductive) selection: Inheritance of fertility in man, and of fecundity in thoroughbred racehorses. Philosophical Transactions of the Royal Society of London. Series A, Containing Papers of a Mathematical or Physical Character, 192, 257–330.

[bib50] Poldrack R. A., Laumann T. O., Koyejo O., Gregory B., Hover A., Chen M.-Y., … Mumford J. A. (2015). Long-term neural and physiological phenotyping of a single human. Nature Communications, 6, 8885. 10.1038/ncomms9885 PMC4682164 26648521

[bib51] Preston A. R., Bornstein A. M., Hutchinson J. B., Gaare M. E., Glover G. H., & Wagner A. D. (2010). High-resolution fMRI of content-sensitive subsequent memory responses in human medial temporal lobe. Journal of Cognitive Neuroscience, 22(1), 156–173. 19199423 10.1162/jocn.2009.21195 PMC2854293

[bib52] Price R. B., Lane S., Gates K., Kraynak T. E., Horner M. S., Thase M. E., & Siegle G. J. (2017). Parsing heterogeneity in the brain connectivity of depressed and healthy adults during positive mood. Biological Psychiatry, 81(4), 347–357. 27712830 10.1016/j.biopsych.2016.06.023 PMC5215983

[bib53] Ramsey J., Glymour M., Sanchez-Romero R., & Glymour C. (2017). A million variables and more: The Fast Greedy Equivalence Search algorithm for learning high-dimensional graphical causal models, with an application to functional magnetic resonance images. International Journal of Data Science and Analytics, 3(2), 121–129. 28393106 10.1007/s41060-016-0032-z PMC5380925

[bib54] Ramsey J., Hanson S. J., Hanson C., Halchenko Y. O., Poldrack R. A., & Glymour C. (2010). Six problems for causal inference from fMRI. NeuroImage, 49(2), 1545–1558. 19747552 10.1016/j.neuroimage.2009.08.065

[bib55] Ramsey J., Sanchez-Romero R., & Glymour C. (2014). Non-Gaussian methods and high-pass filters in the estimation of effective connections. NeuroImage, 84, 986–1006. 24099845 10.1016/j.neuroimage.2013.09.062

[bib56] Ramsey J., Spirtes P., & Glymour C. (2011). On meta-analyses of imaging data and the mixture of records. NeuroImage, 57(2), 323–330. 20709178 10.1016/j.neuroimage.2010.07.065

[bib57] Ray S., Miller M., Karalunas S., Robertson C., Grayson D. S., Cary R. P., … Fair D. A. (2014). Structural and functional connectivity of the human brain in autism spectrum disorders and attention-deficit/hyperactivity disorder: A rich club-organization study. Human Brain Mapping, 35(12), 6032–6048. 25116862 10.1002/hbm.22603 PMC4319550

[bib58] Richardson T. (1996). A discovery algorithm for directed cyclic graphs. In Proceedings of the 12th Conference on Uncertainty in Artificial Intelligence, Portland, Oregon, 1996. Horvitz E. and Jensen F. (Eds.), Morgan Kaufmann, San Francisco, CA (Vol. 53, pp. 1689–1699).

[bib59] Sanchez-Romero R. (2012). Formation of variables for brain connectivity (Master’s thesis, Carnegie Mellon University) Retrieved from http://www.andrew.cmu.edu/user/rubens/research/MSThesisRubenSanchez.pdf

[bib60] Sanchez-Romero R., Ramsey J. D., Zhang K., Glymour M. R. K., Huang B., & Glymour C. (2019). Supporting information for “Estimating feedforward and feedback effective connections from fMRI time series: Assessments of statistical methods.” Network Neuroscience, 3(2), 274–306. 10.1162/netn_a_00061 PMC6370458 30793083

[bib61] Scheines R., & Ramsey J. (2016). Measurement error and causal discovery. In CEUR Workshop Proceedings (Vol. 1792, p. 1). PMC5340263 28280453

[bib62] Schwarz G., (1978). Estimating the dimension of a model. The Annals of Statistics, 6(2), 461–464.

[bib63] Seth A. K., Chorley P., & Barnett L. C. (2013). Granger causality analysis of fMRI BOLD signals is invariant to hemodynamic convolution but not downsampling. NeuroImage, 65, 540–555. 23036449 10.1016/j.neuroimage.2012.09.049

[bib64] Shah P., Bassett D. S., Wisse L. E., Detre J. A., Stein J. M., Yushkevich P. A., … Das S. R. (2017). Mapping the structural and functional network architecture of the medial temporal lobe using 7T MRI. Human Brain Mapping, 39, 851–865. 29159960 10.1002/hbm.23887 PMC5764800

[bib65] Shimizu S., Hoyer P. O., Hyvärinen A., & Kerminen A. (2006). A linear non-Gaussian acyclic model for causal discovery. Journal of Machine Learning Research, 7(Oct), 2003–2030.

[bib66] Smith S. M., Miller K. L., Salimi-Khorshidi G., Webster M., Beckmann C. F., Nichols T. E., … Woolrich M. W. (2011). Network modelling methods for fMRI. NeuroImage, 54(2), 875–891. 20817103 10.1016/j.neuroimage.2010.08.063

[bib67] Spirtes P., Glymour C. N., & Scheines R. (2000). Causation, Prediction, and Search. Cambridge, MA: MIT Press.

[bib68] Sreenivasan K., Zhuang X., Banks S. J., Mishra V., Yang Z., Deshpande G., & Cordes D. (2017). Olfactory network differences in master sommeliers: Connectivity analysis using Granger causality and graph theoretical approach. Brain Connectivity, 7(2), 123–136. 28125912 10.1089/brain.2016.0458 PMC5359656

[bib69] Swanson N. R., & Granger C. W. J. (1997). Impulse response functions based on a causal approach to residual orthogonalization in vector autoregressions. Journal of the American Statistical Association, 92(437), 357–367.

[bib70] Theis F. J. (2007). Towards a general independent subspace analysis. NIPS 2006 (pp. 1361–1368).

[bib71] Tibshirani R. (1996). Regression shrinkage and selection via the lasso. Journal of the Royal Statistical Society. Series B (Methodological), 267–288.

[bib72] Uddin L. Q., Supekar K., & Menon V. (2013). Reconceptualizing functional brain connectivity in autism from a developmental perspective. Frontiers in Human Neuroscience, 7, 458. 23966925 10.3389/fnhum.2013.00458 PMC3735986

[bib73] und Halbach O. v. B., & Albrecht D. (2002). Reciprocal connections of the hippocampal area CA1, the lateral nucleus of the amygdala and cortical areas in a combined horizontal slice preparation. Neuroscience Research, 44(1), 91–100. 12204297 10.1016/s0168-0102(02)00092-5

[bib74] Vinh N. X., Chetty M., Coppel R., & Wangikar P. P. (2011). Globalmit: Learning globally optimal dynamic bayesian network with the mutual information test criterion. Bioinformatics, 27(19), 2765–2766. 21813478 10.1093/bioinformatics/btr457

[bib75] Winder A. T., Echagarruga C., Zhang Q., & Drew P. J. (2017). Weak correlations between hemodynamic signals and ongoing neural activity during the resting state. Nature Neuroscience, 20, 1761–1769. 29184204 10.1038/s41593-017-0007-y PMC5816345

[bib76] Witter M. P., & Amaral D. G. (1991). Entorhinal cortex of the monkey: V. Projections to the dentate gyrus, hippocampus, and subicular complex. Journal of Comparative Neurology, 307(3), 437–459. 1713237 10.1002/cne.903070308

[bib77] Zeineh M. M., Palomero-Gallagher N., Axer M., Gräβel D., Goubran M., Wree A., … Zilles K. (2017). Direct visualization and mapping of the spatial course of fiber tracts at microscopic resolution in the human hippocampus. Cerebral Cortex, 27(3), 1779–1794. 26874183 10.1093/cercor/bhw010 PMC5963820

[bib78] Zeki S., & Shipp S. (1988). The functional logic of cortical connections. Nature, 335(6188), 311–317. 3047584 10.1038/335311a0

[bib79] Zelle S. L., Gates K. M., Fiez J. A., Sayette M. A., & Wilson S. J. (2017). The first day is always the hardest: Functional connectivity during cue exposure and the ability to resist smoking in the initial hours of a quit attempt. NeuroImage, 151, 24–32. 26975550 10.1016/j.neuroimage.2016.03.015 PMC5018416

[bib80] Zhang K., Gong M., Ramsey J., Batmanghelich K., Spirtes P., & Glymour C. (2017). Causal discovery in the presence of measurement error: Identifiability conditions. Retrieved from https://www.cs.purdue.edu/homes/eb/causal-uai17/papers.html

[bib81] Zhang K., Peng H., Chan L., & Hyvärinen A. (2009). ICA with sparse connections: Revisited. In Adali T., Jutten C., Romano J. M. T., & Barros A. K. (Eds.), Independent Component Analysis and Signal Separation. ICA 2009. Lecture Notes in Computer Science (Vol. 5441). Berlin: Springer.

[bib82] Zou H. (2006). The Adaptive Lasso and Its Oracle Properties. Journal of the American Statistical Association, 101(476), 1418–1429.

