# Supplementary material for: Estimating feedforward and feedback effective connections from fMRI time series: Assessments of statistical methods
Source: Netw Neurosci. 2019 Feb 1;3(2):274–306. doi: 10.1162/netn_a_00061 (PMC6370458; doi:10.1162/netn_a_00061)
Supplement: Supplementary file 1 [file netn-03-274-s001.pdf]

Sanchez-Romero, R., Ramsey, J. D., Zhang, K., Glymour, M. R. K., Huang, B., & Glymour, C. (2019). Supporting Information for "Estimating feedforward and feedback effective connections from FMRI time series: Assessments of statistical methods." *Network Neuroscience, 3*(2), 274–306. https://doi.org/10.1162/netn_a_00061

Estimating Feedforward and Feedback Effective Connections from FMRI Time Series: Assessments of Statistical Methods

Sanchez-Romero, R., Ramsey, J. D., Zhang, K., Glymour, M. R. K., Huang, B., and Glymour, C.

Supplementary Material A

The FASK Algorithm

The Fast Adjacency Skewness (FASK) procedure is given as algorithms 1, 2, 3 and 4. The idea is as follows: First, we run Fast Adjacency Search stable (FAS-stable) on the data, producing an undirected graph. FAS-stable is the order independent adjacency search of the PC-stable algorithm (Spirtes et al., 2000; Colombo & Maathuis, 2014). We use the linear, Gaussian BIC score as a *conditional independence test* with a specified penalty discount (see section Methods: Cyclic Search Procedures, in the main text). This yields undirected graph G_0. The reason FAS-stable works for sparse cyclic models where the linear coefficients are all less than 1, is that correlations induced by long cyclic paths are statistically judged as zero, since they are products of multiple coefficients less than 1. One then orients each of the adjacencies in G_0 as a 2-cycle or a directed edge left or right. Taking up each adjacency in turn, one tests to see whether the adjacency is a 2-cycle using the 2-cycle Detection . If so, one adds edges $X \rightarrow Y$ and $X \leftarrow Y$ in the output graph G_1. If not, one applies the Left-Right orientation rule and orients $X \rightarrow Y$ or $X \leftarrow Y$ in the output graph G_1. G_1 is a fully oriented graph. For some models, where the true coefficients of a 2-cycle between X and Y are close but opposite in sign, a correlation test may eliminate the edge between X and Y when in fact a 2-cycle exists. In this case, we check explicitly whether $corr(X, Y|X > 0)$ and $corr(X, Y|Y > 0)$ differ by more than a set amount of 0.3. If so, we add the adjacency to the graph and orient it using the aforementioned rules. The idea for the heuristic extra adjacency rule is that if X and Y are independent, then $|corr(X, Y|X > 0) - corr(X, Y|Y > 0)| = 0$, so if this difference is not zero, there must be a trek between X and Y. If the difference is very different from zero, we infer a very short path and so add an adjacency. The specific threshold 0.3 is a parameter than can be tuned.

A comment on notation: When we write $E(X|X > 0)$ we mean $E(X)$ under the condition $X > 0$, and similarly for $corr(X, Y|X > 0)$. When we write $corr(X, Y|Z, X > 0)$ we mean $corr(X, Y|Z)$ under the condition $X > 0$.

Algorithm 1 FASK Algorithm

```
D a continuous dataset, c a penalty discount for a SEM-BIC score, α a threshold level for the 2-cycle
detection rule, δ a tunable parameter for the Left-Right rule
procedure FASK(D, c, α)                    ▷ Returns a fully oriented graph possibly with cycles.
    Center D
    G_0 ← FAS-stable(D, c)
    G_1 ← <>
    V ← variables in D
    for i = 1 to |V| − 1 do
        for j = i + 1 to |V| do
            X = ith variable in V
            Y = jth variable in V
            if adjacency X − Y is in G_0 or |corr(X, Y|X > 0) − corr(X, Y|Y > 0)| > 0.3 then
                if TwoCycle(D, X, Y, G_0, α) then
                    Add X → Y and X ← Y to G_1
                else if LeftRight(D, X, Y, δ) then
                    Add X → Y to G_1
                else
                    Add X ← Y to G_1
                end if
            end if
        end for
    end for
    return G_1
end procedure
```

Algorithm 2 FAS-stable Algorithm

```
D a continuous dataset, c a penalty discount for a SEM-BIC score.
procedure FAS-STABLE(D, c)
    V ← variables in D
    G_0 ← complete undirected graph over V
    for all depth = 0, 1, ..., so long as adjacencies can be removed do
        for all X ∈ V do
            a(X) ← adj(X, G_0)                    ▷ Adjacent variables set for X in G_0.
        end for
        for all X, Y, S for X, Y ∈ V, S ⊆ a(X) \ {Y}, |S| = depth do
            if X is independent of Y conditional on S then    ▷ Use SEM-BIC score with penalty c.
                Remove adjacency X − Y in G_0
            end if
        end for
    end for
    return G_0
end procedure
```

Algorithm 3 2-Cycle Detection Rule

D a continuous dataset, α a threshold level, G_0 an undirected graph, X, Y a pair of adjacent variables.
procedure TwoCycle(D, X, Y, G_0, α) ▷ Returns *true* if $X \rightleftarrows Y$.
  for all $\mathbf{S} \subseteq \{adj(X, G_0) \cup adj(Y, G_0)\} \setminus \{X, Y\}$ **do**
    $c \leftarrow corr(X, Y|\mathbf{S})$
    $c_1 \leftarrow corr(X, Y|\mathbf{S}, X > 0)$
    $c_2 \leftarrow corr(X, Y|\mathbf{S}, Y > 0)$
    $z \leftarrow 0.5(ln(1.0 + c) - ln(1.0 - c))$ ▷ Fisher's z-transformation.
    $z_1 \leftarrow 0.5(ln(1.0 + c_1) - ln(1.0 - c_1))$
    $z_2 \leftarrow 0.5(ln(1.0 + c_2) - ln(1.0 - c_2))$
    $n \leftarrow \#X$ ▷ $\#i$ sample size for variable i.
    $n_1 \leftarrow \#X > 0$
    $n_2 \leftarrow \#Y > 0$
    $zv_1 \leftarrow (z - z_1)/\sqrt{1/(n-3) + 1/(n_1 - 3)}$ ▷ Two-sample testing for diff. in correlations.
    $zv_2 \leftarrow (z - z_2)/\sqrt{1/(n-3) + 1/(n_2 - 3)}$
    $p_1 \leftarrow$ two-tailed $N(0, 1)$ p-value for zv_1
    $p_2 \leftarrow$ two-tailed $N(0, 1)$ p-value for zv_2
    $rejected_1 \leftarrow p_1 < \alpha$
    $rejected_2 \leftarrow p_2 < \alpha$
    $possibleTwoCycle \leftarrow false$
    if $rejected_1 \wedge rejected_2$ **then**
      $possibleTwoCycle \leftarrow true$
    else if $zv_1 < 0 \wedge zv_2 > 0 \wedge rejected_1$ **then** ▷ conditions in case of *control* 2-cycles.
      $possibleTwoCycle \leftarrow true$
    else if $zv_1 > 0 \wedge zv_2 < 0 \wedge rejected_2$ **then**
      $possibleTwoCycle \leftarrow true$
    end if
    if not $possibleTwoCycle$ **then**
      return $false$
    end if
  end for
  return $true$
end procedure

Algorithm 4 Left-Right Orientation Rule

D a continuous dataset, X, Y a pair of adjacent variables, δ in $(-1, 0)$ a tunable parameter.
procedure LeftRight(D, X, Y, δ) ▷ Returns *true* iff the edge should be oriented $X \to Y$.
  $e_1 \leftarrow E(XY|X > 0)/\sqrt{E(X^2|X > 0)E(Y^2|X > 0)}$
  $e_2 \leftarrow E(XY|Y > 0)/\sqrt{E(X^2|Y > 0)E(Y^2|Y > 0)}$
  $lr := e_1 - e_2$
  $c := corr(X, Y)$
  $sx := skewness(X)$
  $sy := skewness(Y)$
  $c := c * sign(sx) * sign(sy)$
  $lr := lr * sign(c)$
  if $c < \delta$ **then**
    $lr := -lr$
  end if
  return $lr > 0$
end procedure

The Left-Right Orientation Rule

Assuming $X \to Y$ or $X \leftarrow Y$ without a 2-cycle between X and Y, we give a procedure to orient the adjacency left or right. To do this, we define a certain relationship that must obtain if the probability distributions of the variables X, Y and their residuals, e_X and e_Y, are *smoothly positively skewed*, defined thus:

Definition 1. *We say a variable X with p.d.f., $f_X(x)$ centered around c is smoothly positively skewed if for every $b > 0$, $|\int_{c-b}^{c} x f_X(x)dx| > |\int_{c}^{c+b} x f_X(x)dx|$. X is smoothly negatively skewed if $-X$ is smoothly positively skewed.*

Variables that are smoothly positively skewed are positively skewed in Pearson's sense, $E[(X-\mu_X)^3]/(E[(X-\mu_X)^2])^{3/2} > 0$ (Pearson, 1894). There may be variables skewed positively in Pearson's sense that are are not smoothly positively skewed, as for instance case where $f_X(x)$ has a small bump directly to the right of the center c but is zero to the left within some interval $(c-b, c+b)$ but overall skewed in Pearson's sense. In such cases, for small b, it may be that $|\int_{c-b}^{c} x f_X(x)dx| < |\int_{c}^{c+b} x f_X(x)dx|$, whereas for sufficiently larger b, this will not hold. These cases we set aside.

We assume the following for pairwise direct causal relationships:

1. $Y = aX + e_Y$.
2. X and e_Y are independent: $X \perp\!\!\!\perp e_Y$.
3. X and e_Y are centered.
4. X is smoothly positively skewed.
5. e_Y is smoothly positively skewed.
6. $a > 0$.

Lemma 1. *Assume 1-6, then $E(Xe_Y|Y > 0) < 0$.*

Proof. See Figure A1 for X and eY centered around zero. The $E(Xe_Y|Y > 0)$ integrates over regions A, B, C and D, with $x_0, e_{y_0} > 0$. The total expectation can be decomposed into the expectation for regions A and B taken together and the expectation for regions C and D taken together. The expectation for regions A and B taken together is given by:

$$E(Xe_Y|Y > 0)_{A,B} = (\int_{-x_0}^{0} x f_X(x)dx + \int_{0}^{x_0} x f_X(x)dx)(\int_{|-ax|}^{e_{y_0}} e_y f_{e_Y}(e_y)de_y)$$

The sum inside the first parenthesis we know to be negative because of assumption 4 and definition 1. The term inside the second parenthesis is positive since e_y integrates over a positive range. Thus, the total product will be negative. Equivalently, for regions C ad D taken together:

$$E(Xe_Y|Y > 0)_{C,D} = (\int_{-e_{y_0}}^{0} e_y f_{e_Y}(e_y)de_y + \int_{0}^{e_{y_0}} e_y f_{e_Y}(e_y)de_y)(\int_{|-\frac{1}{a}e_{y_0}|}^{x_0} x f_X(x)dx)$$

Equivalently, by assumption 5 and definition 1 we know the sum inside parenthesis is negative, and the term inside the second parenthesis is positive since x integrates over a positive range. Thus, the total product is negative. Therefore, $E(Xe_Y|Y > 0) = E(Xe_Y|Y > 0)_{A,B} + E(Xe_Y|Y > 0)_{C,D} < 0$. □

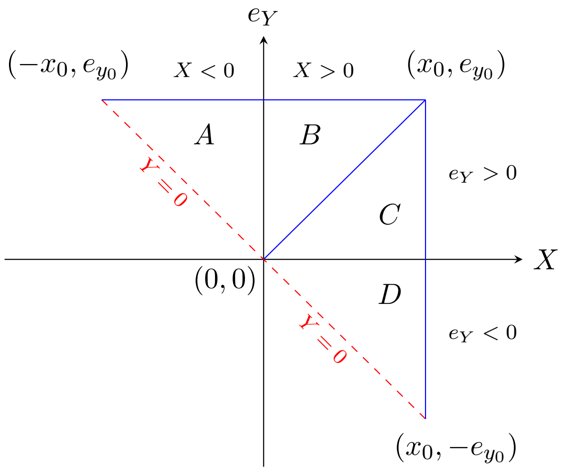

Figure A1: Integration regions A,B,C,D for $E(Xe_Y|Y > 0)$. The dashed line indicates $Y = 0 \Rightarrow e_Y = -aX$. The area to the right of the dashed line correspond to $Y > 0$.

Lemma 1 may be weakened in several ways. All that is necessary is that $E(Xe_Y|Y > 0)$ integrates to a negative number over regions A, B, C, and D. So if X is smoothly positively skewed but e_Y is symmetric about zero, this integral will still be negative, and the same is true in reverse. Also, clearly X or e_Y do not need to be smoothly positively skewed over their entire domains; if one or the other is smoothly positively skewed under any restriction of its domain, the conclusion will follow, so long as it is constant over the rest of its domain. In addition, small aberrations may be tolerated in the smooth skewness condition, so long as they are overpowered sufficiently soon in the integration.

Note that additional paths between X and Y do not alter the conclusion of Lemma 1, so long as assumptions 2, 3, 4, 5 and 6 are upheld. (Confounder paths do not satisfy these conditions, since X is not independent of e_Y in these cases.) Here, we simply use the *marginal* relationship between X, Y, and e_Y for Figure A1.

Lemma 2. *Assume 1-6, then* $\frac{E(XY|X>0)}{E(X^2|X>0)} > \frac{E(XY|Y>0)}{E(X^2|Y>0)}$.

Proof. Multiplying each term by X and taking expectations conditional on $Y > 0$ for $Y = aX + e_Y$, we have that $E(XY|Y > 0) - aE(X^2|Y > 0) = E(Xe_Y|Y > 0) < 0$, by Lemma 1, so

$$\frac{E(XY|Y>0)}{E(X^2|Y>0)} < a$$

If now we condition on $X > 0$ we have $E(XY|X > 0) - aE(X^2|X > 0) = E(Xe_Y|X > 0) = 0$ by assumption of $X \perp\!\!\!\perp e_Y$, so

$$\frac{E(XY|X>0)}{E(X^2|X>0)} = a$$

Consequently, $\frac{E(XY|X>0)}{E(X^2|X>0)} > \frac{E(XY|Y>0)}{E(X^2|Y>0)}$. □

Lemma 2 may be weakened as indicated for Lemma 1.

Theorem 1. *Assume 1-6, and additionally that Y and e_X are smoothly positively skewed and that X and Y do not form a 2-cycle, then*

$$\frac{E(XY|X>0)}{\sqrt{E(X^2|X>0)E(Y^2|X>0)}} > \frac{E(XY|Y>0))}{\sqrt{E(X^2|Y>0)E(Y^2|Y>0)}}$$

Proof. If $X \to Y$, we have by Lemma 2 that

$$A = \frac{E(XY|X>0)/E(X^2|X>0)}{E(XY|Y>0)/E(X^2|Y>0)} > 1$$

and following the same strategy as in Lemma 2 but multiplying the terms by Y instead of X, we can derive,

$$B = \frac{E(XY|Y>0)/E(Y^2|Y>0)}{E(XY|X>0)/E(Y^2|X>0)} < 1$$

This suggests a rule that if $A > B$ we orient $X \to Y$; if the reverse, $X \leftarrow Y$. $A > B$ simplifies to

$$\frac{E(XY|X>0)^2}{E(X^2|X>0)E(Y^2|X>0)} > \frac{E(XY|Y>0)^2}{E(X^2|Y>0)E(Y^2|Y>0)}$$

From this, the conclusion of the theorem follows. □

Theorem 1 may be weakened as indicated for Lemma 1.

A comment should be added as to how negative coefficients and negative skewness (violations of assumptions 4 - 6) can be handled. We may assume fairly in most cases that X and e_X are either both smoothly positively skewed together or both smoothly negatively skewed together, and that the same is true of Y and e_Y. Then if X and Y are both smoothly positively skewed and $a > 0$, Theorem 1 tells us that $X \to Y$. If $a < 0$, $E(Xe_Y)$ is negative for regions A and B and positive for the regions now symmetrically opposite to regions C and D. So whether the total integral for $E(Xe_Y)$ is positive or negative will depend on the value of a. There is a threshold δ, empirically tuned for particular types of data, for a greater than which this integral is negative and less than which it is positive. A δ of about -0.2 is suitable for our fMRI simulations. If $a < \delta$, then the orientation of the causality needs to be reversed. If X and Y are both smoothly negatively skewed, the same conclusion follows, since X and Y will both need to be replaced by their negations for Lemmas 1 and 2 and Theorem 1. For judging the δ threshold condition, one will need to replace X or Y or both by their negations when using correlation of X and Y as an estimate of the coefficient a for Lemmas 1, 2 and Theorem 1. The formulation of these ideas as an algorithm is given in Algorithm 4.

If the data is centered, the formulation in Theorem 1 is very close to the claim that $X \to Y$ if and only if $corr(X, Y|X > 0) > corr(X, Y|Y > 0)$. The latter claim will be true as well, if X and e_Y are not centered around zero but they are smoothly positively skewed. In practice, we find that the formulation in Theorem 1 works better.

The Left-Right rule is similar in many ways to the pairwise orientation rules proposed by Hyvärinen and Smith (2013), though differently motivated. It is closest to their Robust Skew rule substituting $h(x) = max(0, x)$ for $g(x) = log(cosh(max(0, x)))$, a very similar function. There are some differences suggested by the algebra. In simulation, it has a noticeable advantage for the data we are concerned with. Also, the assumptions of Theorem 1 may be weakened as indicated. For these reasons, we prefer it.

Left-Right Orientation Rule with Confounding

Although it is not exact, the above argument can be adjusted for the case where we have a linearly added confounding term like so,

$$Y = aX + bZ + e_Y \text{ and } X = cZ + e_X$$

Assume $X \perp\!\!\!\perp e_Y$, $Z \perp\!\!\!\perp e_X$; $a + bc > 0$; and Z, e_X and e_Y centered and smoothly positively skewed. In the equation for Y multiply each term by X and take expectations to get,

$$E(XY) = aE(X^2) + bcE(Z^2) + E(Xe_Y)$$

Conditioning on $X > 0$, together with the $X \perp\!\!\!\perp e_Y$ assumption, we have

$$E(XY|X > 0) = aE(X^2|X > 0) + bcE(Z^2|X > 0)$$

When conditioning on $Y > 0$ we get,

$$E(XY|Y > 0) = aE(X^2|Y > 0) + bcE(Z^2|Y > 0) + E(Xe_Y|Y > 0)$$

The question is if under the presence of a confounder, $E(XY|X > 0) > E(XY|Y > 0)$. This question reduces to whether

$$E(Z^2|X > 0) \approx E(Z^2|Y > 0)$$

Algebraically, these expressions are not identical, but the conclusion of Lemma 1 still either holds directly (in the cases we investigate in this paper) or is very close. As an example where one expects strong confounding, let the errors all be distributed as $Beta(1, 5)$ (which has a small variance and positive skew), and let coefficients a, b, c be drawn from $(-0.9, -0.1) \cup (0.1, 0.9)$, then over 100 runs of the simulation for this linear model, we have $mean(E(Z^2|X > 0)) = 0.0300$, $variance(E(Z^2|X > 0)) = 0.0002$, $mean(E(Z^2|Y > 0)) = 0.0377$, $variance(E(Z^2|Y > 0)) = 0.0003$, with a mean difference of -0.0077. If the product of the coefficients bc is positive, as is generally the case with our simulations, the break is in the right direction, but even if the product bc is negative, $bc(E(Z^2|X > 0) - E(Z^2|Y > 0))$ is quite small, since b and c are small in absolute value.

2-Cycle Detection Rule

In order to apply the Left-Right rule described above, we need to rule out the possibility that $X \to Y$ and $X \leftarrow Y$ forming a 2-cycle. Fortunately, we can detect 2-cycles using ideas from the Left-Right rule, though extra conditioning on combinations of adjacent variables needs to be done to block the effect of other possible cyclic paths between X and Y. The 2-cycle detection rule *without conditioning* only discovers whether a cyclic path exists between X and Y but cannot distinguish if such a path is a 2-cycle or a higher degree cycle.

The 2-cycle detection rule is based on the claim that if there are no additional cycles in the graph and $X \to Y$ and $X \leftarrow Y$, then $corr(X, Y) \neq corr(X, Y|X > 0)$ and $corr(X, Y) \neq corr(X, Y|Y > 0)$. Here, we use centered correlations as opposed to the uncentered correlations of Theorem 1 because the calculations are very close, and with centered correlations we can calculate z-statistics. As a result, the calculations are approximations, but very good ones.

The justification is based on arguments for the Left-Right rule. Assume X, Y, e_X, and e_Y centered and smoothly positively skewed. If $X \to Y$, with a linear model $Y = aX + e_Y$, $a > 0$, and $X \perp\!\!\!\perp e_Y$, then $E(XY)/E(X^2) = a$. Additionally, by Lemma 2 we know that $E(XY|X > 0)/E(X^2|X > 0) = a$, and $E(XY|Y > 0)/E(X^2|Y > 0) < a$. Considering these relationships, if $X \to Y$ then these two conditions will hold:

$$E(XY)/E(X^2) = E(XY|X > 0)/E(X^2|X > 0) \tag{1}$$

$$E(XY)/E(X^2) > E(XY|Y > 0)/E(X^2|Y > 0) \tag{2}$$

But for a 2-cycle, in which $X \to Y$ and $X \leftarrow Y$, with linear models $Y = aX + e_Y$ and $X = bY + e_X$, these two conditions do not hold anymore. Condition (1) fails because X is not independent of e_Y anymore, the necessary assumption for determining the equality. Condition (2) will still be an inequality but possibly with direction flipped depending on the relative strength of the coefficients. The important fact is that under a 2-cycle, conditions (1) and (2) will become inequalities that can be tested. In general, if $X \to Y$ and $X \leftarrow Y$, we expect:

$$E(XY)/E(X^2) \neq E(XY|X > 0)/E(X^2|X > 0) \quad (3)$$

$$E(XY)/E(X^2) \neq E(XY|Y > 0)/E(X^2|Y > 0) \quad (4)$$

We have seen in simulations two problems with testing (3) and (4) as they are. First, small decimal differences make the conditions to hold even without the presence of a 2-cycle. Second, the conditions may hold even without a 2-cycle if the sample is not large enough. To overcome these problems we approximate the ratios in (3) and (4) with conditional and unconditional correlations and perform statistical tests of the difference of correlations. In particular, using centered rather than non-centered correlations, we test if $corr(X, Y) \neq corr(X, Y|X > 0)$ and $corr(X, Y) \neq corr(X, Y|Y > 0)$.

Specifically, we convert these correlations to z-scores and make two-tailed hypothesis testing of the differences of the conditioned z-score with the unconditioned z-score for $X > 0$ and $Y > 0$ correspondingly. If both differences are judged significantly different from zero, we may conclude that X and Y form a 2-cycle.

The one problem with this approach is that the coefficients in opposite directions in the 2-cycle may have opposite signs, a type of cycle called *control cycle*. In control 2-cycles, one or the other of the conditioned correlations might not be distinguishable from the unconditioned correlation because of cancellation or near cancellation of coefficients. That is, one might reject the null of the difference test only for $X > 0$ or $Y > 0$, but not both, therefore missing the presence of a 2-cycle. However, in control 2-cycles the differences in correlations will be of opposite sign, unlike the unidirectional $X \to Y$ case, in which these differences will be of the same sign. Leveraging this information it is possible to distinguish a control 2-cycle from an unidirectional edge. The exact condition is given in the pseudo-code for Algorithm 3. Following this approach, all 2-cycles in a causal graph that contains no other cycles are identifiable.

If the graph contains other cyclic paths between X and Y, we simply need to condition on some combination of the adjacent variables of X and the adjacent variables of Y, other than X and Y themselves, to block additional d-connecting cyclic paths between X and Y. The theory for this is given in (Richardson, 1996), for the Cyclic Causal Discovery (CCD) algorithm. For the 2-cycle detection rule, we may try every combination of such adjacent variables, looking for a conditioning set where the 2-cycle condition above fails to obtain. If we find such a conditioning set, we stop and apply the Left-Right rule. If there is no such conditioning set, we conclude that the adjacency is a 2-cycle. This idea is incorporated into the pseudo-code for Algorithm 3.

Supplementary Material B

B1 Parameter Tuning

All the search procedures tested here require at least one user-input parameter; some require two or three. In order to give fair comparisons under parameters optimal for the respective algorithms and kind of data, datasets were created exactly as the ones described in the main text section Methods: Data Description, but changing the coefficient values range, by sampling them from a Gaussian distribution with $mean = 0.4$, $std.dev = 0.1$, and truncated between 0.2 and 0.6. We refer to these data as *training* datasets.

Ten datasets were selected at random without replacement from 60 individual training datasets for *Network 5* and *Network 7* exclusively. *Network 5* is a representative case for a structure with 2-cycles; and *Network 7* represents structures with higher degree cycles.

Each of the search algorithms was run on the training datasets with varying parameters. The Matthews correlation coefficient (MCC) (Matthews, 1975) computed from the confusion matrix, was used to assess the performance of the methods. The MCC ranges from +1, when an algorithm perfectly recovers the true graph without false positive edges, to -1, when the algorithm produces false positive edges without recovering any of the true edges in the graph. The average of the Matthews correlation coefficient (MCC) for adjacency estimation and orientation estimation was computed for each combination of parameters, and the best-performing parameters were selected. In cases where two or more combinations of parameters performed equally well, the combination which was present in the list of highest performances for both networks was selected. If multiple combinations appeared in both high-performance lists, each combination of parameters was used in the test phase. Matthews correlation coefficient contains an implicit utility trade-off between recall and precision that may not be appropriate for some settings.

For both implementations of Two-Step (FAS-stable and Alasso), multiple combinations of parameters performed equally well, but there was no overlap between the high-performing lists of parameters for *Network 5* and *7*, and thus no combination of parameters could be selected following the above procedure. To solve this problem, Two-Step with FAS-stable and with Alasso were run on ten randomly selected training datasets without replacement generated from the *SmallDegree* macaque network with coefficients sampled from a Gaussian distribution with $mean = 0.4$, $std.dev = 0.1$ and values truncated between 0.2 and 0.6. The highest-performing combination of parameters on this network was selected for both implementations of Two-Step.

Using the parameter values obtained under the process described above, all the algorithms were run on test data from *Networks 1* to *9* from Figure 1.

B2 Parameter Tuning for Macaque-Based Simulations

As noted in the main text section Methods: Data Description, for the macaque-based simulations the coefficients were restricted to very small values to guarantee the stability of the synthetic BOLD signals. Given this restriction on the coefficients we did not find it feasible to create a training set based on a different range for the coefficients, as we did in Section B1. Instead, we followed this procedure: We centered and concatenated the first ten datasets for each Macaque-based simulation, and used the concatenated data as training data. The remaining 50 individual datasets were used as the test set. FASK and Two-Step with Alasso were run for different parameters values and we chose those that maximize the average of the Matthews correlation coefficient for adjacencies and 2-cycle orientations in the case of FASK, and for adjacencies and the complete orientations for Two-Step, we explain the reason for this difference below. For FASK, the penalty discount c controls the sparsity of the adjacency search, while

the α threshold controls the sensitivity for the difference of correlations used in the the 2-cycle detection step. The left-right orientation rule does not require a parameter. Thus, we look for the pair of parameters that maximize the average of the Matthews correlation coefficient for adjacencies and 2-cycles (MCCa2c). This process is illustrated in Figure B1, with heatmaps for the MCCa2c values obtained under different combinations of values for the penalty discount c and the α threshold for 2-cycle detection, for each of the four macaque-based networks.

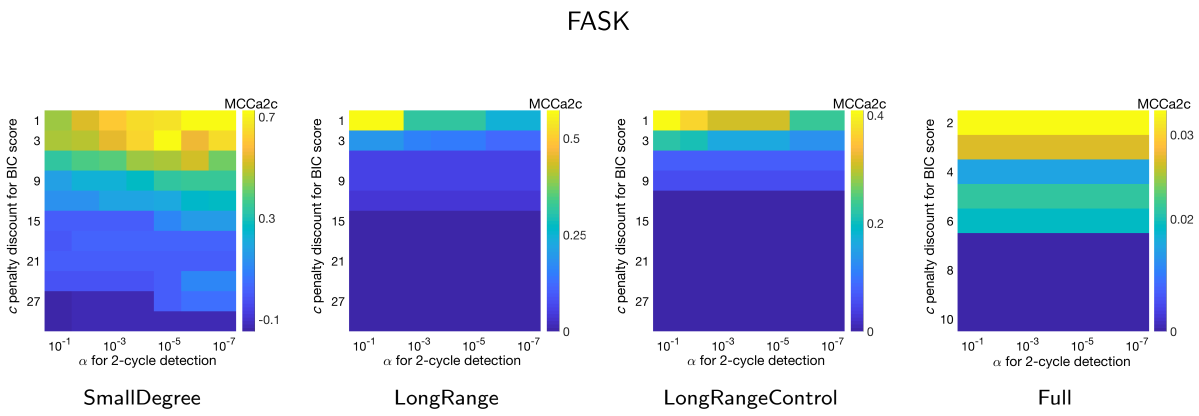

Figure B1: Average Matthews correlation coefficient for adjacencies and 2-cycles (MCCa2c) for the FASK algorithm, under different combinations of c penalty discount for the BIC* score (Y axis) and α significance threshold for the 2-cycle detection rule (X axis). Results for the four macaque-based simulations.

For Two-Step with Alasso, we vary the sparsity penalty λ of the $\mathbf{B}$ matrix estimation, and the threshold for the absolute values in the final $\mathbf{B}$ matrix. In contrast to FASK, the detection of 2-cycles is part of the global ICA estimation procedure of Two-Step, and so we use the average of the Matthews correlation coefficient for adjacencies and orientations (MCCao). Figure B2 shows the parameter tuning heatmaps for combinations of λ and the threshold for the absolute values of the $\mathbf{B}$ matrix, for each of the four macaque-based networks simulations.

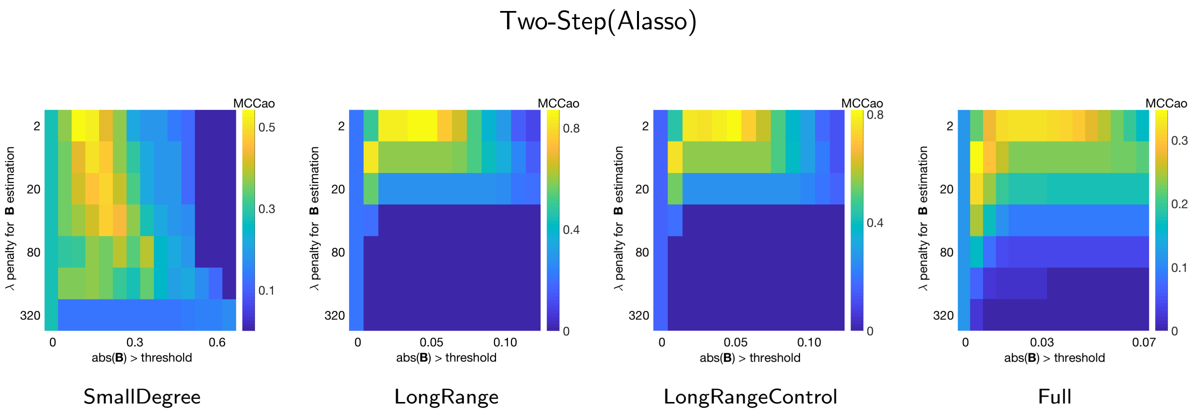

Figure B2: Average Matthews correlation coefficient for adjacencies and orientations (MCCao) for the Two-Step(Alasso) algorithm, under different combinations of the sparsity parameter λ (Y axis) and the threshold for the absolute $\mathbf{B}$ matrix values (X axis). Results for the four macaque-based simulations.

Supplementary Material C

C1 Results for simple networks with 2-cycles

Complete results for analysis shown in Figure 2 of the main text, for *Networks 1* to *6* and their amplifying and control variants. "amp" and "cont" after each network label indicates if the network has an amplifying (excitatory) or a control (inhibitory) cycle. For *Network 5* two additional control variants were tested, one where the 2-cycle is a control cycle with coefficients +0.3 and −0.7 (Net5_c_p3n7); and its inverse where the 2-cycle coefficients are +0.7 and −0.3 (Net5_c_p7n3). Tables show for each algorithm tested the average results for 60 repetitions of ten concatenated datasets from the corresponding networks, for precision and recall of adjacencies, orientations, and 2-cycle detection, and running times in seconds. The average and standard deviation over all ten networks for each algorithm, is presented at the right of each average table. These averages of averages are plotted in Figure 2. Corresponding tables showing the standard deviation across 60 repetitions of ten concatenated datasets for each network and algorithm are also presented.

	Net1_amp	Net2_amp	Net3_amp	Net4_amp	Net5_amp	Net5_cont	Net5_c_p3n7	Net5_c_p7n3	Net6_amp	Net6_cont	average	std.dev
MVGC	0.7	0.55	0.67	0.7	0.79	0.86	0.84	0.91	0.86	0.89	0.78	0.12
MVARp	0.81	0.68	0.76	0.60	0.81	0.84	0.81	0.88	0.87	0.86	0.79	0.09
GIMME-50	0.84	0.77	0.81	0.76	0.92	0.96	0.97	0.93	0.91	0.99	0.89	0.08
GIMME-60	0.88	0.81	0.84	0.78	0.95	0.99	0.99	1	0.93	1	0.92	0.08
GlobalMIT(FAS)	0.99	0.94	0.93	0.99	0.97	1	1	1	1	1	0.98	0.03
CCD-max	0.95	0.85	0.85	0.97	0.83	0.62	0.83	0.71	0.98	0.74	0.83	0.12
FAS+R1	0.95	0.85	0.85	0.97	0.83	0.62	0.83	0.71	0.98	0.74	0.83	0.12
FAS+R2	0.95	0.85	0.85	0.97	0.83	0.62	0.83	0.71	0.98	0.74	0.83	0.12
FAS+R3	0.95	0.85	0.85	0.97	0.83	0.62	0.83	0.71	0.98	0.74	0.83	0.12
FAS+Patel	0.95	0.85	0.85	0.97	0.83	0.62	0.83	0.71	0.98	0.74	0.83	0.12
FAS+Skew	0.95	0.85	0.85	0.97	0.83	0.62	0.83	0.71	0.98	0.74	0.83	0.12
FAS+RSkew	0.95	0.85	0.85	0.97	0.83	0.62	0.83	0.71	0.98	0.74	0.83	0.12
FASK	0.95	0.85	0.85	0.97	0.83	0.68	0.83	0.75	0.98	0.77	0.85	0.10
Two-Step(FAS)	0.89	0.78	0.81	0.88	0.9	0.75	0.81	0.79	0.91	0.79	0.83	0.06
Two-Step(Alasso)	0.93	0.76	0.87	0.92	0.88	0.88	0.76	1	0.97	0.88	0.89	0.08

Table C1: Adjacency Precision (average)

	Net1_amp	Net2_amp	Net3_amp	Net4_amp	Net5_amp	Net5_cont	Net5_c_p3n7	Net5_c_p7n3	Net6_amp	Net6_cont
MVGC	0.07	0.04	0.05	0.06	0.03	0.14	0.1	0.14	0.06	0.09
MVARp	0.12	0.11	0.13	0.10	0.10	0.15	0.08	0.13	0.09	0.10
GIMME-50	0.11	0.09	0.11	0.06	0.1	0.09	0.07	0.12	0.07	0.04
GIMME-60	0.11	0.08	0.1	0.07	0.1	0.05	0.05	0.03	0.07	0.02
GloablMIT(FAS)	0.04	0.10	0.13	0.03	0.11	0	0	0	0	0
CCD-max	0.07	0.09	0.05	0.04	0.09	0.06	0.11	0.1	0.04	0.05
FAS+R1	0.07	0.08	0.05	0.05	0.09	0.06	0.11	0.1	0.04	0.05
FAS+R2	0.07	0.08	0.05	0.05	0.09	0.06	0.11	0.1	0.04	0.05
FAS+R3	0.07	0.08	0.05	0.05	0.09	0.06	0.11	0.1	0.04	0.05
FAS+Patel	0.07	0.08	0.05	0.05	0.09	0.06	0.11	0.1	0.04	0.05
FAS+Skew	0.07	0.08	0.05	0.05	0.09	0.06	0.11	0.1	0.04	0.05
FAS+Rskew	0.07	0.08	0.05	0.05	0.09	0.06	0.11	0.1	0.04	0.05
FASK	0.08	0.08	0.05	0.04	0.08	0.04	0.08	0.09	0.05	0.05
Two-Step(FAS)	0.05	0.08	0.08	0.05	0.04	0.15	0.12	0.13	0.02	0.08
Two-Step(Alasso)	0.09	0.09	0.07	0.06	0.1	0.16	0.08	0	0.05	0.13

Table C2: Adjacency Precision (standard deviation)

	Net1_amp	Net2_amp	Net3_amp	Net4_amp	Net5_amp	Net5_cont	Net5_c_p3n7	Net5_c_p7n3	Net6_amp	Net6_cont	average	std.dev
MVGC	1	1	1	0.95	1	0.75	1	0.86	1	0.86	0.94	0.09
MVARp	0.99	0.99	0.98	0.93	1	0.76	0.98	0.89	1	0.86	0.94	0.08
GIMME-50	1	1	1	0.87	0.99	0.75	0.97	0.84	1	0.85	0.93	0.09
GIMME-60	0.99	1	0.97	0.82	0.98	0.71	0.85	0.74	0.99	0.83	0.89	0.11
GlobalMIT(FAS)	0.77	0.81	0.64	0.46	0.51	0.38	0.52	0.36	0.7	0.47	0.56	0.16
CCD-max	1	1	1	0.85	1	0.75	1	0.84	1	0.86	0.93	0.09
FAS+R1	1	1	1	0.85	1	0.75	1	0.84	1	0.86	0.93	0.09
FAS+R2	1	1	1	0.85	1	0.75	1	0.84	1	0.86	0.93	0.09
FAS+R3	1	1	1	0.85	1	0.75	1	0.84	1	0.86	0.93	0.09
FAS+Patel	1	1	1	0.85	1	0.75	1	0.84	1	0.86	0.93	0.09
FAS+Skew	1	1	1	0.85	1	0.75	1	0.84	1	0.86	0.93	0.09
FAS+RSkew	1	1	1	0.85	1	0.75	1	0.84	1	0.86	0.93	0.09
FASK	1	1	1	0.85	1	1	1	0.99	1	1	0.98	0.05
Two-Step(FAS)	0.91	0.9	0.91	0.75	0.91	0.68	0.85	0.77	0.91	0.78	0.84	0.09
Two-Step(Alasso)	1	1	1	0.8	0.99	0.85	1	1	1	0.89	0.95	0.08

Table C3: Adjacency Recall (average)

	Net1_amp	Net2_amp	Net3_amp	Net4_amp	Net5_amp	Net5_cont	Net5_c_p3n7	Net5_c_p7n3	Net6_amp	Net6_cont
MVGC	0	0	0	0.05	0	0.03	0	0.12	0	0
MVARp	0.04	0.04	0.06	0.07	0	0.05	0.06	0.13	0	0.02
GIMME-50	0.03	0	0.03	0.06	0.05	0.06	0.08	0.13	0.02	0.03
GIMME-60	0.04	0.03	0.07	0.07	0.07	0.1	0.19	0.16	0.04	0.06
GlobalMIT(FAS)	0.11	0.13	0.16	0.06	0.11	0.18	0.17	0.21	0.05	0.11
CCD-max	0	0	0	0.05	0	0	0	0.12	0	0
FAS+R1	0	0	0	0.05	0	0	0	0.12	0	0
FAS+R2	0	0	0	0.05	0	0	0	0.12	0	0
FAS+R3	0	0	0	0.05	0	0	0	0.12	0	0
FAS+Patel	0	0	0	0.05	0	0	0	0.12	0	0
FAS+Skew	0	0	0	0.05	0	0	0	0.12	0	0
FAS+Rskew	0	0	0	0.05	0	0	0	0.12	0	0
FASK	0	0	0	0.06	0	0	0	0.05	0	0
Two-Step(FAS)	0	0.03	0	0.06	0	0	0.11	0.12	0	0
Two-Step(Alasso)	0	0	0.03	0.08	0.05	0.15	0.03	0	0	0.1

Table C4: Adjacency Recall (standard deviation)

	Net1_amp	Net2_amp	Net3_amp	Net4_amp	Net5_amp	Net5_cont	Net5_c_p3n7	Net5_c_p7n3	Net6_amp	Net6_cont	average	std.dev
MVGC	0.45	0.41	0.5	0.52	0.52	0.5	0.56	0.51	0.53	0.47	0.50	0.04
MVARp	0.53	0.55	0.61	0.43	0.55	0.58	0.58	0.53	0.52	0.45	0.53	0.06
GIMME-50	0.57	0.60	0.64	0.52	0.69	0.74	0.72	0.62	0.70	0.57	0.64	0.07
GIMME-60	0.56	0.59	0.65	0.52	0.71	0.77	0.74	0.64	0.72	0.59	0.65	0.08
GlobalMIT(FAS)	0.93	0.91	0.95	0.89	0.93	0.94	0.96	0.94	0.96	0.85	0.93	0.03
CCD-max	0.36	0.42	0.51	0.60	0.09	0.27	0.66	0.34	0.99	0.43	0.47	0.25
FAS+R1	0.94	0.68	0.80	0.84	0.55	0.58	0.69	0.72	0.95	0.73	0.75	0.14
FAS+R2	0.95	0.78	0.82	0.76	0.76	0.68	0.94	0.96	0.98	0.98	0.86	0.11
FAS+R3	0.94	0.55	0.79	0.54	0.83	0.62	0.83	0.71	0.98	0.74	0.75	0.15
FAS+Patel	0.83	0.81	0.85	0.95	0.63	0.41	0.62	0.51	0.58	0.55	0.67	0.18
FAS+Skew	0.95	0.85	0.85	0.97	0.83	0.62	0.69	0.71	0.98	0.74	0.82	0.13
FAS+RSkew	0.95	0.85	0.85	0.97	0.83	0.62	0.83	0.71	0.98	0.74	0.83	0.12
FASK	0.96	0.88	0.89	0.97	0.86	0.70	0.77	0.78	0.98	0.79	0.86	0.10
Two-Step(FAS)	0.87	0.76	0.80	0.69	0.90	0.73	0.77	0.79	0.86	0.79	0.80	0.07
Two-Step(Alasso)	0.78	0.72	0.85	0.73	0.87	0.79	0.70	0.93	0.91	0.82	0.81	0.08

Table C5: Orientation Precision (average)

	Net1_amp	Net2_amp	Net3_amp	Net4_amp	Net5_amp	Net5_cont	Net5_c_p3n7	Net5_c_p7n3	Net6_amp	Net6_cont
MVGC	0.05	0.03	0	0.04	0.04	0.1	0.05	0.07	0.03	0.03
MVARp	0.08	0.1	0.1	0.07	0.09	0.1	0.1	0.08	0.05	0.06
GIMME-50	0.24	0.18	0.2	0.11	0.18	0.2	0.18	0.31	0.13	0.2
GIMME-60	0.26	0.2	0.2	0.12	0.18	0.2	0.2	0.32	0.14	0.2
GlobalMIT(FAS)	0.12	0.12	0.1	0.08	0.13	0.2	0.12	0.15	0.07	0.14
CCD-max	0.06	0.07	0	0.07	0.09	0.1	0.15	0.19	0.1	0.19
FAS+R1	0.05	0.09	0.1	0.03	0.1	0.1	0.09	0.1	0.08	0.05
FAS+R2	0.07	0.12	0.1	0.05	0.07	0	0.08	0.06	0.05	0.04
FAS+R3	0.09	0.12	0.1	0.05	0.09	0.1	0.11	0.1	0.04	0.05
FAS+Patel	0.11	0.09	0.1	0.06	0.08	0	0.09	0.09	0.09	0.1
FAS+Skew	0.07	0.08	0.1	0.05	0.09	0.1	0.14	0.1	0.04	0.05
FAS+Rskew	0.07	0.08	0.1	0.05	0.09	0.1	0.11	0.1	0.04	0.05
FASK	0.07	0.07	0	0.04	0.06	0.1	0.1	0.08	0.04	0.04
Two-Step(FAS)	0.07	0.1	0.1	0.08	0.05	0.2	0.15	0.14	0.07	0.08
Two-Step(Alasso)	0.1	0.09	0.1	0.09	0.1	0.2	0.09	0.1	0.09	0.15

Table C6: Orientation Precision (standard deviation)

	Net1_amp	Net2_amp	Net3_amp	Net4_amp	Net5_amp	Net5_cont	Net5_c_p3n7	Net5_c_p7n3	Net6_amp	Net6_cont	average	std.dev
MVGC	0.99	1	0.99	0.9	0.99	0.59	0.96	0.69	0.99	0.74	0.88	0.15
MVARp	0.91	0.91	0.89	0.72	0.86	0.56	0.79	0.68	0.89	0.64	0.79	0.13
GIMME-50	0.62	0.61	0.63	0.5	0.68	0.46	0.63	0.46	0.73	0.43	0.58	0.10
GIMME-60	0.54	0.54	0.55	0.44	0.64	0.44	0.53	0.39	0.7	0.43	0.52	0.10
GlobalMIT(FAS)	0.77	0.82	0.73	0.5	0.58	0.29	0.44	0.32	0.73	0.4	0.56	0.20
CCD-max	0.28	0.17	0.4	0.42	0.03	0.24	0.3	0.33	0.86	0.23	0.33	0.22
FAS+R1	0.67	0.25	0.33	0.09	0.36	0.53	0.54	0.66	0.58	0.75	0.48	0.21
FAS+R2	0.73	0.54	0.6	0.26	0.28	0.07	0.39	0.17	0.52	0.5	0.41	0.21
FAS+R3	0.82	0.46	0.66	0.37	0.8	0.6	0.8	0.67	0.88	0.75	0.68	0.16
FAS+Patel	0.73	0.69	0.71	0.66	0.61	0.4	0.6	0.48	0.52	0.56	0.6	0.11
FAS+Skew	0.83	0.71	0.71	0.67	0.8	0.6	0.67	0.67	0.88	0.75	0.73	0.09
FAS+RSkew	0.83	0.71	0.71	0.67	0.8	0.6	0.8	0.67	0.88	0.75	0.74	0.09
FASK	1	0.99	1	0.88	1	0.93	0.81	0.97	1	1	0.96	0.07
Two-Step(FAS)	0.9	0.88	0.88	0.68	0.91	0.54	0.71	0.62	0.91	0.68	0.77	0.14
Two-Step(Alasso)	0.99	0.96	1	0.71	0.99	0.69	0.8	0.8	1	0.79	0.87	0.13

Table C7: Orientation Recall (average)

	Net1_amp	Net2_amp	Net3_amp	Net4_amp	Net5_amp	Net5_cont	Net5_c_p3n7	Net5_c_p7n3	Net6_amp	Net6_cont
MVGC	0.04	0	0.04	0.06	0.05	0.05	0.08	0.1	0.03	0.03
MVARp	0.12	0.12	0.12	0.08	0.17	0.09	0.15	0.13	0.1	0.08
GIMME-50	0.25	0.16	0.16	0.11	0.18	0.12	0.15	0.23	0.14	0.15
GIMME-60	0.24	0.16	0.14	0.1	0.17	0.13	0.16	0.22	0.13	0.15
GlobalMIT(FAS)	0.11	0.13	0.11	0.05	0.07	0.13	0.14	0.19	0.05	0.1
CCD-max	0.1	0.18	0.13	0.05	0.1	0.2	0.17	0.22	0.07	0.25
FAS+R1	0	0.15	0.15	0.04	0.19	0.1	0.09	0.11	0.11	0.03
FAS+R2	0.08	0.13	0.1	0.09	0.12	0.13	0.13	0.15	0.07	0
FAS+R3	0.04	0.08	0.07	0.04	0	0	0	0.1	0	0
FAS+Patel	0.08	0.06	0	0.05	0.04	0	0	0.1	0.08	0.09
FAS+Skew	0	0	0	0.04	0	0.03	0.1	0.1	0	0
FAS+Rskew	0	0	0	0.04	0	0	0	0.1	0	0
FASK	0	0.02	0	0.05	0	0.1	0.06	0.09	0	0
Two-Step(FAS)	0.03	0.06	0.06	0.08	0	0	0.18	0.1	0	0
Two-Step(Alasso)	0.03	0.07	0.03	0.1	0.05	0.12	0.04	0.03	0	0.13

Table C8: Orientation Recall (standard deviation)

	Net1_amp	Net2_amp	Net3_amp	Net4_amp	Net5_amp	Net5_cont	Net5_c_p3n7	Net5_c_p7n3	Net6_amp	Net6_cont	average	std.dev
MVGC	0.2	0.3	0.3	0.33	0.2	0	0.23	0	0.14	0	0.17	0.13
MVARp	0.3	0.4	0.5	0.44	0.3	0	0.21	0	0.2	0	0.24	0.20
GIMME-50	0.3	0.4	0.4	0.55	0.5	0	0	0	0.27	0	0.24	0.23
GIMME-60	0.2	0.2	0.2	0.27	0.4	0	0	0	0.1	0	0.13	0.13
GlobalMIT(FAS)	0.9	1	1	0.97	1	0	0.64	0.5	0.9	0	0.68	0.39
CCD-max	0	0	0	0	0	1	1	1	0	0	0.3	0.48
FAS+R1	0	0	0	0.04	0	0	0	0	0	0	0	0.01
FAS+R2	*	*	*	*	*	*	*	*	*	*	*	*
FAS+R3	*	*	*	*	*	*	*	*	*	*	*	*
FAS+Patel	*	*	*	*	*	*	*	*	*	*	*	*
FAS+Skew	*	*	*	*	*	*	*	*	*	*	*	*
FAS+RSkew	*	*	*	*	*	*	*	*	*	*	*	*
FASK	1	1	1	0.99	1	0.89	0.12	1	1	1	0.9	0.28
Two-Step(FAS)	0.9	0.8	0.8	0.51	0.9	0	0.5	0	0.8	0	0.51	0.38
Two-Step(Alasso)	0.5	0.7	0.8	0.64	0.9	0.03	0.02	0	0.7	0.2	0.45	0.36

Table C9: 2-cycle Precision (average). The (*) symbol indicates algorithms that cannot detect 2-cycles by design: R2, R3, Patel, Skew and RSkew.

	Net1_amp	Net2_amp	Net3_amp	Net4_amp	Net5_amp	Net5_cont	Net5_c_p3n7	Net5_c_p7n3	Net6_amp	Net6_cont
MVGC	0	0	0	0.04	0	0	0.09	0	0	0
MVARp	0.1	0.2	0.1	0.08	0.1	0	0.24	0	0	0
GIMME-50	0.4	0.4	0.4	0.37	0.4	0	0	0	0.4	0
GIMME-60	0.3	0.3	0.3	0.38	0.4	0	0	-	0.3	-
GlobalMIT(FAS)	0.2	0.1	0	0.08	0.1	0	0.5	0.5	0.2	0
CCD-max	-	-	-	-	-	-	-	-	-	-
FAS+R1	-	-	-	-	-	-	-	-	-	-
FAS+R2	*	*	*	*	*	*	*	*	*	*
FAS+R3	*	*	*	*	*	*	*	*	*	*
FAS+Patel	*	*	*	*	*	*	*	*	*	*
FAS+Skew	*	*	*	*	*	*	*	*	*	*
FAS+Rskew	*	*	*	*	*	*	*	*	*	*
FASK	0	0	0	0.03	0	0.28	0.3	0	0	0
Two-Step(FAS)	0.2	0.2	0.2	0.15	0.1	0	0.39	0	0.3	0
Two-Step(Alasso)	0.3	0.2	0.2	0.14	0.2	0.18	0.13	0.1	0.3	0.3

Table C10: 2-cycle Precision (standard deviation). The (*) symbol indicates algorithms that cannot detect 2-cycles by design: R2, R3, Patel, Skew and RSkew.

	Net1_amp	Net2_amp	Net3_amp	Net4_amp	Net5_amp	Net5_cont	Net5_c_p3n7	Net5_c_p7n3	Net6_amp	Net6_cont	average	std.dev
MVGC	1	1	1	1	1	0	0.88	0	1	0	0.69	0.48
MVARp	1	0.91	1	1	0.9	0	0.48	0	1	0	0.62	0.45
GIMME-50	0.3	0.21	0.3	0.2	0.6	0	0	0	0.3	0	0.18	0.18
GIMME-60	0.2	0.09	0.1	0.1	0.4	0	0	0	0.2	0	0.09	0.12
GlobalMIT(FAS)	1	0.93	1	0.9	1	0	0.12	0.13	1	0	0.60	0.47
CCD-max	0	0	0	0	0	0.02	0.02	0.08	0	0	0.01	0.03
FAS+R1	0	0	0	0	0	0	0	0	0	0	0	0
FAS+R2	*	*	*	*	*	*	*	*	*	*	*	*
FAS+R3	*	*	*	*	*	*	*	*	*	*	*	*
FAS+Patel	*	*	*	*	*	*	*	*	*	*	*	*
FAS+Skew	*	*	*	*	*	*	*	*	*	*	*	*
FAS+RSkew	*	*	*	*	*	*	*	*	*	*	*	*
FASK	1	0.99	1	1	1	0.66	0.06	0.91	1	1	0.86	0.30
Two-Step(FAS)	0.9	0.88	0.8	0.7	0.9	0	0.52	0	0.9	0	0.57	0.41
Two-Step(Alasso)	1	0.93	1	0.9	1	0.03	0.02	0.02	1	0.2	0.60	0.46

Table C11: 2-cycle Recall (average). The (*) symbol indicates algorithms that cannot detect 2-cycles by design: R2, R3, Patel, Skew and RSkew.

	Net1_amp	Net2_amp	Net3_amp	Net4_amp	Net5_amp	Net5_cont	Net5_c_p3n7	Net5_c_p7n3	Net6_amp	Net6_cont
MVGC	0	0	0	0	0	0	0.32	0	0	0
MVARp	0.13	0.27	0	0.10	0.28	0	0.50	0	0.13	0
GIMME-50	0.5	0.25	0.3	0.2	0.5	0	0	0	0.5	0
GIMME-60	0.4	0.2	0.2	0.1	0.5	0	0	0	0.4	0
GlobalMIT(FAS)	0.18	0.17	0.09	0.12	0.00	0.00	0.32	0.34	0.13	0.00
CCD-max	0	0	0	0	0	0	0.22	0.18	0	0
FAS+R1	0	0	0	0	0	0	0	0	0	0
FAS+R2	*	*	*	*	*	*	*	*	*	*
FAS+R3	*	*	*	*	*	*	*	*	*	*
FAS+Patel	*	*	*	*	*	*	*	*	*	*
FAS+Skew	*	*	*	*	*	*	*	*	*	*
FAS+Rskew	*	*	*	*	*	*	*	*	*	*
FASK	0	0.06	0	0	0	0.48	0.25	0.28	0	0
Two-Step(FAS)	0.2	0.13	0.2	0.2	0	0	0.49	0	0	0
Two-Step(Alasso)	0.2	0.17	0.1	0.2	0.2	0.18	0.13	0.13	0	0.4

Table C12: 2-cycle Recall (standard deviation). The (*) symbol indicates algorithms that cannot detect 2-cycles by design: R2, R3, Patel, Skew and RSkew.

	Net1_amp	Net2_amp	Net3_amp	Net4_amp	Net5_amp	Net5_cont	Net5_c_p3n7	Net5_c_p7n3	Net6_amp	Net6_cont	average	std.dev
MVGC	0.13	0.11	0.12	0.39	0.10	0.10	0.11	0.10	0.23	0.17	0.15	0.09
MVARp	16.83	15.42	15.8	61.29	16.05	15.88	15.78	15.46	38.6	38.8	25.0	15.89
GIMME-50	44.0	54.8	49.4	446.5	42.6	28.8	34.6	57.8	199.2	206.8	116.5	133.64
GIMME-60	101.2	72.7	87.7	766.7	36.1	27.0	30.9	27.5	108.0	140.2	139.8	223.78
GlobalMIT(FAS)	0.41	0.67	0.68	3.37	0.58	0.42	0.58	0.52	1.54	1.64	1.04	0.93
CCD-max	0.00	0.00	0.00	0.01	0.00	0.00	0.00	0.00	0.00	0.00	0.002	0.00
FAS+R1	0.11	0.13	0.12	0.44	0.10	0.10	0.11	0.10	0.18	0.20	0.16	0.10
FAS+R2	0.74	1.26	1.14	6.20	0.90	0.69	0.91	0.77	1.67	2.15	1.64	1.67
FAS+R3	0.10	0.11	0.11	0.32	0.09	0.09	0.09	0.08	0.14	0.15	0.13	0.07
FAS+Patel	0.01	0.02	0.02	0.12	0.01	0.01	0.01	0.01	0.03	0.02	0.03	0.03
FAS+Skew	0.01	0.01	0.01	0.11	0.01	0.01	0.01	0.01	0.02	0.01	0.02	0.03
FAS+RSkew	0.01	0.01	0.01	0.12	0.01	0.01	0.01	0.01	0.02	0.02	0.02	0.03
FASK	0.04	0.04	0.03	0.24	0.02	0.02	0.02	0.02	0.05	0.04	0.05	0.07
Two-Step(FAS)	22.45	59.00	28.86	54.80	9.50	6.08	16.95	6.92	11.83	9.47	22.59	19.47
Two-Step(Alasso)	33.78	79.31	59.74	110.59	32.39	29.56	27.97	20.20	70.95	30.42	49.49	29.45

Table C13: Running Time (seconds) (average)

	Net1_amp	Net2_amp	Net3_amp	Net4_amp	Net5_amp	Net5_cont	Net5_c_p3n7	Net5_c_p7n3	Net6_amp	Net6_cont
MVGC	0.03	0.01	0.02	0.04	0.01	0.01	0.01	0.01	0.04	0.02
MVARp	1.16	0.35	0.67	1.87	0.70	0.60	0.57	0.69	1.10	1.76
GIMME-50	5.25	9.12	10.70	53.77	7.71	2.75	3.73	15.65	39.02	34.70
GIMME-60	36.33	10.92	34.44	326.92	4.15	1.83	3.33	2.07	11.30	5.43
GlobalMIT(FAS)	0.21	0.25	0.19	1.20	0.13	0.19	0.16	0.38	0.19	0.19
CCD-max	0.00	0.00	0.00	0.00	0.00	0.00	0.00	0.00	0.00	0.00
FAS+R1	0.02	0.02	0.01	0.05	0.01	0.01	0.02	0.02	0.02	0.01
FAS+R2	0.29	0.62	0.22	2.60	0.19	0.13	0.36	0.34	0.83	0.24
FAS+R3	0.29	0.62	0.22	2.60	0.19	0.13	0.36	0.34	0.83	0.24
FAS+Patel	0.00	0.00	0.00	0.01	0.00	0.00	0.00	0.00	0.00	0.00
FAS+Skew	0.00	0.00	0.00	0.00	0.00	0.00	0.00	0.00	0.00	0.00
FAS+RSkew	0.00	0.00	0.00	0.00	0.00	0.00	0.00	0.00	0.00	0.00
FASK	0.16	0.01	0.01	0.03	0.01	0.01	0.01	0.01	0.01	0.01
Two-Step(FAS)	18.77	8.97	14.85	27.24	6.49	2.57	15.18	2.67	4.70	1.44
Two-Step(Alasso)	18.97	18.49	26.13	21.74	14.78	8.67	5.27	2.94	10.13	8.68

Table C14: Running Time (seconds) (standard deviation)

C2 Results for simple networks with higher degree cycles

Complete results for analysis shown in Figure 3 of the main text, for *Networks 7* to *9* and their amplifying and control variants. "amp" and "cont" after each network label indicates if the network has an amplifying (excitatory) or a control (inhibitory) cycle. Tables show for each algorithm tested the average results over 60 repetitions of ten concatenated datasets from the

corresponding simulations, for precision and recall of adjacencies, orientations, and 2-cycle false positives; and running times in seconds. Average over all eight networks for each algorithm is shown to the right of each table, followed by the corresponding standard deviation. These averages are plotted in Figure 3.

	Net7_amp	Net7_cont	Net8_a_a	Net8_a_c	Net8_c_a	Net9_a_a	Net9_a_c	Net9_c_a	average	std.dev
MVGC	0.79	0.99	0.79	0.83	0.93	0.75	0.87	0.91	0.86	0.08
MVARp	0.86	0.99	0.84	0.88	0.9	0.76	0.89	0.92	0.88	0.07
GIMME-50	0.89	0.99	0.88	0.93	0.98	0.85	0.93	0.93	0.92	0.05
GIMME-60	0.92	1	0.91	0.95	0.99	0.88	0.95	0.95	0.94	0.04
GlobalMIT(FAS)	0.99	1	1	1	1	1	1	1	1	0.00
CCD-max	0.97	1	1	0.99	1	0.96	0.98	1	0.99	0.02
FAS+R1	0.97	1	1	0.99	1	0.96	0.98	1	0.99	0.02
FAS+R2	0.97	1	1	0.99	1	0.96	0.98	1	0.99	0.02
FAS+R3	0.97	1	1	0.99	1	0.96	0.98	1	0.99	0.02
FAS+Patel	0.97	1	1	0.99	1	0.96	0.98	1	0.99	0.02
FAS+Skew	0.97	1	1	0.99	1	0.96	0.98	1	0.99	0.02
FAS+RSkew	0.97	1	1	0.99	1	0.96	0.98	1	0.99	0.02
FASK	0.97	0.89	1	0.98	0.99	0.96	0.91	0.96	0.96	0.04
Two-Step(FAS)	0.91	0.91	0.91	0.91	0.91	0.91	0.91	0.91	0.91	0.00
Two-Step(Alasso)	1	1	1	0.96	0.98	0.98	1	1	0.99	0.02

Table C15: Adjacency Precision (average)

	Net7_amp	Net7_cont	Net8_a_a	Net8_a_c	Net8_c_a	Net9_a_a	Net9_a_c	Net9_c_a
MVGC	0.08	0.04	0.07	0.07	0.06	0.07	0.06	0.07
MVARp	0.09	0.04	0.10	0.09	0.08	0.08	0.07	0.06
GIMME-50	0.08	0.03	0.08	0.06	0.04	0.07	0.06	0.05
GIMME-60	0.08	0	0.07	0.06	0.03	0.07	0.06	0.05
GlobalMIT(FAS)	0.04	0	0.02	0	0	0.02	0	0
CCD-max	0.08	0	0.04	0.04	0.01	0.08	0.04	0
FAS+R1	0.08	0	0.04	0.04	0.01	0.08	0.04	0
FAS+R2	0.08	0	0.04	0.04	0.01	0.08	0.04	0
FAS+R3	0.08	0	0.04	0.04	0.01	0.08	0.04	0
FAS+Patel	0.08	0	0.04	0.04	0.01	0.08	0.04	0
FAS+Skew	0.08	0	0.04	0.04	0.01	0.08	0.04	0
FAS+Rskew	0.08	0	0.04	0.04	0.01	0.08	0.04	0
FASK	0.06	0.07	0.01	0.04	0.03	0.05	0.06	0.05
Two-Step(FAS)	0	0	0	0	0	0.01	0	0
Two-Step(Alasso)	0	0	0.02	0.05	0.04	0.06	0.01	0.02

Table C16: Adjacency Precision (standard deviation)

	Net7_amp	Net7_cont	Net8_a_a	Net8_a_c	Net8_c_a	Net9_a_a	Net9_a_c	Net9_c_a	average	std.dev
MVGC	1	1	1	1	1	1	1	1	1	0.00
MVARp	1	0.99	1	1	1	1	1	1	1	0.00
GIMME-50	1	0.99	1	1	0.98	1	1	1	1	0.01
GIMME-60	0.99	0.95	0.99	0.96	0.90	0.99	0.98	0.98	0.97	0.03
GlobalMIT(FAS)	0.66	0.57	0.70	0.64	0.48	0.68	0.65	0.63	0.63	0.07
CCD-max	1	1	1	1	1	1	1	1	1	0.00
FAS+R1	1	1	1	1	1	1	1	1	1	0.00
FAS+R2	1	1	1	1	1	1	1	1	1	0.00
FAS+R3	1	1	1	1	1	1	1	1	1	0.00
FAS+Patel	1	1	1	1	1	1	1	1	1	0.00
FAS+Skew	1	1	1	1	1	1	1	1	1	0.00
FAS+RSkew	1	1	1	1	1	1	1	1	1	0.00
FASK	1	1	1	1	1	1	1	1	1	0.00
Two-Step(FAS)	0.91	0.91	0.91	0.91	0.91	0.91	0.91	0.91	0.91	0.00
Two-Step(Alasso)	1	1	1	1	1	1	1	1	1	0.00

Table C17: Adjacency Recall (average)

	Net7_amp	Net7_cont	Net8_a_a	Net8_a_c	Net8_c_a	Net9_a_a	Net9_a_c	Net9_c_a
MVGC	0	0	0	0	0.01	0	0	0
MVARp	0	0.05	0	0	0	0	0.02	0.01
GIMME-50	0	0.04	0.01	0.01	0.04	0.01	0	0
GIMME-60	0.04	0.08	0.02	0.07	0.08	0.03	0.04	0.04
GlobalMIT(FAS)	0.12	0.12	0.09	0.07	0.09	0.1	0.09	0.08
CCD-max	0	0	0	0	0	0	0	0
FAS+R1	0	0	0	0	0	0	0	0
FAS+R2	0	0	0	0	0	0	0	0
FAS+R3	0	0	0	0	0	0	0	0
FAS+Patel	0	0	0	0	0	0	0	0
FAS+Skew	0	0	0	0	0	0	0	0
FAS+Rskew	0	0	0	0	0	0	0	0
FASK	0	0	0	0	0	0	0	0
Two-Step(FAS)	0	0	0	0	0	0	0	0
Two-Step(Alasso)	0	0	0	0	0	0	0	0.01

Table C18: Adjacency Recall (standard deviation)

	Net7_amp	Net7_cont	Net8_a_a	Net8_a_c	Net8_c_a	Net9_a_a	Net9_a_c	Net9_c_a	average	std.dev
MVGC	0.42	0.5	0.4	0.45	0.47	0.4	0.47	0.48	0.46	0.03
MVARp	0.49	0.52	0.5	0.49	0.47	0.4	0.51	0.5	0.49	0.02
GIMME-50	0.64	0.6	0.6	0.66	0.57	0.6	0.61	0.64	0.62	0.03
GIMME-60	0.66	0.61	0.6	0.69	0.57	0.7	0.63	0.65	0.63	0.04
GlobalMIT(FAS)	0.76	0.89	0.8	0.8	0.81	0.8	0.84	0.84	0.81	0.04
CCD-max	0.84	0.59	0.7	0.7	0.64	0.8	0.89	0.8	0.74	0.11
FAS+R1	1	0.97	1	0.97	0.98	1	0.96	0.88	0.97	0.04
FAS+R2	0.97	1	1	0.99	0.99	1	0.99	1	0.99	0.01
FAS+R3	0.97	0.98	1	0.94	0.98	1	0.92	0.9	0.96	0.03
FAS+Patel	0.55	0.41	0.5	0.53	0.56	0.4	0.43	0.48	0.48	0.06
FAS+Skew	0.96	1	1	0.99	1	1	0.98	1	0.99	0.02
FAS+RSkew	0.97	0.83	1	0.88	0.89	1	0.89	0.9	0.92	0.06
FASK	0.97	0.74	1	0.87	0.88	0.96	0.82	0.86	0.89	0.08
Two-Step(FAS)	0.87	0.75	0.9	0.78	0.84	0.8	0.74	0.74	0.80	0.06
Two-Step(Alasso)	0.76	0.69	0.7	0.7	0.72	0.7	0.69	0.74	0.71	0.03

Table C19: Orientation Precision (average)

	Net7_amp	Net7_cont	Net8_a_a	Net8_a_c	Net8_c_a	Net9_a_a	Net9_a_c	Net9_c_a
MVGC	0.03	0.01	0	0.03	0.02	0	0.03	0.02
MVARp	0.04	0.08	0	0.05	0.05	0	0.04	0.04
GIMME-50	0.22	0.15	0.2	0.16	0.13	0.2	0.18	0.14
GIMME-60	0.23	0.17	0.2	0.15	0.17	0.2	0.18	0.16
GlobalMIT(FAS)	0.15	0.14	0.1	0.12	0.14	0.1	0.11	0.1
CCD-max	0.25	0.03	0	0.06	0.32	0.2	0.06	0.03
FAS+R1	0.14	0.11	0.1	0.09	0.08	0.2	0.12	0.05
FAS+R2	0.08	0.07	0	0.04	0.03	0.1	0.07	0.04
FAS+R3	0.08	0.05	0	0.07	0.04	0.1	0.07	0.07
FAS+Patel	0.1	0.09	0.1	0.08	0.09	0.1	0.07	0.06
FAS+Skew	0.09	0	0	0.04	0.01	0.1	0.04	0
FAS+Rskew	0.08	0	0	0.03	0.01	0.1	0.03	0
FASK	0.06	0.07	0	0.04	0.03	0.1	0.06	0.05
Two-Step(FAS)	0.07	0.08	0.1	0.05	0.06	0.1	0.04	0.05
Two-Step(Alasso)	0.12	0.06	0.1	0.08	0.09	0.1	0.06	0.06

Table C20: Orientation Precision (standard deviation)

	Net7_amp	Net7_cont	Net8_a_a	Net8_a_c	Net8_c_a	Net9_a_a	Net9_a_c	Net9_c_a	average	std.dev
MVGC	1	0.99	1	0.99	0.98	1	1	0.99	0.99	0.01
MVARp	0.96	0.82	0.94	0.92	0.88	0.94	0.96	0.92	0.92	0.05
GIMME-50	0.73	0.64	0.68	0.73	0.58	0.77	0.67	0.71	0.69	0.06
GIMME-60	0.72	0.58	0.66	0.71	0.52	0.74	0.65	0.68	0.66	0.07
GlobalMIT(FAS)	0.62	0.56	0.66	0.6	0.46	0.65	0.64	0.62	0.6	0.07
CCD-max	0.84	0.49	0.66	0.7	0.64	0.84	0.9	0.8	0.73	0.14
FAS+R1	1	0.98	1	0.97	0.98	0.94	0.94	0.79	0.95	0.07
FAS+R2	0.83	0.88	0.89	0.87	0.88	0.89	0.86	0.85	0.87	0.02
FAS+R3	1	0.98	1	0.95	0.98	0.99	0.94	0.9	0.97	0.03
FAS+Patel	0.57	0.41	0.48	0.54	0.56	0.44	0.44	0.48	0.49	0.06
FAS+Skew	0.99	1	0.99	1	1	1	1	1	1	0.00
FAS+RSkew	1	0.83	1	0.89	0.89	1	0.9	0.9	0.93	0.07
FASK	1	0.83	1	0.89	0.89	1	0.91	0.9	0.93	0.06
Two-Step(FAS)	0.91	0.91	0.91	0.91	0.91	0.91	0.91	0.91	0.91	0.00
Two-Step(Alasso)	1	1	1	1	1	1	1	1	1	0.00

Table C21: Orientation Recall (average)

	Net7_amp	Net7_cont	Net8_a_a	Net8_a_c	Net8_c_a	Net9_a_a	Net9_a_c	Net9_c_a
MVGC	0.02	0.03	0	0.02	0.04	0	0.01	0.03
MVARp	0.09	0.16	0.08	0.1	0.11	0.08	0.06	0.09
GIMME-50	0.22	0.17	0.19	0.15	0.14	0.14	0.19	0.15
GIMME-60	0.22	0.17	0.18	0.17	0.15	0.16	0.17	0.16
GlobalMIT(FAS)	0.13	0.13	0.09	0.11	0.1	0.12	0.1	0.09
CCD-max	0.23	0.02	0.05	0.03	0.34	0.16	0.04	0.03
FAS+R1	0	0.05	0	0.05	0.07	0.09	0.1	0.06
FAS+R2	0.02	0.08	0.02	0.05	0.04	0.03	0.07	0.06
FAS+R3	0	0.05	0	0.07	0.04	0.03	0.06	0.07
FAS+Patel	0.1	0.09	0.1	0.08	0.09	0.07	0.07	0.06
FAS+Skew	0.03	0	0.02	0	0	0	0	0
FAS+Rskew	0	0	0	0	0	0	0	0
FASK	0	0	0	0	0	0	0	0
Two-Step(FAS)	0	0	0	0	0	0	0	0
Two-Step(Alasso)	0	0	0	0	0	0.02	0	0.02

Table C22: Orientation Recall (standard deviation)

	Net7_amp	Net7_cont	Net8_a_a	Net8_a_c	Net8_c_a	Net9_a_a	Net9_a_c	Net9_c_a	average	std.dev
MVGC	6.57	5.88	9.27	8.92	9.15	10.65	9.95	9.6	8.75	1.66
MVARp	4.75	3.47	7.25	6.62	7.03	7.7	7.43	7.32	6.45	1.52
GIMME-50	0.22	0.37	0.32	0.37	0.2	0.37	0.33	0.43	0.33	0.08
GIMME-60	0.03	0.05	0.03	0.05	0	0.12	0.08	0.03	0.05	0.04
GlobalMIT(FAS)	0.92	0.35	1.07	0.92	0.82	1.58	1.12	1.07	0.98	0.34
CCD-max	0	0	0	0	0.02	0.02	0	0	0.01	0.01
FAS+R1	0	0.23	0	0.23	0.13	0.35	0.33	1.07	0.29	0.34
FAS+R2	*	*	*	*	*	*	*	*	*	*
FAS+R3	*	*	*	*	*	*	*	*	*	*
FAS+Patel	*	*	*	*	*	*	*	*	*	*
FAS+Skew	*	*	*	*	*	*	*	*	*	*
FAS+RSkew	*	*	*	*	*	*	*	*	*	*
FASK	0.00	0.00	0.00	0.03	0.00	0.00	0.07	0.02	0.01	0.02
Two-Step (FAS)	0.59	1.48	0.86	1.6	0.96	1.56	2.26	2.18	1.44	0.60
Two-Step(Alasso)	2.03	2.72	3.53	3.7	3.58	4.67	4.62	3.48	3.54	0.88

Table C23: 2-cycle False Positives (average). The (*) symbol indicate algorithms that cannot detect 2-cycles by design: R2, R3, Patel, Skew and RSkew.

	Net7_amp	Net7_cont	Net8_a_a	Net8_a_c	Net8_c_a	Net9_a_a	Net9_a_c	Net9_c_a
MVGC	0.67	0.32	0.73	0.72	0.68	0.88	0.83	0.59
MVARp	1.04	1.03	1.47	1.52	1.45	1.43	1.06	1.19
GIMME-50	0.42	0.52	0.6	0.58	0.4	0.55	0.48	0.65
GIMME-60	0.18	0.22	0.18	0.22	0	0.32	0.28	0.18
GlobalMIT(FAS)	0.59	0.52	0.55	0.65	0.75	0.85	0.85	0.69
CCD-max	0	0	0	0	0.13	0.18	0	0
FAS+R1	0	0.65	0	0.43	0.34	0.7	0.51	0.36
FAS+R2	*	*	*	*	*	*	*	*
FAS+R3	*	*	*	*	*	*	*	*
FAS+Patel	*	*	*	*	*	*	*	*
FAS+Skew	*	*	*	*	*	*	*	*
FAS+Rskew	*	*	*	*	*	*	*	*
FASK	0.00	0.00	0.00	0.18	0.00	0.00	0.25	0.13
Two-Step (FAS)	0.46	0.76	0.7	0.65	0.68	0.9	0.64	0.71
Two-Step(Alasso)	1.21	0.78	1.27	1.28	1.37	1.5	1.19	1.11

Table C24: 2-cycle False Positives (standard deviation). The (*) symbol indicates algorithms that cannot detect 2-cycles by design: R2, R3, Patel, Skew and RSkew.

	Net7_amp	Net7_cont	Net8_a_a	Net8_a_c	Net8_c_a	Net9_a_a	Net9_a_c	Net9_c_a	average	std.dev
MVGC	0.13	0.13	0.19	0.19	0.18	0.27	0.22	0.23	0.19	0.05
MVARp	22.44	23.27	39.6	41.59	40.68	68.35	62.22	57.48	44.5	17.05
GIMME-50	156.8	159.4	166.6	129.6	120.4	220.1	201.2	173.2	165.9	33.27
GIMME-60	53.8	49.6	120.0	118.9	112.1	209.1	189.6	186.6	130.0	60.75
GlobalMIT(FAS)	0.89	0.65	1.38	1.36	1.29	2.22	1.89	1.88	1.45	0.53
CCD-max	0.00	0.00	0.00	0.00	0.00	0.00	0.00	0.00	0.00	0.00
FAS+R1	0.15	0.13	0.22	0.32	0.21	0.28	0.26	0.25	0.23	0.06
FAS+R2	1.49	1.09	2.34	3.04	2.30	3.63	3.16	2.95	2.50	0.87
FAS+R3	0.12	0.11	0.23	0.17	0.17	0.21	0.19	0.19	0.17	0.04
FAS+Patel	0.02	0.01	0.05	0.03	0.03	0.05	0.03	0.03	0.03	0.01
FAS+Skew	0.01	0.01	0.04	0.02	0.02	0.04	0.02	0.02	0.02	0.01
FAS+RSkew	0.02	0.01	0.05	0.02	0.02	0.05	0.03	0.03	0.03	0.01
FASK	0.03	0.01	0.07	0.05	0.04	0.09	0.06	0.05	0.05	0.02
Two-Step(FAS)	9.54	10.23	11.61	14.23	13.53	17.03	19.16	21.96	14.66	4.40
Two-Step(Alasso)	19.96	18.71	30.18	36.24	28.87	42.01	33.68	42.11	31.47	8.91

Table C25: Running Time (seconds) (average)

	Net7_amp	Net7_cont	Net8_a_a	Net8_a_c	Net8_c_a	Net9_a_a	Net9_a_c	Net9_c_a
MVGC	0.01	0.01	0.02	0.02	0.01	0.03	0.02	0.02
MVARp	1.19	1.51	2.22	2.57	4.66	11.02	5.07	6.15
GIMME-50	43.60	48.55	66.84	16.74	13.94	24.83	22.59	19.31
GIMME-60	6.08	4.84	12.60	12.03	14.46	27.42	18.21	14.83
GlobalMIT(FAS)	0.42	0.06	0.27	0.42	0.18	0.54	0.26	0.18
CCD-max	0.00	0.00	0.00	0.00	0.00	0.00	0.00	0.00
FAS+R1	0.03	0.00	0.02	0.03	0.01	0.06	0.03	0.00
FAS+R2	1.08	0.02	1.01	1.04	0.22	2.72	1.08	0.02
FAS+R3	1.08	0.02	1.01	1.04	0.22	2.72	1.08	0.02
FAS+Patel	0.00	0.00	0.00	0.00	0.00	0.00	0.00	0.00
FAS+Skew	0.00	0.00	0.00	0.00	0.00	0.00	0.00	0.00
FAS+RSkew	0.00	0.00	0.00	0.00	0.00	0.00	0.00	0.00
FASK	0.01	0.00	0.02	0.01	0.01	0.02	0.01	0.02
Two-Step(FAS)	4.58	5.48	4.61	5.70	8.38	14.09	6.33	8.94
Two-Step(Alasso)	3.35	2.54	3.63	6.54	6.37	8.30	5.47	15.32

Table C26: Running Time (seconds) (standard deviation)

C3 Macaque-based networks (standard deviation)

FASK (standard deviation)

Network	AP	AR	OP	OR	2CP	2CR	r.time(s)
SmallDegree	0.03	0.03	0.05	0.05	0.09	0.14	1.98
LongRange	0.04	0.02	0.03	0.02	0.06	0.07	0.31
LongRangeControl	0.04	0.02	0.04	0.02	0.09	0.06	0.13
Full	0.04	0.00	0.03	0.00	0.02	0.01	11.42

Two-Step(Alasso) (standard deviation)

Network	AP	AR	OP	OR	2CP	2CR	r.time(s)
SmallDegree	0.05	0.04	0.03	0.05	0.03	0.16	10.71
LongRange	0.01	0.03	0.01	0.03	0.02	0.06	3.20
LongRangeControl	0.02	0.03	0.02	0.03	0.04	0.05	7.61
Full	0.02	0.01	0.02	0.01	0.03	0.03	26.83

Table C27: **Standard deviation** over 60 repetitions of 10 concatenated datasets. Results for FASK and Two-Step with Alasso for the four different macaque-based networks. Precision and recall for adjacencies (AP, AR), orientations (OP, OR), 2-cycles (2CP, 2CR), and running times in seconds (r.time).

C4 Results for resting state medial temporal lobe data

C4.1 Left hemisphere results

Results for 23 repetitions of ten concatenated subjects, for seven regions of interest of the medial temporal lobe in the left hemisphere. For FASK and Two-Step(Alasso) we report the frequency of appearance in percentage, of each edge across the 23 repetitions. A value of 1.00 indicates that the edge shows in all 23 repetitions. A value of 0.04 indicates that the edge shows in only one of the repetitions. FASK was run with penalty $c = 1$ and $\alpha = 0.05$; Two-Step(Alasso) was run with $\lambda = 2$ and threshold 0.10.

**CA1**	→	**SUB**	**1.00**	**PHC**	→	**ERC**	**0.65**	BA36	→	CA1	0.26
**CA23DG**	→	**CA1**	**1.00**	**ERC**	→	**BA35**	**0.52**	ERC	→	SUB	0.22
**SUB**	→	**CA1**	**1.00**	**CA1**	→	**BA36**	**0.48**	CA1	→	PHC	0.17
**BA36**	→	**BA35**	**1.00**	**SUB**	→	**PHC**	**0.48**	CA23DG	→	SUB	0.17
**CA1**	→	**CA23DG**	**0.96**	SUB	→	CA23DG	0.43	ERC	→	PHC	0.17
**SUB**	→	**ERC**	**0.91**	BA35	→	PHC	0.43	CA1	→	BA35	0.09
**BA35**	→	**BA36**	**0.91**	SUB	→	BA35	0.26	CA23DG	→	ERC	0.09
**PHC**	→	**SUB**	**0.83**	BA35	→	SUB	0.26	PHC	→	CA1	0.09
**PHC**	→	**BA35**	**0.78**	BA35	→	ERC	0.26	BA35	→	CA1	0.04

Table C28: FASK: medial temporal lobe edges ordered by frequency of appearance (left hemisphere). In bold the edges with frequency equal to 48% or more.

**CA1**	→	**BA36**	**0.74**	**ERC**	→	**PHC**	**0.48**	ERC	→	CA1	0.30
**BA35**	→	**BA36**	**0.70**	**PHC**	→	**CA1**	**0.48**	ERC	→	CA23DG	0.30
**CA1**	→	**CA23DG**	**0.65**	ERC	→	BA35	0.43	PHC	→	BA35	0.30
**CA23DG**	→	**SUB**	**0.65**	BA36	→	CA23DG	0.43	CA1	→	PHC	0.26
**SUB**	→	**CA1**	**0.65**	BA36	→	CA1	0.39	CA23DG	→	PHC	0.26
**BA35**	→	**ERC**	**0.65**	BA36	→	SUB	0.39	BA35	→	CA1	0.26
**PHC**	→	**SUB**	**0.65**	CA1	→	ERC	0.35	PHC	→	CA23DG	0.22
**CA1**	→	**SUB**	**0.61**	BA35	→	PHC	0.35	BA35	→	CA23DG	0.17
**ERC**	→	**SUB**	**0.61**	BA36	→	PHC	0.35	BA35	→	SUB	0.17
**PHC**	→	**ERC**	**0.61**	PHC	→	BA36	0.35	SUB	→	CA23DG	0.13
**SUB**	→	**ERC**	**0.57**	CA1	→	BA35	0.30	BA36	→	ERC	0.13
**SUB**	→	**BA35**	**0.52**	CA23DG	→	ERC	0.30	CA23DG	→	BA36	0.04
**BA36**	→	**BA35**	**0.52**	SUB	→	BA36	0.30				
**CA23DG**	→	**CA1**	**0.48**	SUB	→	PHC	0.30				

Table C29: Two-Step(Alasso): medial temporal lobe edges ordered by frequency of appearance (left hemisphere). In bold the edges with frequency equal to 48% or more.

C4.2 Right hemisphere results

Results for 23 repetitions of ten concatenated subjects, for seven regions of interest of the medial temporal lobe in the right hemisphere. Figure C1 shows graphs depicting robust directed edges that appear in 48% or more of the 23 repetitions, for FASK and Two-Step(Alasso). For FASK and Two-Step(Alasso) we also report the frequency of appearance in percentage, of each edge across the 23 repetitions. A value of 1.00 indicates that the edge shows in all 23 repetitions. A value of 0.04 indicates that the edge shows in only one of the repetitions. FASK was run with penalty $c = 1$ and $\alpha = 0.05$; Two-Step(Alasso) was run with $\lambda = 2$ and threshold 0.10.

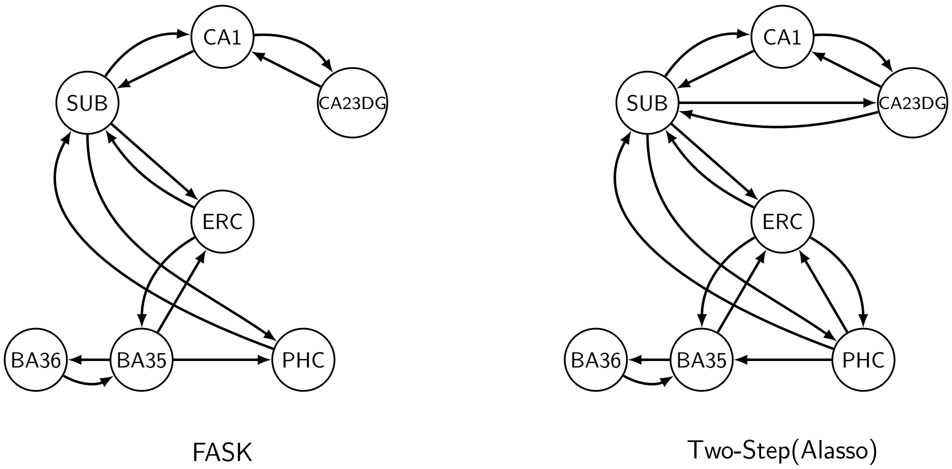

Figure C1: Comparison of the most robust edges estimated by FASK and Two-Step(Alasso) in 23 repetitions of ten concatenated subjects for seven regions of interest from the right hemisphere medial temporal lobe. A directed edge is depicted if it is estimated in 48% or more of the 23 repetitions of ten subjects concatenated. Regions of interest include: Cornu Ammonis 1 (CA1); CA2, CA3 and dentate gyrus together (CA23DG); entorhinal cortex (ERC); perirhinal cortex divided in Brodmann areas (BA35 and BA36); and parahippocampal cortex (PHC).

**CA1**	→	**CA23DG**	**1.00**	**BA35**	→	**ERC**	**0.87**	BA36	→	CA1	0.17
**CA1**	→	**SUB**	**1.00**	**BA35**	→	**PHC**	**0.87**	SUB	→	CA23DG	0.13
**CA23DG**	→	**CA1**	**1.00**	**PHC**	→	**SUB**	**0.83**	BA36	→	CA23DG	0.09
**SUB**	→	**ERC**	**1.00**	**SUB**	→	**PHC**	**0.70**	CA1	→	BA36	0.04
**ERC**	→	**SUB**	**1.00**	CA1	→	PHC	0.39	CA23DG	→	BA36	0.04
**BA36**	→	**BA35**	**1.00**	PHC	→	CA1	0.39	BA35	→	SUB	0.04
**SUB**	→	**CA1**	**0.96**	CA23DG	→	SUB	0.26	PHC	→	ERC	0.04
**ERC**	→	**BA35**	**0.96**	PHC	→	BA35	0.26				
**BA35**	→	**BA36**	**0.96**	ERC	→	PHC	0.17				

Table C30: FASK: medial temporal lobe edges ordered by frequency of appearance (right hemisphere). In bold the edges with frequency equal to 48% or more.

**CA1**	→	**SUB**	**0.78**	**CA23DG**	→	**SUB**	**0.48**	SUB	→	BA36	0.17
**CA1**	→	**CA23DG**	**0.74**	**SUB**	→	**PHC**	**0.48**	BA36	→	CA23DG	0.17
**CA23DG**	→	**CA1**	**0.74**	**ERC**	→	**PHC**	**0.48**	BA36	→	PHC	0.17
**ERC**	→	**SUB**	**0.70**	CA1	→	PHC	0.39	CA23DG	→	ERC	0.13
**SUB**	→	**CA1**	**0.65**	CA23DG	→	BA36	0.35	ERC	→	CA23DG	0.13
**BA35**	→	**BA36**	**0.65**	BA35	→	PHC	0.35	CA1	→	BA35	0.09
**PHC**	→	**ERC**	**0.65**	PHC	→	CA1	0.35	BA35	→	CA23DG	0.09
**SUB**	→	**ERC**	**0.61**	SUB	→	BA35	0.30	BA35	→	SUB	0.09
**BA35**	→	**ERC**	**0.61**	ERC	→	BA36	0.26	BA36	→	CA1	0.09
**SUB**	→	**CA23DG**	**0.57**	BA36	→	ERC	0.26	ERC	→	CA1	0.04
**BA36**	→	**BA35**	**0.57**	CA1	→	BA36	0.22	BA35	→	CA1	0.04
**PHC**	→	**SUB**	**0.57**	CA23DG	→	PHC	0.22	BA36	→	SUB	0.04
**ERC**	→	**BA35**	**0.52**	PHC	→	BA36	0.22				
**PHC**	→	**BA35**	**0.52**	CA1	→	ERC	0.17				

Table C31: Two-Step(Alasso): medial temporal lobe edges by frequency of appearance (right hemisphere). In bold the edges with frequency equal to 48% or more.

C4.3 2-cycles frequency of appearance

For FASK and Two-Step(Alasso) we report 2-cycles by frequency of appearance in percentage, across the 23 repetitions of ten concatenated subjects, for seven regions of interest from the medial temporal lobe for the left and right hemisphere correspondingly. A value of 1.00 indicates that the 2-cycle appears in all 23 repetitions. A value of 0.04 indicates that the 2-cycle appears in only one repetition. FASK was run with penalty $c = 1$ and $\alpha = 0.05$; Two-Step(Alasso) was run with $\lambda = 2$ and threshold 0.10.

left hemisphere				right hemisphere			
CA1	⇄	SUB	1.00	CA1	⇄	CA23DG	1.00
CA1	⇄	CA23DG	0.96	SUB	⇄	ERC	1.00
BA35	⇄	BA36	0.91	CA1	⇄	SUB	0.96
SUB	⇄	PHC	0.43	BA35	⇄	BA36	0.96
SUB	⇄	BA35	0.26	ERC	⇄	BA35	0.83
BA35	⇄	PHC	0.22	SUB	⇄	PHC	0.70
CA1	⇄	BA36	0.13	CA1	⇄	PHC	0.22
SUB	⇄	ERC	0.13	BA35	⇄	PHC	0.13
ERC	⇄	BA35	0.04	CA23DG	⇄	BA36	0.04
				ERC	⇄	PHC	0.04

Table C32: FASK: 2-cycles by frequency of appearance for medial temporal lobe regions of interest.

left hemisphere				right hemisphere			
CA1	⇄	BA36	0.30	CA1	⇄	CA23DG	0.48
ERC	⇄	PHC	0.30	ERC	⇄	PHC	0.48
CA1	⇄	SUB	0.26	CA1	⇄	SUB	0.43
CA1	⇄	ERC	0.26	CA23DG	⇄	SUB	0.35
BA36	⇄	PHC	0.26	SUB	⇄	ERC	0.30
SUB	⇄	ERC	0.22	SUB	⇄	PHC	0.22
ERC	⇄	BA35	0.22	BA35	⇄	BA36	0.22
BA35	⇄	BA36	0.22	ERC	⇄	BA35	0.17
CA1	⇄	BA35	0.17	ERC	⇄	BA36	0.17
CA23DG	⇄	PHC	0.17	CA23DG	⇄	BA36	0.13
SUB	⇄	BA36	0.17	BA36	⇄	PHC	0.13
CA1	⇄	CA23DG	0.13	CA23DG	⇄	ERC	0.09
CA1	⇄	PHC	0.13	CA1	⇄	ERC	0.04
CA23DG	⇄	SUB	0.13	CA1	⇄	PHC	0.04
CA23DG	⇄	ERC	0.13	BA35	⇄	PHC	0.04
SUB	⇄	BA35	0.13				
SUB	⇄	PHC	0.13				
BA35	⇄	PHC	0.09				
CA23DG	⇄	BA36	0.04				

Table C33: Two-Step(Alasso): 2-cycles by frequency of appearance for medial temporal lobe regions of interest.

C4.4 Individual datasets results for medial temporal lobe data

Results for 23 individual subjects datasets, for seven regions of interest of the medial temporal lobe in the left and right hemisphere correspondingly. For FASK and Two-Step(Alasso) we report the frequency of appearance in percentage, of each edge across the 23 individual subjects. A value of 1.00 indicates that the edge shows in all 23 subjects. A value of 0.04 indicates that the edge shows in only one of the subjects. We also report frequency of appearance for 2-cycles. FASK was run with penalty $c = 1$ and $\alpha = 0.10$; Two-Step(Alasso) was run with $\lambda = 2$ and threshold 0.10.

CA1	→	SUB	0.57	ERC	→	PHC	0.22	BA35	→	CA23DG	0.09
SUB	→	CA1	0.57	BA36	→	PHC	0.22	BA35	→	SUB	0.09
BA35	→	BA36	0.57	PHC	→	BA36	0.22	BA36	→	CA23DG	0.09
CA23DG	→	CA1	0.52	CA23DG	→	ERC	0.17	BA36	→	ERC	0.09
CA1	→	CA23DG	0.43	BA35	→	PHC	0.17	CA1	→	ERC	0.04
BA36	→	BA35	0.39	BA36	→	CA1	0.17	CA1	→	PHC	0.04
SUB	→	ERC	0.35	BA36	→	SUB	0.17	CA23DG	→	SUB	0.04
ERC	→	SUB	0.35	PHC	→	BA35	0.17	CA23DG	→	PHC	0.04
PHC	→	ERC	0.30	CA1	→	BA35	0.13	SUB	→	CA23DG	0.04
ERC	→	CA23DG	0.26	CA1	→	BA36	0.13	SUB	→	BA35	0.04
BA35	→	ERC	0.26	ERC	→	BA36	0.13	SUB	→	BA36	0.04
PHC	→	SUB	0.26	CA23DG	→	BA35	0.09	BA35	→	CA1	0.04
SUB	→	PHC	0.22	CA23DG	→	BA36	0.09	PHC	→	CA1	0.04
ERC	→	BA35	0.22	ERC	→	CA1	0.09	PHC	→	CA23DG	0.04

Table C34: FASK: medial temporal lobe edges by frequency of appearance (individual datasets) (left hemisphere).

CA1	→	CA23DG	0.57	SUB	→	CA23DG	0.39	BA35	→	CA23DG	0.30
SUB	→	CA1	0.57	ERC	→	BA36	0.39	BA36	→	CA1	0.30
ERC	→	CA23DG	0.57	BA36	→	ERC	0.39	CA1	→	PHC	0.26
ERC	→	SUB	0.57	CA1	→	BA35	0.35	ERC	→	PHC	0.26
BA35	→	BA36	0.57	CA23DG	→	CA1	0.35	BA35	→	PHC	0.26
PHC	→	BA35	0.57	CA23DG	→	BA35	0.35	BA36	→	PHC	0.26
BA36	→	BA35	0.52	SUB	→	ERC	0.35	PHC	→	CA1	0.26
PHC	→	SUB	0.52	SUB	→	BA35	0.35	PHC	→	CA23DG	0.26
CA1	→	SUB	0.48	BA36	→	SUB	0.35	PHC	→	BA36	0.26
CA1	→	BA36	0.48	CA23DG	→	ERC	0.30	CA23DG	→	SUB	0.17
CA1	→	ERC	0.43	CA23DG	→	BA36	0.30	CA23DG	→	PHC	0.13
SUB	→	PHC	0.43	SUB	→	BA36	0.30	BA35	→	ERC	0.13
ERC	→	BA35	0.43	ERC	→	CA1	0.30	BA36	→	CA23DG	0.13
PHC	→	ERC	0.43	BA35	→	CA1	0.30	BA35	→	SUB	0.04

Table C35: Two-Step(Alasso): medial temporal lobe edges by frequency of appearance (individual datasets) (left hemisphere).

CA23DG	→	CA1	0.78	CA23DG	→	SUB	0.17	PHC	→	CA23DG	0.09
CA1	→	SUB	0.65	SUB	→	CA23DG	0.17	CA1	→	ERC	0.04
CA1	→	CA23DG	0.61	BA36	→	CA23DG	0.17	CA1	→	BA35	0.04
BA36	→	BA35	0.52	BA36	→	ERC	0.17	CA23DG	→	ERC	0.04
ERC	→	SUB	0.48	BA36	→	PHC	0.17	CA23DG	→	PHC	0.04
ERC	→	BA35	0.48	CA23DG	→	BA36	0.13	SUB	→	BA35	0.04
BA35	→	PHC	0.43	PHC	→	ERC	0.13	ERC	→	CA23DG	0.04
SUB	→	ERC	0.39	PHC	→	BA35	0.13	ERC	→	BA36	0.04
BA35	→	ERC	0.39	PHC	→	BA36	0.13	BA35	→	CA1	0.04
BA35	→	BA36	0.35	CA23DG	→	BA35	0.09	BA35	→	CA23DG	0.04
SUB	→	CA1	0.30	SUB	→	PHC	0.09	BA35	→	SUB	0.04
ERC	→	PHC	0.30	BA36	→	CA1	0.09	BA36	→	SUB	0.04
PHC	→	SUB	0.26	PHC	→	CA1	0.09				

Table C36: FASK: medial temporal lobe edges by frequency of appearance (individual datasets) (right hemisphere).

BA36	→	BA35	0.65	ERC	→	SUB	0.39	ERC	→	CA23DG	0.26
SUB	→	ERC	0.61	BA35	→	CA23DG	0.39	ERC	→	PHC	0.26
CA1	→	CA23DG	0.52	BA36	→	CA1	0.39	BA36	→	ERC	0.26
CA23DG	→	CA1	0.52	CA1	→	ERC	0.35	BA36	→	PHC	0.26
BA35	→	ERC	0.52	CA1	→	BA35	0.35	PHC	→	CA1	0.26
BA35	→	PHC	0.52	CA1	→	PHC	0.35	PHC	→	ERC	0.26
CA23DG	→	SUB	0.48	CA23DG	→	BA35	0.35	SUB	→	BA35	0.22
CA23DG	→	ERC	0.48	PHC	→	SUB	0.35	BA35	→	CA1	0.22
SUB	→	CA1	0.48	PHC	→	BA35	0.35	PHC	→	CA23DG	0.22
ERC	→	BA35	0.48	PHC	→	BA36	0.35	BA36	→	SUB	0.17
BA35	→	BA36	0.48	CA23DG	→	PHC	0.30	SUB	→	BA36	0.13
CA1	→	SUB	0.43	ERC	→	BA36	0.30	ERC	→	CA1	0.13
SUB	→	PHC	0.43	CA23DG	→	BA36	0.26	BA35	→	SUB	0.13
BA36	→	CA23DG	0.43	SUB	→	CA23DG	0.26	CA1	→	BA36	0.09

Table C37: Two-Step(Alasso): medial temporal lobe edges by frequency of appearance (individual datasets) (right hemisphere).

left hemisphere				right hemisphere			
CA1	⇄	CA23DG	0.22	CA1	⇄	CA23DG	0.48
CA1	⇄	SUB	0.22	SUB	⇄	ERC	0.26
BA35	⇄	BA36	0.22	BA35	⇄	BA36	0.26
ERC	⇄	PHC	0.17	ERC	⇄	BA35	0.22
SUB	⇄	ERC	0.13	CA1	⇄	SUB	0.17
CA23DG	⇄	ERC	0.09	CA23DG	⇄	SUB	0.09
ERC	⇄	BA35	0.09	CA23DG	⇄	BA36	0.09
BA36	⇄	PHC	0.09	SUB	⇄	PHC	0.09
CA1	⇄	ERC	0.04	BA35	⇄	PHC	0.09
CA1	⇄	BA36	0.04	BA36	⇄	PHC	0.09
CA23DG	⇄	BA35	0.04	CA23DG	⇄	ERC	0.04
CA23DG	⇄	BA36	0.04	CA23DG	⇄	PHC	0.04
CA23DG	⇄	PHC	0.04	ERC	⇄	BA36	0.04
SUB	⇄	BA35	0.04	ERC	⇄	PHC	0.04
SUB	⇄	BA36	0.04				
SUB	⇄	PHC	0.04				
ERC	⇄	BA36	0.04				

Table C38: FASK: 2-cycles by frequency of appearance for medial temporal lobe regions of interest (individual data).

left hemisphere				right hemisphere			
CA1	⇄	BA35	0.17	BA35	⇄	BA36	0.26
CA1	⇄	BA36	0.17	CA23DG	⇄	ERC	0.17
CA23DG	⇄	ERC	0.17	CA23DG	⇄	BA35	0.17
SUB	⇄	ERC	0.17	SUB	⇄	ERC	0.13
ERC	⇄	BA36	0.17	SUB	⇄	PHC	0.13
BA35	⇄	BA36	0.17	ERC	⇄	BA35	0.13
CA1	⇄	SUB	0.13	CA1	⇄	CA23DG	0.09
CA1	⇄	ERC	0.13	CA1	⇄	ERC	0.09
CA23DG	⇄	BA35	0.13	CA1	⇄	BA35	0.09
SUB	⇄	PHC	0.13	CA23DG	⇄	SUB	0.09
CA1	⇄	PHC	0.09	CA23DG	⇄	PHC	0.09
SUB	⇄	BA36	0.09	SUB	⇄	BA35	0.09
CA1	⇄	CA23DG	0.04	BA35	⇄	PHC	0.09
CA23DG	⇄	SUB	0.04	CA1	⇄	SUB	0.04
CA23DG	⇄	PHC	0.04	CA1	⇄	BA36	0.04
ERC	⇄	BA35	0.04	CA1	⇄	PHC	0.04
BA35	⇄	PHC	0.04	CA23DG	⇄	BA36	0.04
				ERC	⇄	BA36	0.04
				BA36	⇄	PHC	0.04

Table C39: Two-Step(Alasso): 2-cycles by frequency of appearance for medial temporal lobe regions of interest (individual data).

C5 Results for rhyming task individual data

FASK and Two-Step(Alasso) were run on 9 individual subjects standardized datasets (160 datapoints) for eight regions of interest in the cerebral cortex and one Input variable modeling the dynamics of the visual stimuli presentation in the rhyming task. We report frequency of appearance in percentage, of individual edges across the 9 subjects. We also report 2-cycles frequency of appearance. A value of 1.00 indicates the edge appears in all the 9 subjects. A value of 0.11 indicates the edge appears in just one subject. FASK was run with a penalty discount of $c = 1$ and $\alpha = 0.10$ to account for the reduction in sample size relative to the

concatenated data (1,440 datapoints). Two-Step(Alasso) was run with $\lambda = 2$ and threshold for the absolute values of the **B** matrix of 0.10.

LOCC	→	ROCC	0.89	LIPL	→	LIFG	0.22	RACC	→	LIPL	0.11
LACC	→	RACC	0.78	RIFG	→	LIFG	0.22	RACC	→	RIPL	0.11
ROCC	→	LOCC	0.67	RIFG	→	RACC	0.22	RACC	→	Input	0.11
LIPL	→	RIPL	0.56	LOCC	→	LIFG	0.11	RIFG	→	RIPL	0.11
LIFG	→	LIPL	0.44	LOCC	→	LIPL	0.11	RIFG	→	Input	0.11
LIFG	→	RIFG	0.44	LACC	→	Input	0.11	RIPL	→	RIFG	0.11
RACC	→	LACC	0.44	LIFG	→	LACC	0.11	Input	→	LOCC	0.11
RIPL	→	LIPL	0.44	LIPL	→	LACC	0.11	Input	→	LACC	0.11
LOCC	→	Input	0.22	LIPL	→	RACC	0.11	Input	→	RACC	0.11
LACC	→	LIFG	0.22	ROCC	→	RIPL	0.11				

Table C40: FASK: Rhyming task data edges ordered by frequency of appearance (individual datasets).

ROCC	→	LOCC	0.89	LACC	→	LIPL	0.33	LOCC	→	RIPL	0.11
LOCC	→	ROCC	0.78	LIFG	→	LACC	0.33	LOCC	→	Input	0.11
LACC	→	RACC	0.78	LIPL	→	LACC	0.33	LACC	→	RIPL	0.11
LIFG	→	RIFG	0.78	LIPL	→	RIFG	0.33	LIFG	→	ROCC	0.11
Input	→	LOCC	0.78	RIFG	→	RIPL	0.33	LIFG	→	RIPL	0.11
LOCC	→	LIPL	0.67	Input	→	LIFG	0.33	LIFG	→	Input	0.11
LIFG	→	LIPL	0.67	Input	→	RIFG	0.33	LIPL	→	LOCC	0.11
LIPL	→	RIPL	0.67	LIFG	→	LOCC	0.22	LIPL	→	RACC	0.11
RACC	→	RIPL	0.56	LIFG	→	RACC	0.22	ROCC	→	LACC	0.11
RIFG	→	LIFG	0.56	ROCC	→	RACC	0.22	ROCC	→	LIFG	0.11
RIPL	→	RIFG	0.56	ROCC	→	RIPL	0.22	RACC	→	LOCC	0.11
LACC	→	LIFG	0.44	RACC	→	LACC	0.22	RIFG	→	LACC	0.11
LIPL	→	LIFG	0.44	RACC	→	LIFG	0.22	RIFG	→	LIPL	0.11
ROCC	→	RIFG	0.44	RACC	→	ROCC	0.22	RIPL	→	LOCC	0.11
RACC	→	RIFG	0.44	RIFG	→	ROCC	0.22	RIPL	→	LACC	0.11
RIFG	→	RACC	0.44	RIPL	→	ROCC	0.22	RIPL	→	RACC	0.11
RIPL	→	LIPL	0.44	Input	→	LACC	0.22	Input	→	RACC	0.11
LOCC	→	LACC	0.33	Input	→	LIPL	0.22				
LOCC	→	LIFG	0.33	Input	→	ROCC	0.22				

Table C41: Two-Step(Alasso): Rhyming task data edges ordered by frequency of appearance (individual datasets).

LOCC	⇄	ROCC	0.56
LACC	⇄	RACC	0.33
LIPL	⇄	RIPL	0.33
LIFG	⇄	RIFG	0.11
LIPL	⇄	RACC	0.11

Table C42: FASK: Rhyming task data 2-cycles ordered by frequency of appearance (individual datasets).

LOCC	⇄	ROCC	0.67
LIFG	⇄	RIFG	0.44
LIFG	⇄	LIPL	0.22
LOCC	⇄	LIFG	0.11
LACC	⇄	LIPL	0.11
LIFG	⇄	ROCC	0.11
LIFG	⇄	RACC	0.11
LIPL	⇄	RIPL	0.11
RACC	⇄	RIFG	0.11
RACC	⇄	RIPL	0.11
RIFG	⇄	RIPL	0.11

Table C43: Two-Step(Alasso): Rhyming task data 2-cycles ordered by frequency of appearance (individual datasets).

C6 Measurement noise and sampling resolution results

Values used to compute percentage changes reported in Figures 4 and 5 of the main text, for data with and without measurement noise (abbreviated as m.n) and a fast sampling rate of TR = 1.2 seconds, and a slower rate of TR = 3 seconds. Table C44 shows average results over 60 repetitions of 10 concatenated datasets from Network 4, for precision and recall of adjacencies (AP, AR), orientations (OP, OR) and 2-cycle detection (2CP, 2CR), and running times in seconds. Corresponding standard deviations are presented in table C45.

FASK (average)

Network 4	AP	AR	OP	OR	2CP	2CR	r.time(s)
BOLD (m.n. // 1.2 s TR)	0.97	0.85	0.97	0.88	1.00	1.00	0.24
BOLD (without m.n // 1.2 s TR)	0.98	0.86	0.98	0.88	1.00	0.94	0.19
BOLD (m.n // 3 s TR)	0.94	0.80	0.95	0.84	1.00	1.00	0.26

Two-Step(Alasso) (average)

Network 4	AP	AR	OP	OR	2CP	2CR	r.time(s)
BOLD (m.n. // 1.2 s TR)	0.92	0.80	0.73	0.71	0.64	0.86	64.53
BOLD (without m.n // 1.2 s TR)	0.99	0.91	0.99	0.93	0.97	1.00	17.52
BOLD (m.n // 3 s TR)	0.90	0.80	0.76	0.75	0.68	0.88	76.68

MVGC (average)

Network 4	AP	AR	OP	OR	2CP	2CR	r.time(s)
BOLD (m.n. // 1.2 s TR)	0.70	0.95	0.52	0.90	0.33	1.00	0.65
BOLD (without m.n // 1.2 s TR)	0.53	0.67	0.50	0.56	0.66	0.43	0.71
BOLD (m.n // 3 s TR)	0.91	0.51	0.87	0.51	0.99	0.68	0.29

MVARp (average)

Network 4	AP	AR	OP	OR	2CP	2CR	r.time(s)
BOLD (m.n. // 1.2 s TR)	0.60	0.93	0.43	0.72	0.44	0.95	61.29
BOLD (without m.n // 1.2 s TR)	0.35	0.68	0.23	0.48	0.22	0.49	79.94
BOLD (m.n // 3 s TR)	0.47	0.84	0.37	0.66	0.42	0.74	84.37

GlobalMIT(FAS) (average)

Network 4	AP	AR	OP	OR	2CP	2CR	r.time(s)
BOLD (m.n. // 1.2 s TR)	0.99	0.46	0.89	0.50	0.97	0.93	4.66
BOLD (without m.n // 1.2 s TR)	1.00	0.38	0.94	0.42	0.97	0.63	2.91
BOLD (m.n // 3 s TR)	1.00	0.18	0.99	0.14	NaN	0.00	3.20

Table C44: Precision and recall average results over 60 repetitions of 10 concatenated datasets, for Network 4 under different conditions of sampling resolution (1.2 and 3 seconds TR) and measurement noise (m.n or without m.n).

FASK (standard deviation)

Network 4	AP	AR	OP	OR	2CP	2CR	r.time(s)
BOLD (m.n. // 1.2 s TR)	0.04	0.06	0.04	0.05	0.03	0.00	0.15
BOLD (without m.n // 1.2 s TR)	0.04	0.06	0.03	0.05	0.03	0.12	0.18
BOLD (m.n // 3 s TR)	0.06	0.06	0.04	0.04	0.00	0.00	0.16

Two-Step(Alasso) (standard deviation)

Network 4	AP	AR	OP	OR	2CP	2CR	r.time(s)
BOLD (m.n. // 1.2 s TR)	0.06	0.08	0.09	0.10	0.14	0.15	7.77
BOLD (without m.n // 1.2 s TR)	0.02	0.04	0.04	0.04	0.07	0.00	8.05
BOLD (m.n // 3 s TR)	0.07	0.07	0.08	0.07	0.15	0.16	14.76

MVGC (standard deviation)

Network 4	AP	AR	OP	OR	2CP	2CR	r.time(s)
BOLD (m.n. // 1.2 s TR)	0.06	0.05	0.04	0.06	0.04	0.00	0.07
BOLD (without m.n // 1.2 s TR)	0.11	0.13	0.13	0.12	0.32	0.23	0.09
BOLD (m.n // 3 s TR)	0.11	0.12	0.08	0.09	0.05	0.21	0.05

MVARp (standard deviation)

Network 4	AP	AR	OP	OR	2CP	2CR	r.time(s)
BOLD (m.n. // 1.2 s TR)	0.10	0.07	0.07	0.08	0.08	0.10	1.87
BOLD (without m.n // 1.2 s TR)	0.05	0.13	0.05	0.14	0.13	0.24	2.15
BOLD (m.n // 3 s TR)	0.08	0.09	0.08	0.09	0.16	0.19	10.80

GlobalMIT(FAS) (standard deviation)

Network 4	AP	AR	OP	OR	2CP	2CR	r.time(s)
BOLD (m.n. // 1.2 s TR)	0.03	0.07	0.08	0.05	0.08	0.12	1.92
BOLD (without m.n // 1.2 s TR)	0.02	0.06	0.06	0.06	0.10	0.22	1.07
BOLD (m.n // 3 s TR)	0.02	0.08	0.05	0.07	NaN	0.00	1.62

Table C45: Precision and recall standard deviation over 60 repetitions of 10 concatenated datasets, for Network 4 under different conditions of sampling resolution (1.2 and 3 seconds TR) and measurement noise (m.n or without m.n).

Supplementary Material D

Spectral density of simulated neural signals

The neural signals z (from Eq. 1 in the main text, $dz/dt = \sigma \mathbf{A} z + \mathbf{C} u$) come from a Poisson process that controls two states with mean duration of 2.5 seconds (up) and 10 s (down) together with the connectivity matrix $\mathbf{A}$. We plot (overlapped) spectral density estimates (left) and corresponding log-log plots (right), from 91 synthetic neural signals from the full macaque network, with a black line representing the mean log spectral density. The mean log spectral density (black line in the log-log plot) was fitted to a power-law function $p(x) = x^{-a}$, which resulted in an exponent of $a = 2.4$. We consider that the spectral densities shown in our log-log plot resemble, in shape and mean exponent value to the log-log plots for spectral density of human electrocorticography (ECoG) reported in Fig. 1, Fig. 2, and Fig. S1 of He, B. J., et al. (2010). The temporal structures and functional significance of scale-free brain activity. *Neuron*, 66(3), 353-369.

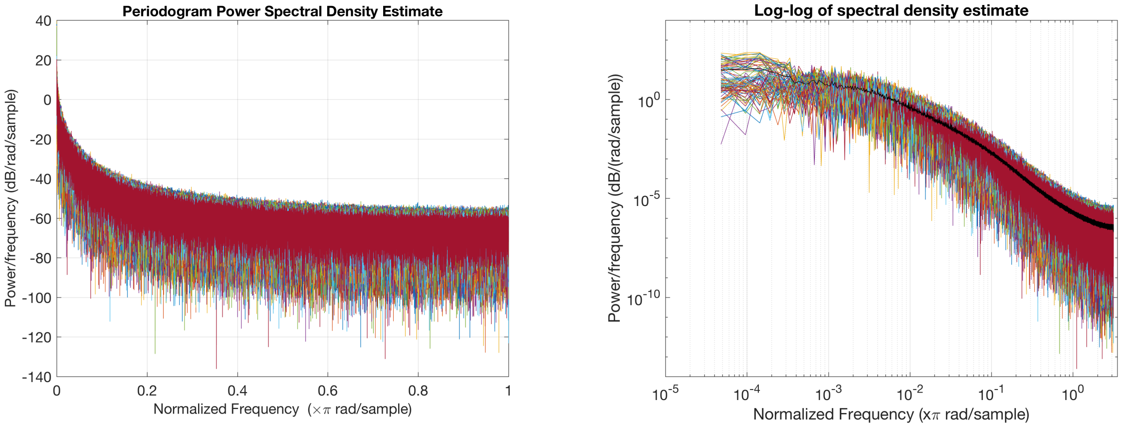

Figure D1: Spectral density estimates (left) and log-log plots (right) for simulated neural signals of 91 nodes from the full macaque network. The black line in the log-log plot represent the mean spectral density estimates. The mean log spectral density was fitted to a power-law function $p(x) = x^{-a}$, which resulted in an exponent of $a = 2.4$.

Supplementary Material E

Performance at increasing sample size

To analyze the dependency of the performance of FASK and Two-Step to the sample size, we ran FASK and Two-Step(Alasso) at increasing sample size (obtained by concatenating datasets) for the *Network 4* simulation. The minimum sample size tested was 500, and the maximum 30,000. FASK was run with a value of $c = 2$ for the penalty discount and $\alpha = 10^{-6}$ for the 2-cycle detection. Two-Step(Alasso) with a $\lambda = 32$ and a threshold of 0.15 for the absolute values of the estimated **B** matrix.

Con.Data	Sample Size	**AP**	**AR**	**OP**	**OR**	**2CP**	**2CR**	**2CFP**	**2CFN**	**2CTP**	**elapsed (s)**
1	500	0.89	0.53	0.91	0.53	1	0.50	0	2.00	2.00	0.0
2	1,000	1	0.53	1	0.58	1	0.75	0	1	3.00	0.0
3	1,500	1	0.60	1	0.63	1	0.75	0	1	3.00	0.0
4	2,000	1	0.73	1	0.79	1	1	0	0	4.00	0.1
5	2,500	1	0.73	1	0.79	1	1	0	0	4.00	0.1
10	5,000	1	0.93	1	0.95	1	1	0	0	4.00	0.3
15	7,500	1	1	1	1	1	1	0	0	4.00	0.5
20	10,000	1	1	1	1	1	1	0	0	4.00	0.8
25	12,500	1	1	1	1	1	1	0	0	4.00	0.9
30	15,000	0.94	1	0.95	1	1	1	0	0	4.00	1.3
35	17,500	0.94	1	0.95	1	1	1	0	0	4.00	1.7
40	20,000	0.94	1	0.95	1	1	1	0	0	4.00	1.8
45	22,500	0.94	1	0.95	1	1	1	0	0	4.00	2.3
50	25,000	0.94	1	0.95	1	1	1	0	0	4.00	2.3
55	27,500	0.94	1	0.95	1	1	1	0	0	4.00	2.5
60	30,000	0.94	1	0.95	1	1	1	0	0	4.00	4.4

Table E1: **Performance of FASK at increasing sample size**. FASK was run with $c = 2$ and $\alpha = 10^{-6}$. AP, AR adjacency precision and recall; OP, OR orientation precision and recall; 2CP, 2CR 2-cycle precision and recall; 2CFP, 2CFN and 2CTP 2-cycle false positive, false negative and true positive; elapsed time in seconds. Con.Data column indicates number of concatenated datasets.

Con.Data	Sample Size	**AP**	**AR**	**OP**	**OR**	**2CP**	**2CR**	**2CFP**	**2CFN**	**2CTP**	**elapsed (s)**
1	500	0.77	0.67	0.50	0.42	0.67	0.50	1	2.00	2.00	7.1
2	1,000	0.93	0.87	0.63	0.53	1	0.50	0	2.00	2.00	11.4
3	1,500	0.82	0.93	0.63	0.63	1	0.50	0	2.00	2.00	12.1
4	2,000	0.85	0.73	0.65	0.58	0.75	0.75	1	1	3.00	22.9
5	2,500	0.88	0.93	0.67	0.74	0.60	0.75	2.00	1	3.00	28.2
10	5,000	0.79	0.73	0.65	0.68	0.33	0.50	4.00	2.00	2.00	58.1
15	7,500	0.92	0.73	0.76	0.68	0.60	0.75	2.00	1	3.00	70.7
20	10,000	1	0.67	0.72	0.68	0.50	1	4.00	0	4.00	118.5
25	12,500	1	0.67	0.72	0.68	0.50	1	4.00	0	4.00	117.9
30	15,000	1	0.73	0.81	0.68	0.60	0.75	2.00	1	3.00	137.0
35	17,500	1	0.67	0.75	0.63	0.50	0.75	3.00	1	3.00	176.7
40	20,000	1	0.67	0.72	0.68	0.50	1	4.00	0	4.00	205.8
45	22,500	1	0.67	0.71	0.63	0.57	1	3.00	0	4.00	226.0
50	25,000	1	0.80	0.70	0.74	0.50	1	4.00	0	4.00	250.6
55	27,500	1	0.80	0.68	0.68	0.57	1	3.00	0	4.00	261.9
60	30,000	1	0.80	0.67	0.63	0.67	1	2.00	0	4.00	289.6

Table E2: **Performance of Two-Step(Alasso) at increasing sample size.** Two-Step(Alasso) was run with $\lambda = 32$ and threshold of 0.15 for the **B** matrix. AP, AR adjacency precision and recall; OP, OR orientation precision and recall; 2CP, 2CR 2-cycle precision and recall; 2CFP, 2CFN and 2CTP 2-cycle false positive, false negative and true positive; elapsed time in seconds. Con.Data column indicates number of concatenated datasets.

Supplementary Material F

Subsampling strategy application

We apply the Bühlmann & van de Geer (2011, ch. 10) subsampling strategy for the control of false positives to our particular problem of inferring a cyclic directed network from fMRI data. This strategy consists in creating k random subsamples of size $n/2$ without replacement (within each subsample) from an original dataset of sample size n; infer k graphs and count the number of times a particular edge appears across the k graphs. A final graph is built with those edges that appear in more than a proportion t of the k graphs, where $0.50 < t \leq 1$. The value of t is a threshold —which may be different for each network and dataset —that depends on the number of tolerated false positives. To obtain t we first order the edges, from high to low, according to f, the proportion of times an edge appears across the k graphs. Then, we assign to each ordered edge an index q starting at $q = 1$ for the edge with the highest f. Finally for *each* q and f we compute $E(a) = (q^2/p)(1/(2f - 1))$, which is a bound on the expected number of false positives if we set the threshold $t = f$. In the above equation, $p = v(v - 1)$, where v is the number of nodes and p is the number of total possible directed edges in the network. Once we build this table, we can find the value of f corresponding to the value of $E(a)$ closest to the number of false positives we are tolerating, and set the threshold t equal to such f, as long as $0.50 < f \leq 1$. For example, if we tolerate a number of false positives equal to 5% of the total number of possible edges p, then we look in the column $E(a)$ of our table for the value closest to $0.05p$, and set t equal to the corresponding f. Intuitively, the smaller the number of false positives tolerated the higher the value of t that will be selected.

Using datasets with 500 datapoints, we test this subsampling strategy for FASK and Two-Step(Alasso) on the 18 simple simulations and in the four Macaque-based simulations. We compare precision and recall for these datasets against those obtained with the concatenated datasets of 5,000 datapoints for which results were previously reported in Figures 2, 3 and Table 1 in the main text. For the subsampling strategy we use $k = 100$ to guarantee stability of the results. In our simulations, for smaller size problems, such as the 18 simple networks, a number of false positives equal to 5% of the possible directed edges p, produces good results, but with higher size problems, such as the Macaque-based simulations, we obtained better results with a proportion of $1/p$.

For the datasets with 500 datapoints, we use FASK with a penalty of $c = 1$ and $\alpha = 0.01$ for the 18 simple simulations, and $\alpha = 0.10$ for the Macaque-based; Two-Step(Alasso) was run with $\lambda = 1$ and varying $\mathbf{B}$ matrix thresholds, 0.10 for the 18 simple simulations and *Macaque SmallDegree* network; 0.05 for the *Macaque LongRange* and *Macaque LongRangeControl* cases; and 0.005 for the *Macaque Full* network. These threshold values for Two-Step correspond to the ones derived previously in the parameter tuning process.

	AP		AR		OP		OR		2CP		2CR	
	**500**	**5,000**	**500**	**5,000**	**500**	**5,000**	**500**	**5,000**	**500**	**5,000**	**500**	**5,000**
Net1_amp	1	0.95	0.56	1	1	0.96	0.57	1	1	1	0.60	1
Net2_amp	0.98	0.85	0.62	1	0.98	0.88	0.49	1	1	1	0.15	0.99
Net3_amp	0.95	0.85	0.48	1	0.97	0.89	0.49	1	1	1	0.50	1
Net4_amp	0.96	0.97	0.43	0.85	0.97	0.97	0.47	0.88	1	1	0.63	1
Net5_amp	0.97	0.83	0.58	1	0.98	0.86	0.60	1	1	1	0.70	1
Net5_cont	0.98	0.68	0.68	1	0.98	0.70	0.62	0.93	1	0.90	0.40	0.67
Net5_c_p3n7	1	0.83	0.78	1	1	0.77	0.64	0.81	1	0.12	0.10	0.07
Net5_c_p7n3	0.98	0.75	0.65	0.99	0.98	0.78	0.52	0.97	0.00	1	0.00	0.92
Net6_amp	0.89	0.98	0.66	1	0.89	0.98	0.68	1	0.94	1	0.80	1
Net6_cont	0.97	0.77	0.80	1	0.97	0.79	0.75	1	1	1	0.40	1
Net7_amp	1	0.97	0.62	1	0.98	0.97	0.62	1				
Net7_cont	0.98	0.89	0.70	1	0.77	0.74	0.55	0.83				
Net8_a_a	1	1	0.63	1	1	1	0.63	1				
Net8_a_c	0.96	0.98	0.71	1	0.82	0.87	0.61	0.89				
Net8_c_a	0.96	0.99	0.78	1	0.87	0.88	0.70	0.89				
Net9_a_a	1	0.96	0.57	1	0.96	0.96	0.56	1				
Net9_a_c	0.93	0.91	0.70	1	0.77	0.82	0.60	0.91				
Net9_c_a	0.95	0.96	0.71	1	0.82	0.86	0.61	0.90				

Table F1: **Performance of FASK with subsampling strategy and 500 datapoints compared to performance with 5,000 datapoints, for the 18 simple simulations**. AP, AR indicate adjacency precision and recall; OP, OR indicate orientation precision and recall; 2CP, 2CR 2-cycle precision and recall. The empty rows correspond to networks without 2-cycles.

	AP		AR		OP		OR		2CP		2CR	
	**500**	**5,000**	**500**	**5,000**	**500**	**5,000**	**500**	**5,000**	**500**	**5,000**	**500**	**5,000**
SmallDegree	0.95	0.78	0.23	0.84	0.91	0.75	0.22	0.79	0.94	0.96	0.18	0.81
LongRange	0.24	0.95	0.01	0.24	0.19	0.95	0.01	0.31	0.00	0.94	0.00	0.81
LongRangeCont.	0.30	0.94	0.01	0.22	0.25	0.91	0.01	0.24	0.07	0.85	0.01	0.44
Full	0.23	0.98	0.00	0.01	0.10	0.99	0.00	0.02	0.00	1	0.00	0.07

Table F2: **Performance of FASK with subsampling strategy and 500 datapoints compared to performance with 5,000 datapoints, for the Macaque-based simulations**. AP, AR indicate adjacency precision and recall; OP, OR indicate orientation precision and recall; 2CP, 2CR 2-cycle precision and recall.

	AP		AR		OP		OR		2CP		2CR	
	**500**	**5,000**	**500**	**5,000**	**500**	**5,000**	**500**	**5,000**	**500**	**5,000**	**500**	**5,000**
Net1_amp	1	0.93	0.66	1	0.93	0.78	0.65	0.99	1	0.49	0.90	0.97
Net2_amp	0.95	0.76	0.68	1	0.87	0.72	0.54	0.96	0.92	0.68	0.35	0.93
Net3_amp	1	0.87	0.66	1	0.98	0.85	0.64	1	1	0.83	0.65	0.99
Net4_amp	0.89	0.92	0.62	0.8	0.69	0.73	0.61	0.71	0.60	0.64	0.95	0.86
Net5_amp	0.95	0.88	0.78	0.99	0.88	0.87	0.74	0.99	0.71	0.9	0.60	0.97
Net5_cont	0.88	0.88	0.68	0.85	0.73	0.79	0.54	0.69	0.00	0.03	0.00	0.03
Net5_cont_p3n7	0.93	0.76	0.78	1	0.79	0.70	0.66	0.8	0.29	0.02	0.20	0.02
Net5_cont_p7n3	0.92	1	0.88	1	0.88	0.93	0.70	0.8	0.00	0.02	0.00	0.02
Net6_amp	0.93	0.97	0.94	1	0.74	0.91	0.94	1	0.36	0.72	1	1
Net6_cont	0.79	0.88	0.81	0.89	0.57	0.82	0.71	0.79	0.00	0.16	0.00	0.2
Net7_amp	0.98	1	0.92	1	0.92	0.76	0.92	1				
Net7_cont	1	1	0.92	1	0.77	0.69	0.87	1				
Net8_a_a	0.97	1	0.94	1	0.73	0.72	0.92	1				
Net8_a_c	0.95	0.96	0.92	1	0.70	0.7	0.90	1				
Net8_c_a	1	0.98	0.97	1	0.78	0.72	0.96	1				
Net9_a_a	0.92	0.98	0.96	1	0.67	0.67	0.95	1				
Net9_a_c	0.94	1	0.89	1	0.61	0.69	0.84	1				
Net9_c_a	0.93	1	0.91	1	0.60	0.74	0.84	1				

Table F3: **Performance of Two-Step(Alasso) with subsampling strategy and 500 datapoints compared to performance with 5,000 datapoints, for the 18 simple simulations**. AP, AR indicate adjacency precision and recall; OP, OR indicate orientation precision and recall; 2CP, 2CR 2-cycle precision and recall. The empty rows correspond to networks without 2-cycles.

	AP		AR		OP		OR		2CP		2CR	
	**500**	**5,000**	**500**	**5,000**	**500**	**5,000**	**500**	**5,000**	**500**	**5,000**	**500**	**5,000**
SmallDegree	0.91	0.74	0.37	0.82	0.74	0.52	0.35	0.76	0.45	0.18	0.44	0.83
LongRange	0.32	0.97	0.10	0.84	0.32	0.97	0.09	0.85	0.50	0.99	0.04	0.93
LongRangeCont.	0.28	0.97	0.09	0.81	0.27	0.97	0.08	0.77	0.28	0.98	0.02	0.50
Full	0.28	0.88	0.01	0.27	0.26	0.82	0.01	0.28	0.21	0.77	0.01	0.46

Table F4: **Performance of Two-Step(Alasso) with subsampling strategy and 500 datapoints compared to performance with 5,000 datapoints, for the Macaque-based simulations**. AP, AR indicate adjacency precision and recall; OP, OR indicate orientation precision and recall; 2CP, 2CR 2-cycle precision and recall.
